# Anxiety in the Medically Ill: A Systematic Review of the Literature

**DOI:** 10.3389/fpsyt.2022.873126

**Published:** 2022-06-03

**Authors:** Sara Romanazzo, Giovanni Mansueto, Fiammetta Cosci

**Affiliations:** ^1^Department of Experimental and Clinical Medicine, University of Florence, Florence, Italy; ^2^Department of Health Sciences, University of Florence, Florence, Italy; ^3^Clinical Pharmacopsychology Laboratory, University of Florence, Florence, Italy; ^4^Department of Psychiatry and Neuropsychology, Maastricht University, Maastricht, Netherlands

**Keywords:** systematic review, anxiety, medically ill, disease, medical illness, rate, prevalence

## Abstract

**Background:**

Although anxiety is highly represented in the medically ill and its occurrence has relevant clinical implications, it often remains undetected and not properly treated. This systematic review aimed to report on anxiety, either symptom or disorder, in patients who suffer from a medical illness.

**Methods:**

English-language papers reporting on anxiety in medically ill adults were evaluated. PubMed, PsycINFO, Web of Science, and Cochrane databases were systematically searched from inception to June 2021. Search term was “anxiety” combined using the Boolean “AND” operator with “medically ill/chronic illness/illness/disorder/disease.” Risk of bias was assessed *via* the Joanna Briggs Institute (JBI) Critical Appraisal Tools—Checklist for Prevalence Studies. The PRISMA guidelines were followed.

**Results:**

Of 100,848 citations reviewed, 329 studies met inclusion criteria. Moderate or severe anxious symptoms were common among patients with cardiovascular, respiratory, central nervous system, gastrointestinal, genitourinary, endocrine, musculoskeletal system or connective tissue, dermatological diseases, cancer, AIDS and COVID-19 infections. The most common anxiety disorder was generalized anxiety disorder, observed among patients with cardiovascular, respiratory, central nervous system, dermatologic diseases, cancer, primary aldosteronism, amenorrhea, and COVID-19 infection. Panic disorder was described for cardiovascular, respiratory, dermatology diseases. Social anxiety was found for cardiovascular, respiratory, rheumatoid diseases. Specific phobias were relatively common in irritable bowel syndrome, gastroesophageal reflux, end-stage renal disease.

**Conclusion:**

Anxiety is a major challenge in medical settings. Recognition and proper assessment of anxiety in patients who suffer from a medical illness is necessary for an appropriate management. Future reviews are warranted in order also to clarify the causal and temporal relationship between anxiety and organic illness.

## Introduction

Anxiety is a feeling characterized by anguish, sense of threat, and fear. When it is explained by a real and objective trigger, it is considered physiological. When there are no objective reasons of being in such status, anxiety becomes pathological (1). For instance, when an organic disease occurs, anxiety can be a normal psychological reaction (2) but it can also flourish and evolve into a symptom with a pathological meaning or into a mental disorder. Anxiety, indeed, is highly represented in the medically ill (3), with generalized anxiety disorder as the most prevalent disorder (10.3%) in primary care settings (4). The pathways between anxiety and physical illness co-occurrence are not fully understood. Several possible mechanisms and synergies exist. Among them, environmental and genetic factors (5) have been proposed as being able to favor such co-occurrence as well as individual vulnerability (6) and socio-economic status (7).

In addition, anxiety may influence how the patient experiences the pathological process of own medical illness and his interaction with others (8, 9), including medical and nursing staff (10). In particular anxiety in association with a chronic medical illness worsens the quality of life (11), affects social functioning (12), increases medical burden (13). Anxiety has a negative impact on compliance (14), resulting in exacerbation of illness (15) and high health care utilization and costs (16). Anxiety increases the susceptibility to illness leading to illness progression, rehospitalization, and mortality (17–20). Among chronic illness patients, anxiety negatively affects emotional stability resulting in depressive symptoms, suicidal ideation, and social isolation (21, 22). Several studies reported the strong association between anxiety and somatization (23–25). In medically ill patients, anxiety amplifies physical symptoms, leading to useless (if not dangerous from a physical or psychological point of view) and inappropriate invasive tests/procedures to investigate hypothetical, but never confirmed, organic explanations (26). The treatment of anxiety, whether pharmacological or psychological, was found to favorably affect the outcome of a number of organic diseases (27, 28).

Unfortunately, anxiety often remains unacknowledged, unrecognized, untreated in medically ill patients (29, 30). Such a phenomenon is the result of several converging factors. First, the differentiation of anxiety worthy of clinical attention is hindered by the widespread occurrence of non-clinically relevant anxious symptoms in medical settings (31). Secondly, when an anxiety disorder is associated with a medical illness, there is a tendency to regard it as a physiological psychological reaction, secondary to the distress of the medical illness or to the patient's awareness of its consequences (32). However, in the clinical realm it is evident that not all medically ill patients have anxiety or an anxiety disorder (33). In addition, the expression of emotional distress is often disregarded or even discouraged by clinicians, and patients' needs are satisfied if they refer to the body rather than to the psychological sphere (34). Finally, the use of anxiolytics, particularly benzodiazepines, is widespread, especially during hospitalizations (35), often without a real indication. On the contrary the prescription seems justified by the organizational limits of hospital, being the staff able to handle only a limited number of requests.

Although anxiety is highly represented in the medically ill and its occurrence has relevant clinical implications, no systematic reviews on its prevalence and rate seem currently available. To fill this gap, the aim of the present systematic review was to report on anxiety, either symptom or disorder, in patients who suffer from a medical illness.

## Methods

### Registration

This review protocol was registered in the “International Prospective Register of Systematic Reviews” (PROSPERO) in 2021, under the registration number: CRD42021296741, and available at: https://www.crd.york.ac.uk/prospero/display_record.php?ID=CRD42021296741, and not published elsewhere.

### Eligibility Criteria

Eligible articles included English-language papers published in peer-reviewed journals reporting data on anxiety in medically ill adults. Anxiety disorder had to be diagnosed according to the Diagnostic and Statistical Manual of mental disorders (DSM-III, -III R, -IV, -IVTR or -5th edition) or the International Classification of Diseases (ICD-9, -10, -11). Anxious symptoms had to be assessed *via* standardized rating scales.

Additional inclusion criteria were: age of at least 18 years, a sample of at least 10 subjects (as it was already used in 36 in order to guarantee and minimum representativeness of results). Studies with different designs were included (i.e., cross-sectional, longitudinal study, observational, case-control studies).

Exclusion criteria were: (a) patients with multiple organic diagnoses, (b) not original data, (c) non-clinical samples (d) results on anxiety aggregated with results on depression or other psychological features. Treatment outcome studies were not included, being off topic for the present review.

### Information Sources and Search Strategy

The following electronic databases were systematically searched from inception to June 2021: PubMed, PsycINFO, Web Of Science, and Cochrane. In addition, a manual search of reference lists from relevant reviews was done. Search term was “anxiety” combined using the Boolean “AND” operator with “medically ill/chronic illness/illness/disorder/disease.”

### Selection and Data Collection Process, Data Items

Titles and abstracts were screened by two authors (S.R. and G.M.). Articles potentially relevant were retrieved and the authors independently assessed each in full. Any disagreement was resolved by consensus. Risk of bias (quality of the studies) was assessed *via* the Joanna Briggs Institute (JBI) Critical Appraisal Tools—Checklist for Prevalence Studies (36). It consists of 9 questions and the scoring system is: “yes” scores 1, “no” or “not clear” or “not applicable” score 0. The JBI Checklist-Total score is the sum of the items.

### Effect Measures

The search strings, the list of relevant reviews, the data coding, and the quality criteria are available on request to the corresponding author. No missing data were found. The methods described fulfilled the Preferred Reporting Items for Systematic Reviews and Meta-Analyses (PRISMA) guidelines (37).

## Results

**Figure 1:**
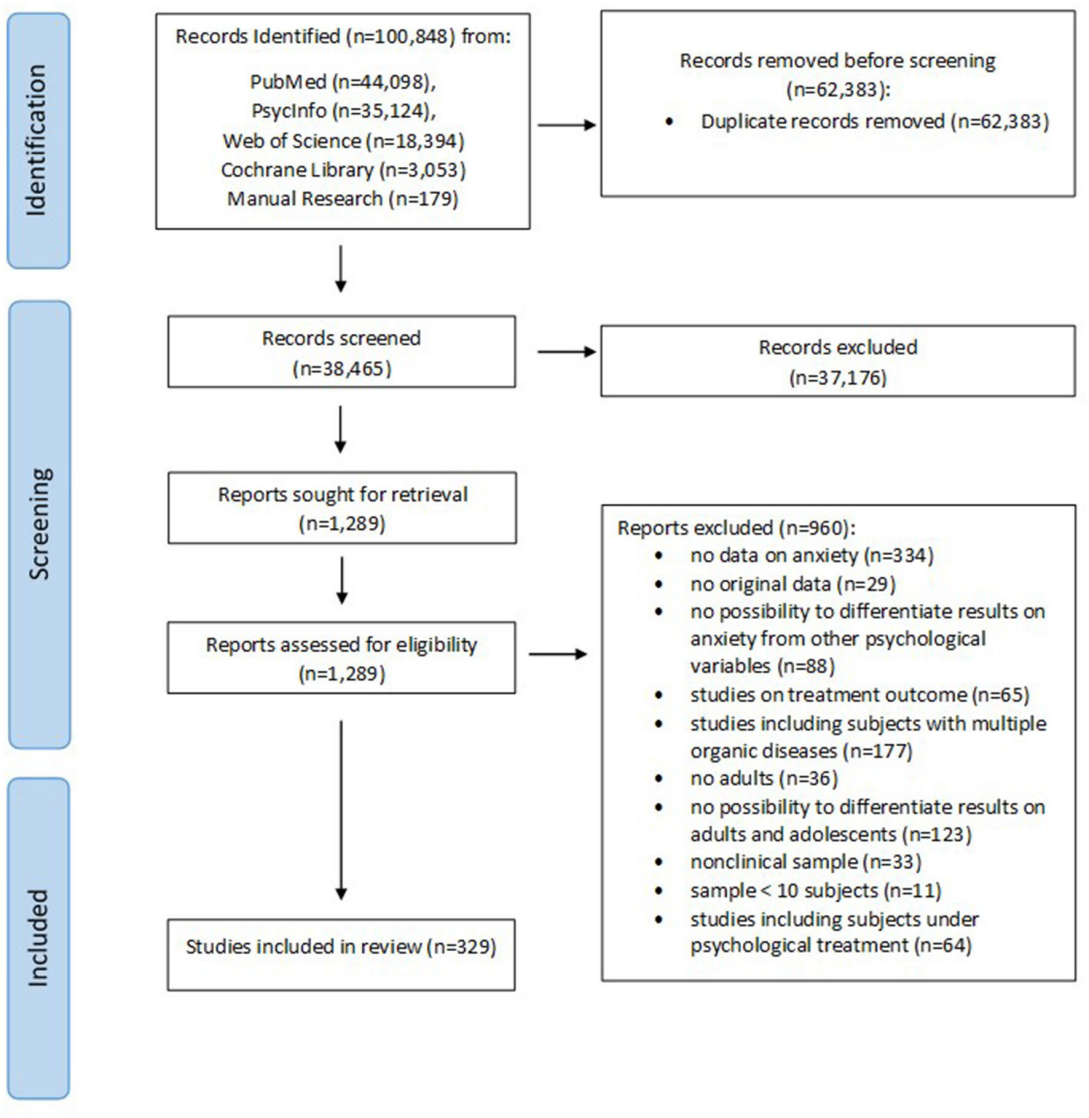
Flow-chart of study selection.

The search provided a total of 100,848 citations. After reviewing the abstracts to exclude those which clearly did not meet the criteria, 1,289 remained. Of these, 960 were excluded, not meeting the inclusion criteria (Figure 1 shows the flow diagram of the search). A total of 329 studies were identified for inclusion, the following parameters were extracted: country/city where data were collected, study design, organic disease diagnosis, sample size, instrument used to assess anxiety, eventual psychopathological manifestations co-occurring with anxiety (for details see Supplementary Table S1). Among them, 7 studies reached the maximum JBI score, 68 had a score of 8, 244 studies had a score of 7, and 10 studies had a score of 6, which means that at least 6 out of 9 criteria were fulfilled. Results will be here qualitatively presented based on the apparatus involved in the organic illness (see also Tables 1, 2). Symptoms severity levels were taken directly from respective studies and may refer to the use of different cut-offs for the same scale of assessment. No meta-analysis was conducted due to the methodological heterogeneity (i.e., tools used for assessment, cut-off choice) of the studies included.

**Table 1:** Moderate or severe anxiety symptoms in medical illness.

**Apparatus**	**Medical illness with moderate or severe anxious symptoms**
Cardiovascular	Atrial fibrillation, heart failure, myocardial infarction, coronary heart disease, coronary artery disease, coronary artery bypass graft, heart transplantation, acute chest pain
Respiratory	Asthma, chronic obstructive pulmonary disease, pulmonary hypertension, chronic thromboembolic pulmonary hypertension, chronic rhinosinusitis, incidental pulmonary nodules, interstitial lung diseases, obstructive sleep apnea
Central Nervous System	Epilepsy, multiple sclerosis, amyotrophic lateral sclerosis, Parkinson's disease, migraine, chronic pelvic pain, postherpetic neuralgia
Gastrointestinal	Chronic digestive system diseases, digestive system tumors, liver cirrhosis, chronic viral hepatitis, functional dyspepsia, inflammatory bowel disease, irritable bowel syndrome, hepatitis C, autoimmune hepatitis
Genitourinary	Chronic kidney disease, end-stage renal disease, chronic hemodialysis, kidney transplantation
Endocrine	Type 2 diabetes, hyperprolactinemia, obesity
Musculoskeletal system or connective tissue	Rheumatoid arthritis, systemic sclerosis, systemic lupus erythematosus, fibromyalgia, osteoarthritis, chronic foot and ankle disease, hip pathology
Dermatological	Psoriasis, chronic urticaria, hereditary angioedema, androgenetic alopecia
Cancer	Advanced cancer in palliative services, stomach cancer, advanced non-small cell lung cancer, breast cancer, metastatic breast cancer, prostate cancer, differentiated thyroid cancer, hematological cancer, chronic lymphocytic leukemia, Hodgkin lymphoma, head and neck cancer
Infections	AIDS, COVID-2019 infection
Miscellaneous	Systemic hypertension and type 2 diabetes, cystic fibrosis, neurofibromatosis type 1, otolaryngology diseases, orthotopic liver transplantation, benign breast lumps

*COVID-19: coronavirus disease 2019; HIV, human immunodeficiency virus; AIDS, acquired immunodeficiency syndrome*.

**Table 2:** Anxiety disorders in medical illness.

**Anxiety disorder**	**Medical illness**
Generalized anxiety disorder	Chronic heart failure, systemic arterial hypertension, myocardial infarction, Coronary heart disease, asthma, chronic obstructive pulmonary disease, stroke, multiple sclerosis, Parkinson's disease, chronic pain, gastroesophageal reflux disease, irritable bowel syndrome, chronic kidney disease, end-stage renal disease on hemodialysis treatment, endocrine disease, primary aldosteronism, systemic lupus erythematosus, fibromyalgia, Chronic low-back pain, chronic low back or neck pain caused by disc herniation, dermatological disease, early stage breast cancer, advanced breast cancer, prostate cancer, renal cell carcinoma, lymphoma, plasma cell dyscrasia, malignant melanoma patients, AIDS, tuberculosis, COVID-19 infection, amenorrhea
Panic disorder	Chronic heart failure, systemic arterial hypertension, myocardial infarction, coronary heart disease, angiographically normal or near normal coronary arteries, asthma, chronic obstructive pulmonary disease, pulmonary hypertension, multiple sclerosis, Parkinson's disease, chronic pain, hepatitis C, chronic kidney disease, end-stage renal disease, endocrine diseases, systemic lupus erythematosus, fibromyalgia, chronic low-back pain, dermatological diseases, early stage breast cancer, renal cell carcinoma, lymphoma, plasma cell dyscrasia, malignant melanoma patients, AIDS, tuberculosis
Agoraphobia	Systemic arterial hypertension, myocardial infarction, coronary heart disease, chronic obstructive pulmonary disease, gastroesophageal reflux disease, multiple sclerosis, chronic pain, irritable bowel syndrome, endocrine diseases, fibromyalgia, early stage breast cancer, advanced breast cancer, asthma, Parkinson's disease, systemic lupus erythematosus
Specific phobias	Systemic arterial hypertension, coronary heart disease, angiographically normal or near normal coronary arteries, multiple sclerosis, Parkinson's disease, chronic pain, gastroesophageal reflux disease, irritable bowel syndrome, end-stage renal disease on hemodialysis treatment, systemic lupus erythematosus, chronic low back or neck pain caused by disc herniation, early stage breast cancer, advanced breast cancer
Social anxiety disorder	Systemic arterial hypertension, coronary heart disease, Asthma, chronic obstructive pulmonary disease, gastroesophageal reflux disease, irritable bowel syndrome, multiple sclerosis, Parkinson's disease, systemic sclerosis, systemic lupus erythematosus, chronic pain, end-stage renal disease, chronic low back or neck pain, early stage breast cancer, advanced breast cancer
Anxiety disorder not otherwise specified	Parkinson's disease, end-stage renal disease on hemodialysis treatment, chronic low back or neck pain caused by disc herniation

*COVID-19, coronavirus disease 2019; AIDS, acquired immunodeficiency syndrome*.

### Cardiovascular Diseases

Anxious symptoms were found in 32.5–53.1% of patients with cardiovascular diseases (Carvalho et al., 2016^*^; Allabadi et al., 2019^*^); 33.9% had mild-to-moderate anxiety, 19.2% had severe to very severe anxiety (Allabadi et al., 2019^*^). A DSM-IV diagnosis of anxiety disorder was observed in 16% of cardiovascular disease patients (Dogar et al., 2008^*^). Women presented more anxiety than men (Dogar et al., 2008^*^; Carvalho et al., 2016^*^).

About 35% of atrial fibrillation patients showed high levels of anxiety, in particular women (49%). Patients having “bad” relations with nursing staff had 6.58 times higher possibility to experience anxiety than patients having “very good” relations (*p* < 0.05) (10^*^).

Patients with Heart Failure presented moderate anxiety (38) (Aggelopoulou et al., 2017^*^), with a rate of 24.9% of clinically relevant anxiety (Damen et al., 2012^*^). Among chronic heart failure patients, 11.4% had anxious symptoms (DSM-IV), with a negative association with self-care behavior (Müller-Tasch et al., 2017^*^). Eighteen per cent had at least one DSM-IV anxiety disorder: 11% generalized anxiety disorder (GAD), 8% panic disorder (PD), 2% both PD and GAD (Haworth et al., 2005^*^).

Among patients with systemic arterial hypertension, 36% had a DSM-IV anxiety disorder: 22.4% GAD, 4% specific phobia, 3.2% agoraphobia, 2.4% PD, 1.6% PD with agoraphobia, 1.6% social phobia (Rafanelli et al., 2012^*^).

Twenty-six percent of Myocardial Infarction (MI) patients had mild to moderate anxiety (Huffman et al., 2006^*^). Among patients after acute MI, 8.9% had anxiety within 24 h after primary percutaneous coronary intervention, 4.5% had anxiety after 3 months, 10.8% 6 months after the discharge, and 6.2% 12 months after (Kala et al., 2016^*^). In addition, 59.5% of patients had clinically relevant anxiety 1 month after the first MI, which was associated with an increased risk of adverse cardiac events (Strik et al., 2003^*^). Fourteen percent of patients with a first episode MI were affected by PD, 12% by agoraphobia, and 11% by GAD (DSM-IV) (Ottolini et al., 2005^*^).

Anxiety symptoms were found in 27–34.7% of Coronary Heart Disease patients before surgery for percutaneous coronary intervention (Gu et al., 2016^*^; Olsen et al., 2018^*^), anxiety significantly increased the day after surgery (54.7%) (Gu et al., 2016^*^) and decreased 3 months later (20.2%). Anxious symptoms were more represented among patients who attended in an exercise-based cardiac rehabilitation program compared to non-participants (Olsen et al., 2018^*^). Prevalence of DSM-IV anxiety disorders was 15% (Yang et al., 2014^*^). Social phobia and GAD were the most prevalent disorders (current prevalence: 20.3–21.3% and 17.5–24%, respectively), followed by specific phobia (14.0–14.7%), PD (4.7–4.9%), and agoraphobia (3.3–3.5%) (Bankier et al., 2004^*^; Todaro et al., 2007^*^; Serber et al., 2009^*^).

In patients with Coronary Artery Disease (CAD), anxiety was moderate (7–12%) or high (3–18.6%) (Wang et al., 2013a^*^; Ozturk et al., 2015^*^; Moryś et al., 2016^*^). Patients with anxiety were more likely to be female, older, unmarried, currently non-smoker and non-drinker, and with severer CAD (Wang et al., 2013a^*^). Anxiety was higher in case of the additional presence of Coronary Artery Ectasia (CAE) (Ozturk et al., 2015^*^); anxiety before and after coronary angiography was significantly higher in CAD patients than among CAE patients or among patients with both CAD and CAE (Ozturk et al., 2015^*^).

About 16–44% of patients with Coronary Artery Bypass Graft had clinically significant anxiety (Okamoto et al., 2013^*^; Guzelhan et al., 2018^*^) although it was more common before surgery (38.7%) and after hospital discharge (38.6%) (Gallagher and McKinley, 2009^*^).

Twenty-six percent of patients with congenital heart disease met DSM criteria for anxiety disorders (Kovacs et al., 2009^*^). Severe anxiety was observed in 9% of heart transplantation patients, especially if depressed (Kugler et al., 2014^*^). Among acute chest pain patients, 30% reported mild anxiety, 14–21.3% moderate, 15% severe (Schwarz et al., 2015^*^; Lin et al., 2021^*^).

Among patients with angiographically normal or near normal coronary arteries, 34% met criteria for current PD and 11% presented specific phobia (Beitman et al., 1989^*^).

About 37% of patients with sudden cardiac death undergoing implantable cardioverter defibrillator reported a DSM-IV-TR anxiety disorder (Gostoli et al., 2016^*^).

### Respiratory Diseases

From 11.8 to 51.5% of patients with asthma had clinically significant anxiety (Vamos and Kolbe, 1999^*^; Cordina et al., 2009^*^; Liu et al., 2014^*^), with more severe anxiety and more frequent anxiety disorders in uncontrolled asthma than in controlled asthma patients (Lomper et al., 2016^*^). Females had significantly higher anxiety than males (Cordina et al., 2009^*^). About 23–52.3% of patients with asthma met the diagnostic criteria for at least one DSM-IV anxiety disorder, the most common were: agoraphobia without PD (4.8–26.8%), PD (11–13.9%), GAD (5–24.4%), social anxiety disorder (3.2–9.3%), other anxiety disorders (10%) (Nascimento et al., 2002^*^; Lavoie et al., 2006^*^; Valença et al., 2006^*^).

Sixteen-thirty percent of Chronic Obstructive Pulmonary Disease (COPD) patients presented moderate anxiety, 57% mild to moderate anxiety, and 6.5–100% severe anxiety (Sutton et al., 1999^*^; Cleland et al., 2007^*^; Tang et al., 2008^*^; DiNicola et al., 2013^*^; Montserrat-Capdevila et al., 2017^*^; Thapa et al., 2017^*^; Kapisiz and Eker, 2018^*^; Vikjord et al., 2020^*^; Bove et al., 2020^*^; Lima et al., 2020^*^). Females had significantly higher rates or more severe anxiety than males (Tang et al., 2008^*^; Giardino et al., 2010^*^; Turan et al., 2014^*^; Xiao et al., 2018^*^; Vikjord et al., 2020^*^; Bove et al., 2020^*^). Anxiety was significantly associated with older age, increased dyspnea, reduced functional performance and quality of life (von Leupoldt et al., 2011^*^; Uchmanowicz et al., 2016^*^; An et al., 2021^*^). COPD patients presenting anxiety in the first year had a higher incidence of at least 2 exacerbations of COPD (41.3%) than patients without anxiety (26.4%), similarly in the second year of monitoring (Montserrat-Capdevila et al., 2017^*^).

Sixteen percent of patients with COPD had a DSM-III-R anxiety disorder. The combined prevalence rate for PD with or without agoraphobia was 8% (Karajgi et al., 1990^*^) while the prevalence of GAD and PD was 5.9% (Chan et al., 2009^*^), of GAD 7.3%, of PD 12.7%, of agoraphobia 7.3%, and of social anxiety 1.8% (Cheung et al., 2012^*^).

A rate of 48% of anxiety symptoms was observed in Pulmonary Hypertension (PH) patients (Vanhoof et al., 2014^*^). Mild anxiety was referred by 24.3%, moderate or severe anxiety by 6.8–20.5% of patients (Shafazand et al., 2004^*^; White et al., 2006^*^; Takita et al., 2021^*^) and severe anxiety by 32.9% (Pfeuffer et al., 2017^*^). The most common DSM-IV-TR anxiety disorder was PD (10.4%) while one fourth of patients had panic attacks. PD and panic attacks were significantly more common in PH patients compared with patients with inflammatory rheumatic disease (IRDs) or with primary care patients (Löwe et al., 2004^*^).

Severe anxiety was found in 43.5% of patients with chronic thromboembolic pulmonary hypertension (Pfeuffer et al., 2017^*^).

Among chronic rhinosinusitis (CRS) patients, 25.9% presented moderate anxiety and 25.9–49.5% severe anxiety (Wasan et al., 2007^*^; Vogt et al., 2021^*^). CRS as well as nasal septal deviation, allergic rhinitis, chronic respiratory disease, and steady-state bronchiectasis patients had higher anxiety than healthy subjects (Katotomichelakis et al., 2013^*^; Sharma et al., 2013b^*^; Gao et al., 2018^*^; Bekir et al., 2020^*^; Ma et al., 2020^*^). Clinically significant anxiety was also found in 31.8% of patients with incidental pulmonary nodules (Li et al., 2020b^*^) and in 12% of those with interstitial lung diseases (Holland et al., 2014^*^).

Among patients with obstructive sleep apnea, anxious symptoms were found in 73.6% of females vs. 53.4% of males; 21.2% of the total sample reported mild anxiety, 14.9% moderate, and 9.1% severe (Asghari, 2012^*^).

### Central Nervous System Diseases

The rate of anxiety among stroke patients was found to increase significantly between 6 months (17%) and 5 years after the event (29%) (Lincoln et al., 2013^*^). Point prevalence of DSM-III-R anxiety disorders was higher in the stroke survivors than in healthy controls. The most common diagnoses were: PD (24 vs. 8%) and GAD (27 vs. 8%). The prevalence of any anxiety disorder was higher in the stroke group (42 vs. 16%) than in healthy controls (Cumming et al., 2016^*^).

Clinically significant anxious symptoms were found in 24.5–33.8% of epilepsy patients (Asadi-Pooya and Sperling, 2011^*^; Alsaadi et al., 2015^*^; Lin and Pakpour, 2017^*^; Zhong et al., 2021^*^). Anxiety was significantly related to sex (31–43.7% of females vs. 16–28.6% of males) (de Souza and Salgado, 2006^*^; Asadi-Pooya and Sperling, 2011^*^) and to the type of epilepsy. Generalized epilepsy patients had significantly lower anxiety than those with temporal lobe epilepsy (Nenadović et al., 2011^*^).

Anxious symptoms were observed in 19.3–90% of Multiple Sclerosis (MS) patients (Noy et al., 1995^*^; Beiske et al., 2008^*^; Jones et al., 2012^*^; Alsaadi et al., 2015^*^; Henry et al., 2019; Wallis et al., 2019^*^; Hanna and Strober, 2020^*^). Females were more frequently anxious than males (Jones et al., 2012^*^). Among these patients, 21.1% had mild, 24.4–39.1% moderate, and 20.7–34.5% severe anxiety (Fruewald et al., 2001^*^; Korostil and Feinstein, 2007^*^; Askari et al., 2014^*^; Karimi et al., 2020^*^). A significantly higher level of anxious symptoms in relapsing remitting MS patients was found if compared with progressive MS patients (Henry et al., 2019^*^). Among MS patients reporting pain, anxiety was higher than among MS subjects without pain (Łabuz-Roszak et al., 2019^*^). Lifetime prevalence of DSM-IV anxiety disorders was 35.7%, point prevalence was 14–42% (GAD 10–18.6%, agoraphobia 8%; specific phobia 2.9–12%, social anxiety 2.1%, PD 2.1%) (Galeazzi et al., 2005^*^; Korostil and Feinstein, 2007^*^; Honarmand and Feinstein, 2009^*^).

Eighteen percent of amyotrophic lateral sclerosis patients had severe anxiety (Wicks et al., 2007^*^) as well as 13.3% of patients with spina bifida (Bellin et al., 2010^*^).

Parkinson's disease patients experienced anxiety (16–55%) (Menza et al., 1993^*^; Quelhas and Costa, 2009^*^; Zhu et al., 2017^*^; Cui et al., 2017^*^), 11% had severe anxiety (Mondolo et al., 2007^*^), 33% had moderate to severe anxiety (Quelhas and Costa, 2009^*^), 24% mild anxiety (Mondolo et al., 2007^*^). “Inability to relax,” “restlessness,” “frightened feeling,” experience of “worrying thoughts” and “feeling tense” were the most common manifestations of anxiety among Parkinson's disease patients (Mondolo et al., 2007^*^). Twenty-eight percent had a DSM-III-R anxiety disorder (PD 12%; GAD 12%; phobic disorder 2%; anxiety disorder, not otherwise specified 2%) (Menza et al., 1993^*^); 20–34% had a DSM-IV diagnosis of current anxiety disorder (agoraphobia without panic 15.5–15.6%, social anxiety 10–13%, PD with agoraphobia 8%, specific phobia 7.8%, PD 6.7%, PD with limited symptoms 1%, GAD 3–20.6%; anxiety disorder NOS was present in 26.7%) (Dissanayaka et al., 2010^*^; Leentjens et al., 2011^*^; Dissanayaka et al., 2016^*^) while 11.8–13.3% met the criteria for multiple DSM-IV anxiety disorders (Leentjens et al., 2011^*^; Dissanayaka et al., 2016^*^). The relationship between anxiety disorder and Parkinson's disease is still controversial since 57% of patients with an anxiety disorder reported the onset of anxiety prior to the diagnosis while 43% reported the onset of anxiety after the diagnosis of Parkinson's disease (Dissanayaka et al., 2016^*^).

Clinical anxious symptoms were also observed in 13.7–38.5% (Lantéri-Minet et al., 2005^*^; Wei et al., 2016^*^; Yon et al., 2020^*^) of migraine patients (Sharma et al., 2013a^*^; Yon et al., 2020^*^), anxiety is particularly evident when headache becomes chronic (Zebenholzer et al., 2016^*^). However, some authors found more severe anxiety among chronic than episodic migraine patients (Yon et al., 2020^*^) while others found no differences (Robbins et al., 2011^*^).

The rate of anxious symptoms was 50.4% among Chronic Pain (CP) patients (i.e., lumbar, lower limb, pelvic/anal/perineal, peripheral neuropathic or muscle pain) (Ganasegeran et al., 2019^*^). The most frequent DSM-IV anxiety disorder was social anxiety (36.5%), followed by agoraphobia (8.5%), PD (7.3%), GAD (5.3%), specific phobia (3.5%) (Castro et al., 2009^*^). Among primary care patients with pain symptoms (i.e., muscle pain, headache, stomach pain), 40% received at least one DSM-IV anxiety disorder diagnosis (GAD 26.5%, social anxiety 18.6%, PD 17.7%) (Means-Christensen et al., 2008^*^). Among CP patients (i.e., fibromyalgia 31.5%, rheumatoid arthritis 21%, chronic pain not otherwise specified 16%), being female was significantly related to having more severe anxious symptoms (Costa et al., 2015^*^). Similarly, the rate of anxious symptoms in Chronic Pelvic Pain (CPP) women was 73 vs. 37% in subjects without CPP (Romão et al., 2009^*^) and anxiety was more severe among CPP women than among controls (Ter Kuile et al., 2010^*^). Finally, 40% of CCP cervical cancer survivors were considered clinically anxious (Vistad et al., 2011^*^).

Among postherpetic neuralgia patients, 19.4% had clinically significant anxiety (Schlereth et al., 2014^*^).

### Gastrointestinal Diseases

Anxiety was present in 28% of gastroenterological patients (Alosaimi et al., 2014^*^). Severe anxiety was observed in 27% of patients with chronic digestive system diseases. Patients with digestive system tumors had the highest rate of anxiety (55.19%), followed by those with liver cirrhosis (48.08%), chronic viral hepatitis (27.12%), or functional dyspepsia (25.55%) (Zhang et al., 2016^*^).

The incidence of severe anxiety was significantly higher in the non-erosive reflux disease group (16.5%) than in the reflux esophagitis one (10.4%) (Yang et al., 2015^*^). Among gastroesophageal reflux disease patients, a current clinical anxiety symptomology was found in 20.7% of cases (On et al., 2017^*^). Among gastroesophageal reflux disease patients, anxiety disorders were diagnosed in 30% of cases: 11.6% met the diagnostic criteria for specific phobias, 5.6% for lifetime social phobia, 5.6% for other phobic anxiety disorders, 3.8% for GAD, and 3.8% for agoraphobia with panic attacks (Grzesiak et al., 2014^*^).

Clinically relevant anxiety was observed among 21.1–41% of Inflammatory Bowel Disease (IBD) patients (Nahon et al., 2011^*^; Freitas et al., 2015^*^; Byrne et al., 2017^*^; Navabi et al., 2018^*^). The rate of severe anxious symptoms was 14.4% among Crohn's disease patients (Tomazoni and Benvegnú, 2018^*^). Anxiety was more represented in Crohn's disease (32.8%) than in ulcerative colitis patients (19.3%) (Freitas et al., 2015^*^). In addition, 33.7% of Crohn's disease and 32.4% of ulcerative colitis patients reported a potential anxiety disorder while 20.4% of Crohn's disease and 14.8% of ulcerative colitis patients had probable anxiety disorder (Häuser et al., 2011^*^).

The rate of anxious symptoms was 76.4% among Irritable Bowel Syndrome (IBS) patients (Baniasadi et al., 2017^*^) and 30% of these patients had clinically relevant anxiety (Thijssen et al., 2010^*^) with a rate of 24% among males and of 32% among females (Thijssen et al., 2010^*^). Anxiety was more severe among poor sleepers than good sleepers (Baniasadi et al., 2017^*^) while no difference was found when a depressive episode was in the past history of IBS patients (Tosic-Golubovic et al., 2010^*^). At least one anxiety disorder was diagnosed in 47% of IBS patients; the most common were: specific phobias (23.6%), GAD (10.4%), social phobia (10.4%), other phobic anxiety disorder (9.4%), agoraphobia (8.5%), anxiety disorder with panic attacks (3.8%) (Grzesiak et al., 2014^*^).

Anxiety was found more severe in patients with chronic hepatitis B virus (HBV) or chronic hepatitis C virus than among controls (Fotos et al., 2018^*^). Anxiety score was higher in the untreated chronic HBV patients than in the group treated with oral antiviral (Karlidag and Atmaca, 2019^*^). Among HBV patients, anxiety was negatively associated with health-related quality of life (Li et al., 2020a^*^). Anxious symptoms were found in 41–52% of patients with hepatitis C (Stewart et al., 2012^*^; Fortier et al., 2017^*^). Twenty-four percent had DSM-IV anxiety disorders, with a similar prevalence in both sexes. The commonest anxiety disorders were panic and phobic disorders (12.2%) and adjustment disorder with anxiety (6.7%) (Golden et al., 2005^*^).

Scoring for moderate anxiety was twice as frequent in autoimmune hepatitis (AIH) patients than in the general population (Schramm et al., 2014^*^).

### Genitourinary Diseases

Clinically relevant anxious symptoms were found in 26.6–71% of Chronic Kidney Disease patients (Lee et al., 2013^*^; Aggarwal et al., 2017^*^; Rebollo Rubio et al., 2017^*^; Mosleh et al., 2020^*^) who were also diagnosed with DSM-IV anxiety disorders (GAD 3.7%, PD 1.9%) (Kokoszka et al., 2016^*^). However, in one study patients on chronic hemodialysis had no more anxiety than those with a chronic illness or healthy subjects (Klarić et al., 2009^*^).

Between 22 and 45% of End-Stage Renal Disease (ESRD) patients presented clinically significant anxiety (Macaron et al., 2014^*^; Brito et al., 2019^*^; Schouten et al., 2019^*^) and among those on HD treatment, 21.1–32.3% had anxiety (Brito et al., 2019^*^). A total of 39.6–47.5% of chronic HD patients were found with clinically relevant anxiety (Bossola et al., 2010^*^; Semaan et al., 2018^*^), which included moderate (10.8%), severe (3.6%) (Semaan et al., 2018^*^), moderate or severe anxiety (48.7%) (Bossola et al., 2010^*^). Among maintenance hemodialysis (MHD) patients, 10–43% showed clinically relevant anxiety (Zhang et al., 2014^*^). Moderate anxiety (Czyzewski et al., 2018^*^) affected 20.3% of transplantation patients (Brito et al., 2019^*^). Overall, 16.5–45.7% of HD patients with ESRD met the criteria for one DSM-IV anxiety disorder (specific phobias 7.3–26.6%, PD with or without agoraphobia 2.7–21%, anxiety disorder NOS 7%, social anxiety 4.2–9.7%, GAD 1.4–18.4%) (Cukor et al., 2008^*^; Preljevic et al., 2012^*^; El Filali et al., 2017^*^).

### Endocrine Diseases

DSM-IV anxiety diagnoses were found in 42.5% of endocrine patients, GAD was the most frequent diagnosis (29%), followed by agoraphobia (8%) and PD (5%) (Sonino et al., 2004^*^).

The rate of subjects with clinically relevant anxiety was significantly higher in type 2 diabetes patients if compared with healthy controls (De Kort et al., 2012^*^). Diabetic patients showed a rate of anxious symptoms of 9–65.4% (Lewko et al., 2012^*^; Weaver and Madhu, 2015^*^; Sun et al., 2016^*^; Dong et al., 2020^*^; Woon et al., 2020^*^) while 19.5–48.6% of diabetes patients had mild anxiety, 9.5–26.3% moderate, 2.4–25% severe (Ganasegeran et al., 2014^*^; AlBekairy et al., 2017^*^; Khan et al., 2019^*^; Sharma et al., 2021^*^). Higher anxiety was statistically significant in females, older participants, individuals with longer duration of diabetes, those taking non-insulin treatment, and individuals with painful neuropathy, nephropathy, and foot ulcers (Khan et al., 2019^*^). Anxiety was negatively correlated with self-efficacy, but positively correlated with illness complications and depression (Wu et al., 2013^*^). Anxiety disorders were found in 49.7% of patients with diabetes (Sharma et al., 2021^*^). Among them, 39.5% received the diagnosis <2 years before the interview while 23.3% were diagnosed more than 2 years earlier (Weaver and Madhu, 2015^*^).

Seventy percent of patients with primary aldosteronism received a DSM-IV diagnosis of anxiety disorder (GAD 60%) (Sonino et al., 2006^*^). Higher prevalence of DSM-IV anxiety disorders was observed in patients with primary aldosteronism (52.2%) compared with patients with essential hypertension (EH) (17.4%). Moreover, GAD was more frequent in primary aldosteronism (30.4%) than in EH patients (8.7%) (Sonino et al., 2011^*^).

Twenty-five percent of women with hyperprolactinemia had severe anxiety compared to 19% in the control group (Reavley et al., 1997^*^).

Moderate and severe anxious symptoms were observed in 15 and 28% of obese patients (Ho et al., 2018), being significantly higher than in healthy subjects (Degirmenci et al., 2015^*^).

### Musculoskeletal System or Connective Tissue Diseases

Clinically relevant anxiety was found in 25.9–58% of Rheumatoid Arthritis (RA) (Barlow et al., 2002^*^; Dirik and Karanci 2010^*^; Ho et al., 2011^*^; Wan et al., 2015^*^; Zhang et al., 2017^*^; Larice et al., 2019^*^; Rahim and Cheng, 2018^*^; Beşirli et al., 2020^*^). Mild and moderate or severe anxious symptoms were found in 20.9–51.1% and in 26.4–35.9% RA patients, respectively (Cordingley et al., 2014^*^; Jamshidi et al., 2016^*^; Karahan et al., 2016^*^; Soósová et al., 2017^*^). Higher anxiety was associated with lower quality of life (Rogers et al., 2015^*^). The prevalence of ICD-9 anxiety disorders was found significantly higher among RA patients than controls (7.1 vs. 6.3%) (Watad et al., 2017^*^). ICD-10 anxiety disorders were diagnosed in 70% of RA patients (El-Miedany and El Rasheed, 2002^*^), being more common in males (100%) than in females (66.2%) (El-Miedany and El Rasheed, 2002^*^).

Patients with a diagnosis of Behçet's disease, Systemic Sclerosis (SSc), Still's disease, or Sjögren's syndrome had higher anxiety than patients with psoriasis or heathy subjects (Calikoglu et al., 2001^*^; Del Rosso et al., 2013^*^; Cui et al., 2018^*^; Chi et al., 2020^*^). In particular, 49.1% of SSc patients had moderate anxiety, 17.5% severe, and 6.1% needed psychiatrist's help due to anxiety (Faezi et al., 2017^*^). SSc patients also had social phobia (40%) (Leite et al., 2013^*^).

Among patients with Systemic Lupus Erythematosus (SLE), anxiety was found in 22.9–84.9% of cases (Kheirandish et al., 2014^*^; Zamora-Racaza et al., 2018^*^; Pinto et al., 2021^*^). Anxiety was moderate (Yilmaz-Oner et al., 2015^*^; Nowicka-Sauer et al., 2018^*^) and clinically relevant in 41% of cases (Azizoddin et al., 2017^*^), in 10.6% extreme (Yilmaz-Oner et al., 2015^*^). DSM-IV anxiety disorders were more common in women with SLE than in the general population. Specific phobia (24%), social phobia (16%), PD (8%), GAD (4%), and agoraphobia without panic disorder (1%) (Bachen et al., 2009^*^) were the most common diagnoses.

Anxiety was higher in Fibromyalgia (FM) than in healthy subjects (Pagano et al., 2004^*^; Alok et al., 2011^*^; Uçar et al., 2015^*^). A rate of 49.8% of FM patients had anxiety symptoms: 19%, 33%, 19% reporting mild, moderate and severe anxiety, respectively (Schaefer et al., 2011^*^). About 20% had a DSM-IV anxiety disorder, the most common was GAD (8.3%) and the incidence was significantly higher than among healthy controls (Kayhan et al., 2016^*^). Agoraphobia and GAD were more common in FM than in spondylarthritis or Sjogren's syndrome patients, while PD was more common in FM than in rheumatoid arthritis ones (Bucourt et al., 2019^*^).

Among patients with Osteoarthritis (OA), 74% had moderate anxiety (Montin et al., 2007^*^) and 84% had moderate anxiety before surgery (Montin et al., 2007^*^). Among lower limb OA patients, 31.5% had anxious symptoms (Axford et al., 2010^*^); among patients with chronic foot and ankle disease, 30% had anxiety (Nakagawa et al., 2017^*^). Among patients with hip pathology, 0.9% of the femoroacetabular impingement patients, 4.3% of the lateral trochanteric pain syndrome patients, 11.4% of the hip osteoarthrosis patients, and 43.8% of the avascular necrosis of the hip patients presented clinically significant anxiety (Hampton et al., 2019^*^); 37–65.7% of those suffering from rheumatic diseases reported clinical anxiety symptoms (Azad et al., 2008^*^; Anyfanti et al., 2014^*^), the majority (51.6 %) of whom exhibiting mild levels, 23.2% moderate anxiety, and 25.3% severe (Anyfanti et al., 2014^*^). The prevalence of anxiety was significantly higher in females than in males (40 vs. 21.8%) (Anyfanti et al., 2014^*^).

Among patients with benign fasciculation syndrome, 14.4% had clinically relevant anxiety (Filippakis et al., 2018^*^). The rate of anxious symptoms was 28.9% in chronic temporomandibular disorder patients (Bertoli and de Leeuw, 2016^*^).

DSM-III-R anxiety disorders were found in 17% of chronic low-back pain patients. Phobic disorders (9%), PD (3%), GAD (2%) were the most common (Polatin et al., 1993^*^). Among patients with chronic low back or neck pain caused by disc herniation, the prevalence of DSM-IV anxiety disorders was 35.8%. The most prevalent diagnoses were: GAD (12.8%), specific phobia (4.7%), social anxiety (0.7%), anxiety disorders not otherwise specified (16.2%) (Kayhan et al., 2015^*^).

### Dermatological Diseases

Patients with psoriasis showed to suffer from anxiety in 36–43% of cases (Richards et al., 2001^*^; Kwan et al., 2018^*^; Pollo et al., 2021^*^), particularly 5, 14, 12, and 6% reporting mild, moderate, severe, and extremely severe anxiety (Bulat et al., 2020^*^; Kwan et al., 2018^*^; Tian et al., 2018^*^).

Chronic urticaria patients had higher levels of anxiety than healthy subjects (Hashiro and Okumura, 1994^*^; Engin et al., 2007^*^; Yildirim et al., 2012^*^; Tat, 2019^*^) with 48% presenting clinically relevant anxiety. In addition, 25% of chronic urticaria patients had DSM-IV anxiety disorders (Staubach et al., 2005^*^).

The rate of anxious symptoms among patients with primary hyperhidrosis was 49.2%; the presence was higher in patients with axillary or craniofacial PH, compared with other areas (i.e. palmar, plantar) (Bragança et al., 2014^*^). The frequency in patients with hyperhidrosis (23.1%) was significantly higher if compared with patients without (7.5%) (Bahar et al., 2016^*^). Among patients with hereditary angioedema, 15–49.9% had anxious symptoms (Fouche et al., 2014^*^; Banerji et al., 2020^*^) which were severe in 7.6–25% of cases (Banerji et al., 2020^*^; Fouche et al., 2014^*^), mild in half of them, and moderate in about 25% (Fouche et al., 2014^*^).

Patients with chronic hand eczema, vitiligo, or atopic eczema presented more anxiety than healthy controls (Kouris et al., 2015^*^; Hamidizadeh et al., 2020^*^; Treudler et al., 2020^*^).

Overall, 58.3% of the patients with allergic conditions had abnormal anxiety whereas only 15.4% of those with non-allergic conditions had abnormal scores (Ponarovsky et al., 2011^*^). Among patients suffering from allergic skin disorders (i.e., allergic contact dermatitis, atopic dermatitis, urticaria), anxiety was higher if compared with patients with non-allergic conditions (i.e., pigmented nevi, irritant contact dermatitis, psoriasis, dyshydrotic eczema, acne, miscellaneous non-allergic conditions) (Ponarovsky et al., 2011^*^). Anxiety was found in 37.6% of patients with skin disease vs. 14.9% of healthy controls (Lukaviciute et al., 2020^*^); 28.6% of patients with androgenetic alopecia had anxiety (Bilaç et al., 2021^*^).

Overall, ICD-10 anxiety disorders were present in 18.4% of dermatology patients (Raikhy et al., 2017^*^), DSM-IV anxiety disorders in 16.7% of inpatients, of which 5.7% had GAD and 3.7% PD (Picardi et al., 2006^*^).

### Cancer

The rate of clinically relevant anxiety was 6.5–30% in cancer patients (Aass et al., 1997^*^; Shim and Hahm 2011^*^; Hong and Tian, 2014^*^; Cardoso et al., 2015^*^; Unseld et al., 2019^*^; Van den Brekel et al., 2020^*^; Naser et al., 2021^*^), mild anxiety was found in 29.8% of cases (Milligan et al., 2018^*^), moderate in 10.5% (Milligan et al., 2018^*^), severe in 1.8–15.5% (Milligan et al., 2018^*^; Truong et al., 2019^*^). Females scored significantly higher than males (Cardoso et al., 2015^*^; Unseld et al., 2019^*^), even at 12-month follow-up (Geue et al., 2019^*^). Among patients with advanced cancer that referred to palliative care services, severe anxious symptoms had a frequency of 25% (Smith et al., 2003^*^) and 44–52.1% had clinically relevant anxiety (Delgado-Guay et al., 2009^*^; Sewtz et al., 2021^*^). Of the 13.9% cancer patients receiving palliative care in Canada, 5.8% had GAD and 5.5% PD (DSM-IV) (Wilson et al., 2007^*^). Anxiety in cancer patients was found significantly higher than in their relatives (Karakoyun-Celik et al., 2010^*^).

The rate of anxious symptoms was 36.9% among oral cancer patients (Yuan et al., 2020^*^) while 30.2% of cancer patients with anxiety had a diagnosis of stomach cancer (Hong and Tian, 2014^*^). Among patients with gastrointestinal cancer, females had higher anxiety (Bektas and Demir, 2016^*^). Anxiety was found in digestive cancer patients also before surgery, before discharge, 6 months after discharge and in the advanced-phase (Matsushita et al., 2005^*^).

Eighteen percent of lung cancer patients presented anxiety (Williamson et al., 2020^*^). Anxious symptoms were found in 40–51% of those with advanced non-small cell lung cancer (Du-Quiton et al., 2010^*^; Yan et al., 2019^*^; Guo and Huang, 2021^*^) with no difference in rates between inpatients and outpatients (Du-Quiton et al., 2010^*^).

About 23% of bladder cancer patients had clinically relevant anxious symptoms (Mani et al., 2020^*^).

Between 19.5 and 78.3% of breast cancer patients reported moderate anxiety, while 15.4–19.2% had severe symptoms (Karakoyun-Celik et al., 2010^*^; Daştan and Buzlu, 2011^*^; Alacacioglu et al., 2013^*^). Among women with metastatic breast cancer, 17% showed mild anxiety and 28% moderate-severe anxiety (Park et al., 2018^*^). DSM-IV anxiety disorders were diagnosed in 9.9% of women with early stage breast cancer: specific phobia (4.3%), social anxiety (2.0%), GAD (1.7%), PD (1.3%), agoraphobia (0.6%) were described. Anxiety disorders were diagnosed in 6% of women with advanced breast cancer (specific phobia 2%, GAD 1.5%, agoraphobia 1.5%, social anxiety 1%) (Kissane et al., 2004^*^).

Among ovarian cancer patients, the highest level of anxiety was documented prior to surgery, anxiety generally decreased throughout the therapeutic process with a slight increase before the second cycle of chemotherapy (Mielcarek et al., 2016^*^).

The rate of anxiety among prostate cancer patients was 22% (Esser et al., 2020^*^), 10.6% of patients were identified as having high anxiety (Roth et al., 2006^*^), 12.5% had GAD and 4.6% met the diagnostic criteria for both GAD and a clinically significant anxiety specifically focused on their disease (Roth et al., 2006^*^).

A rate of 63% was found among differentiated thyroid cancer (DTC) patients hospitalized (Tagay et al., 2006^*^).

Fourteen percent of patients with neuroendocrine tumors (NETs) were classified as having clinically significant anxiety (Beesley et al., 2018^*^). Among patients with advanced gastroenteropancreatic neuroendocrine tumors, 30% had anxious symptoms (Lewis et al., 2018^*^).

Moderate levels of anxiety were found in 22.3% of hematological cancer patients (Abuelgasim et al., 2016^*^). Among chronic lymphocytic leukemia patients, 12% experienced moderate-severe anxiety (Robbertz et al., 2020^*^). In addition, 23–25% of Hodgkin Lymphoma patients had high anxiety vs. 13% of healthy controls (Daniëls et al., 2014^*^; Magyari et al., 2017^*^); 15% reported moderate anxiety and 10% severe anxiety (Magyari et al., 2017^*^). About 40% of lymphoma patients had anxiety (Wang et al., 2013b^*^). One third of multiple myeloma patients reported clinically relevant anxiety (Ramsenthaler et al., 2019^*^). Anxiety rate and score were higher among acute myeloid leukemia patients than healthy controls (Ding et al., 2019^*^).

About 27% of head and neck cancer had anxious symptoms and 10.8% had severe anxiety (Suzuki et al., 2016^*^).

Anxiety was more severe at stage I and II melanoma than at stage IV (Vojvodić and Dedic, 2020^*^) and it was found more severe among survivors of malignant melanoma than among healthy controls as well as in females if compared to males (Beutel et al., 2015^*^).

In renal cell carcinoma, lymphoma, plasma cell dyscrasia, or malignant melanoma patients, 48% reported anxiety (Stark et al., 2002^*^) and 18% fulfilled ICD-10 diagnostic criteria for anxiety disorders (phobia 13.5%, PD 9%, GAD 8.4%) (Stark et al., 2002^*^). For 31%, the current anxiety episode began more than 6 months before the diagnosis of cancer with 80% of episodes being over 2 years long (Stark et al., 2002^*^). For 38%, the episode began from 6 months before to 6 months after the diagnosis of cancer (Stark et al., 2002^*^). For 31% of patients, the episode began more than 6 months after the diagnosis of cancer (Stark et al., 2002^*^).

### Infections

From 13.8 to 49% of patients with AIDS had clinically significant anxiety (Sun et al., 2014^*^; Camara et al., 2020^*^; Park et al., 2021^*^): 31.9% mild (Betancur et al., 2017^*^), 8.4–27.7% moderate (Betancur et al., 2017^*^; Park et al., 2021^*^), 11.9–17% severe (Betancur et al., 2017^*^; Camara et al., 2020^*^; Park et al., 2021^*^). The rate was higher among females (Sun et al., 2014^*^; Mishkin et al., 2021^*^). HIV seropositives had clinically relevant anxiety in 21.6% of cases (Savard et al., 1998^*^). About 60% of HIV-infected pregnant women who continued pregnancy had anxiety (Qin et al., 2018^*^) and 34.6% of HIV migrants had severe anxiety (Been et al., 2019^*^). Among women with HIV, generalized anxiety symptoms were associated with poorer perceived general health and mental health functioning (Mishkin et al., 2021^*^).

Among HIV patients, 5% reported PD and 10% other anxiety disorders (Pence et al., 2006^*^). In addition, 30.8% of patients with HIV, tuberculosis or both was found to have at least one DSM-IV anxiety disorder (GAD 8.7%, PD 3.5%) (Van den Heuvel et al., 2013^*^).

Patients with Coronavirus disease 2019 (COVID-19) showed clinical anxiety symptoms in 20.9–38.5% of cases (Nie et al., 2020^*^; Zhang et al., 2020^*^; Zhou et al., 2021^*^) which was more severe than in healthy controls (Hao et al., 2020^*^). Anxiety showed to be severe in 2.6–97.3% of cases (Nie et al., 2020^*^; Moayed et al., 2021^*^), moderate-severe in 10.4–13% (Liu et al., 2020^*^; Yadav et al., 2021^*^), moderate in 12.8% (Nie et al., 2020^*^), mild in 20.5–54% (Liu et al., 2020^*^; Nie et al., 2020^*^; Yadav et al., 2021^*^). About 51% reported generalized anxiety (Zhang et al., 2021^*^) and 12.61% suffered from anxiety spectrum disorder (Zhang et al., 2021^*^).

### Miscellaneous

A rate of 28.7% of patients suffering from chronic illnesses had moderate anxiety, while the rate for severe anxiety was 61.3% (16^*^). In addition, 33.7% of hospitalized patients affected by different medical diseases had clinically relevant anxious symptoms (Gullich et al., 2013^*^). Moderate anxiety was found in 34.7% of hospitalized patients (Rani Chadalawada et al., 2016^*^). In patients with 79 different rare diseases, the rate of anxious symptoms was 23% and females presented significantly more severe symptomatology than males (Uhlenbusch et al., 2019^*^).

When patients recruited from different medical setting were evaluated, 12.6–23.5% were diagnosed with at least one anxiety disorder (4) (Guidi et al., 2011^*^), GAD was the most frequent (10.3%) (4). Similarly, DSM-IV anxiety disorders were found in 9–23.2% of inpatients with different chronic medical illness and the most common diagnoses were anxiety disorders not otherwise specified (9.5%) and GAD (7.6%) (Kayhan et al., 2013^*^; Al-Atram, 2018^*^). Overall, anxiety disorders were more prevalent than other psychiatric illnesses in patients with endocrine disorders (16.7%) and infections (11.3%) (Al-Atram, 2018^*^).

Patients with systemic hypertension and type 2 diabetes had mild anxiety in 32% of cases, moderate anxiety in 29%, and severe anxiety in 26% of cases (Teixeira et al., 2015^*^). Among patients with β-thalassemia major and intermedia, the rate of anxious symptoms was of 22.5% (Khoury et al., 2012^*^). About 30% of cystic fibrosis patients (Havermans et al., 2008^*^; Olveira et al., 2016^*^) presented severe anxious symptoms and 15% of those with neurofibromatosis type 1 described moderate-severe anxiety (Doser et al., 2020^*^).

Anxious symptoms were also present in 22.9–44% (Zhou et al., 2013^*^; Onwubiko et al., 2020^*^) of glaucoma patients although in another study the rates did not differ between glaucoma and non-glaucoma subjects (Rezapour et al., 2018^*^).

The overall frequency of anxious symptoms among otolaryngology patients was 22.9%, including moderate (14.9%) and severe (8%) anxiety (Al-Rawashdeh et al., 2018^*^). Among chronic tinnitus patients, the prevalence of DSM-IV anxiety disorders was significantly higher in females (58.3%) that in males (40.7%) (Malakouti et al., 2011^*^). Idiopathic ethmoidal nasal polyposis patients had high levels of anxiety in 23.3% of cases (Businco et al., 2004^*^) while orthotopic liver transplantation patients presented mild anxiety (27%), moderate anxiety (12%) or severe anxiety (7%) (Santos et al., 2010^*^). Among patients who received a diagnosis of benign breast lumps, the rate of anxiety was 40.2% (28.8% was classified as mild, 10.8% as moderate, 0.5% as severe) (Lou et al., 2015^*^). Anxious symptoms were found in 14.2% of patients with cubital tunnel syndrome (Jia et al., 2020^*^) while in lower limb amputation patients the frequency was 37% (Hawamdeh et al., 2008^*^).

Among inpatients with amenorrhea, the prevalence of GAD was 23.5% (Fava et al., 1984^*^). Patients with amenorrhea and hyperprolactinemia reported significantly more anxious symptoms than patients with amenorrhea (Fava et al., 1981^*^; Kellner et al., 1984^*^).

## Discussion

Moderate or severe anxiety occurs particularly among patients with chronic kidney disease, end-stage renal disease, hip pathology, systemic lupus erythematosus patients, hereditary angioedema and chronic urticarial, metastatic breast cancer, bladder cancer. Severe anxiety had the highest rates among patients with chronic illnesses, atrial fibrillation, coronary artery bypass graft, chronic thromboembolic pulmonary hypertension, pulmonary hypertension, chronic rhino sinusitis, asthma, migraine, multiple sclerosis, epilepsy, digestive system tumors, liver cirrhosis, irritable bowel syndrome, obesity, type 2 diabetes, hyperprolactinemia, COVID-19 infection.

The most common anxiety disorder was GAD, which was observed among patients with cardiovascular, respiratory, CNS, dermatologic diseases, and also in cancer, primary aldosteronism, amenorrhea, COVID-19 infection. Patients with cardiovascular, respiratory, or dermatology diseases also presented PD. Social anxiety was described for cardiovascular, respiratory, rheumatoid diseases. Specific phobias were relatively common in irritable bowel syndrome, gastroesophageal reflux, end-stage renal disease.

The present results should be considered as an overview of such clinical phenomenon which is in need of being further explored clarifying, among the others, temporal or causal relationships between anxiety and organic illnesses. In addition, we found studies referring to different levels of severity (i.e., mild/moderate/severe) of anxious symptoms that should be taken into account since the various levels of severity may impact the discomfort and the functioning of patients differently. In addition, such heterogeneity of severity, which was measured *via* different tools and in some cases also using different cut-offs for the same tool, suggest caution in interpreting the results and in using them to draw conclusions in comparability. Future studies increasing the body of evidence for each level of severity of anxiety in each medical disease are warranted to overcome this limitation. Also research aimed at disentangling between a physiological anxious reaction to the physical illness and a pathological, thus for instance maladaptive, response to the status of being medically ill are needed.

Anxiety represents a major challenge in medical settings, being highly represented either as a symptom or as a disorder (39). Nowadays, anxiety can be properly assessed in the medically ill *via* clinician- or self-reported measures (40, 41). This may provide information on the overall health condition, also according to a longitudinal view of development of disorders (42, 43), thus demarcating major prognostic and therapeutic differences among patients who otherwise might seem to be deceptively similar since they share the same diagnoses. It also allows to catch the possible interplay between mental and organic disease, for instance clarifying if there is a primary/secondary relationship (44, 45). It can help verifying whether the patient is at risk of developing depression, which often coexists with anxiety (46, 47) and worsen its prognosis. It allows to investigate other areas associated to anxiety. Among them, it allows to investigate illness behavior, the ways in which individuals experience, perceive, evaluate, and respond to their health status (48, 49). This is a transdiagnostic core characterization (50), with multiple expressions (51, 52), providing an explanatory model for clinical phenomena (8). Relevant information can be obtained also assessing mental pain (53, 54), which captures a feeling state characterized by emotional pain, emptiness, and internal perturbation (55), sometimes at the core of the suicidal process (56). Finally, a comprehensive assessment may also include evaluating specific positive features, i.e. psychological well-being (57, 58), which can be eventually empowered to cope with anxiety (59) and the organic disease (60, 61).

Recognition and proper assessment of anxiety represent the necessary steps for its appropriate management. Clinicians boast a large and effective armamentarium to treat anxiety, which include both pharmacological (e.g., benzodiazepines) (62, 63) and non-pharmacological (e.g., wellbeing therapy) interventions (59), they need to use it.

## Data Availability Statement

The original contributions presented in the study are included in the article/Supplementary Material, further inquiries can be directed to the corresponding author/s.

## Author Contributions

FC designed the systematic review. SR and GM screened the titles and abstracts and ascertained the validity of the eligible studies. SR extracted data from original papers and wrote the original draft. GM and FC reviewed all versions of the manuscript. All authors contributed to the article and approved the submitted version.

## Conflict of Interest

The authors declare that the research was conducted in the absence of any commercial or financial relationships that could be construed as a potential conflict of interest.

## Publisher's Note

All claims expressed in this article are solely those of the authors and do not necessarily represent those of their affiliated organizations, or those of the publisher, the editors and the reviewers. Any product that may be evaluated in this article, or claim that may be made by its manufacturer, is not guaranteed or endorsed by the publisher.

## Supplementary Material

References with ^*^ are listed in the reference list of the Supplementary Material.

## References

[1] Marks IM. Living With Fear: Understanding and Coping With Anxiety. New York, NY: McGraw-Hill (2001).

[2] Magni G Schifano F De Dominicis MG Belloni G. Psychological distress in geriatric and adult medical in-patients. Arch Gerontol Geriatr. (1988) 7:151–61. 10.1016/0167-4943(88)90027-1 3415395

[3] Kariuki-Nyuthe C Stein DJ. Anxiety and related disorders and physical illness. Key Issues Ment Health. (2014) 179:81–7. 10.1159/000365538

[4] Fava GA Porcelli P Rafanelli C Mangelli L Grandi S. The spectrum of anxiety disorders in the medically ill. J Clin Psychiatry. (2010) 71:910–4. 10.4088/jcp.10m06000blu 20584526

[5] Bengtson MB Aamodt G Vatn MH Harris JR. Co-occurrence of IBS and symptoms of anxiety or depression, among Norwegian twins, is influenced by both heredity and intrauterine growth. BMC Gastroenterol. (2015) 15:9. 10.1186/s12876-015-0237-y 25649866 PMC4321711

[6] Gerrits MMJG Vogelzangs N van Oppen P van Marwijk HWJ van der Horst H Penninx BWJH. Impact of pain on the course of depressive and anxiety disorders. Pain. (2012) 153:429–36. 10.1016/j.pain.2011.11.001 22154919

[7] Tzur Bitan D Krieger I Comaneshter D Cohen AD Feingold D. The association between the socioeconomic status and anxiety-depression comorbidity in patients with psoriasis: a nationwide population-based study. J Eur Acad Dermatol Venereol. (2019) 33:1555–61. 10.1111/jdv.15651 31054151

[8] Cosci F Guidi J. The role of illness behavior in the COVID-19 pandemic. Psychother Psychosom. (2021) 90:156–9. 10.1159/000513968 33517335 PMC7900455

[9] Nardi AE Cosci F. Expert opinion in anxiety disorder: Corona-phobia, the new face of anxiety. Personal Med Psychiatry. (2021) 25–26:100070. 10.1016/j.pmip.2021.100070

[10] Polikandrioti M Koutelekos I Vasilopoulos G Gerogianni G Gourni M Zyga S . Anxiety and depression in patients with permanent atrial fibrillation: prevalence and associated factors. Cardiol Res Pract. (2018) 2018:1–9. 10.1155/2018/7408129 29670767 PMC5836417

[11] Levenson JL. Anxiety in the medically ill. Prim Psychiatry. (2005) 12:24–6.

[12] Mago R Gomez J-P Gupta N Kunkel EJS. Anxiety in medically III patients. Curr Psychiatry Rep. (2006) 8:228–33. 10.1007/s11920-006-0028-9 19817074

[13] Katon W Lin EH Kroenke K. The association of depression and anxiety with medical symptom burden in patients with chronic medical illness. Gen Hosp Psychiatry. (2007) 29:147–55. 10.1016/j.genhosppsych.2006.11.005 17336664

[14] Arrieta Ó Angulo LP Núñez-Valencia C Dorantes-Gallareta Y Macedo EO Martínez-López D . Association of depression and anxiety on quality of life, treatment adherence, and prognosis in patients with advanced non-small cell lung cancer. Ann Surg Oncol. (2013) 20:1941–8. 10.1245/s10434-012-2793-5 23263699

[15] Xu W Collet JP Shapiro S Lin Y Yang T Platt RW . Independent effect of depression and anxiety on chronic obstructive pulmonary disease exacerbations and hospitalizations. Am J Respir Crit Care Med. (2008) 178:913–20. 10.1164/rccm.200804-619OC 18755925

[16] Fattouh N Hallit S Salameh P Choueiry G Kazour F Hallit R. Prevalence and factors affecting the level of depression, anxiety and stress in hospitalized patients with a chronic disease. Perspect Psychiatr Care. (2019) 55:592–9. 10.1111/ppc.12369 30825393

[17] Székely A Balog P Benkö E Breuer T Székely J Kertai MD . Anxiety predicts mortality and morbidity after coronary artery and valve surgery—a 4-year follow-up study. Psychosom Med. (2007) 69:625–31. 10.1097/PSY.0b013e31814b8c0f 17724254

[18] Tsuchihashi-Makaya M Kato N Chishaki A Takeshita A Tsutsui H. Anxiety and poor social support are independently associated with adverse outcomes in patients with mild heart failure. Circ J. (2009) 73:280–7. 10.1253/circj.cj-08-0625 19096191

[19] Dossa A Glickman ME Berlowitz D. Association between mental health conditions and rehospitalization, mortality, and functional outcomes in patients with stroke following inpatient rehabilitation. BMC Health Serv Res. (2011) 11:1–10. 10.1186/1472-6963-11-311 22085779 PMC3280187

[20] Celano CM Millstein RA Bedoya CA Healy BC Roest AM Huffman JC. Association between anxiety and mortality in patients with coronary artery disease: A meta-analysis. Am Heart J. (2015) 170:1105–15. 10.1016/j.ahj.2015.09.013 26678632 PMC4684590

[21] Chen CK Tsai YC Hsu HJ Wu IW Sun CY Chou CC . Depression and suicide risk in hemodialysis patients with chronic renal failure. Psychosomatics. (2010). 51:528–528.e6. 10.1016/s0033-3182(10)70747-7 21051686

[22] Gürhan N Beşer NG Polat Ü Koç M. Suicide risk and depression in individuals with chronic illness. Commun Ment Health J. (2019) 55:840–8. 10.1007/s10597-019-00388-7 30848413

[23] Bener A Verjee M Dafeeah EE Falah O Al-Juhaisi T Schlogl A . Psychological factors: anxiety, depression, and somatization symptoms in low back pain patients. J Pain Res. (2013) 6:95–101. 10.2147/jpr.s40740 23403693 PMC3569050

[24] Samaha HMS Elsaid AR Sabri Y. Depression, anxiety, distress and somatization in asthmatic patients. Egypt J Chest Dis Tuberc. (2015) 64:307–11. 10.1016/j.ejcdt.2015.02.010

[25] Alam ES Musselman DL Chyou D Shukri G Levine CG Sanghvi S . Somatization, depression, and anxiety disorders in a rhinology practice. Am J Rhinol Allergy. (2019) 33:470–7. 10.1177/1945892419841317 30947506

[26] Sherbourne CD. Prevalence of comorbid anxiety disorders in primary care outpatients. Arch Fam Med. (1996) 5:27–34. 10.1001/archfami.5.1.27 8542051

[27] Ball S Goddard A Shekhar A. Evaluating and treating anxiety disorders in medical settings. J Postgrad Med. (2002) 48:317–21. 12571395

[28] Spies G Asmal L Seedat S. Cognitive-behavioural interventions for mood and anxiety disorders in HIV: a systematic review. J Affect Disord. (2013) 150:171–80. 10.1016/j.jad.2013.04.018 23688915 PMC8811152

[29] Weisberg RB Beard C Moitra E Dyck I Keller MB. Adequacy of treatment received by primary care patients with anxiety disorders. Depress Anxiety. (2014) 31:443–50. 10.1002/da.22209 24190762 PMC4157338

[30] Shepardson RL Buchholz LJ Weisberg RB Funderburk JS. Psychological interventions for anxiety in adult primary care patients: a review and recommendations for future research. J Anxiety Disord. (2018) 54:71–86. 10.1016/j.janxdis.2017.12.004 29427898 PMC7909724

[31] Calleo J Stanley MA Greisinger A Wehmanen O Johnson M . Generalized anxiety disorder in older medical patients: diagnostic recognition, mental health management and service utilization. J Clin Psychol Med Settings. (2009) 16:178–85. 10.1007/s10880-008-9144-5 19152056 PMC2684857

[32] Huang C Huang L Wang Y Li X Ren L Gu X . 6-month consequences of COVID-19 in patients discharged from hospital: a cohort study. Lancet. (2021) 397:220–32. 10.1016/S0140-6736(20)32656-8 33428867 PMC7833295

[33] Hüsing P Löwe B Piontek K Shedden-Mora M. Somatoform disorder in primary care: the influence of co-morbidity with anxiety and depression on health care utilization. J Eval Clin Pract. (2018) 24:892–900. 10.1111/jep.12898 29498165

[34] Sinnema H Terluin B Volker D Wensing M van Balkom A. Factors contributing to the recognition of anxiety and depression in general practice. BMC Fam Pract. (2018) 19:99. 10.1186/s12875-018-0784-8 29935537 PMC6015659

[35] Zisselman MH Rovner BW Kelly KG Woods C. Benzodiazepine utilization in a university hospital. Am J Med Qual. (1994) 9:138–41. 10.1177/0885713x9400900306 7950486

[36] Munn Z Moola S Lisy K Riitano D Tufanaru C. Methodological guidance for systematic reviews of observational epidemiological studies reporting prevalence and cumulative incidence data. Int J Evid-Based Healthc. (2015) 13:147–53. 10.1097/XEB.0000000000000054 26317388

[37] Page MJ McKenzie JE Bossuyt PM Boutron I Hoffmann TC Mulrow CD . The PRISMA 2020 statement: an updated guideline for reporting systematic reviews. J Clin Epidemiol. (2021) 134:178–89. 10.1136/bmj.n71 33789819

[38] Spielberger CD Gorsuch RL Lushene R Vagg PR Jacobs GA. Manual for the State-Trait Anxiety Inventory. Palo Alto, CA: Consulting Psychologists Press (1983).

[39] Bech P. Mood and anxiety in the medically ill. Adv Psychosom Med. (2012) 32:118–32. 10.1159/000330012 22056902

[40] Kastrup M Bech P Rafaelsen OJ. Rating scales for states for anxiety, depression, mania and schizophrenia. A multiaxial approach. Acta Psychiatr Belg 1986 86(5). (1986) 575–81. 2881427

[41] Carrozzino D Patierno C Guidi J Berrocal Montiel C Cao J Charlson ME . Clinimetric criteria for patient-reported outcome measures. Psychother Psychosom. (2021) 90:222–32. 10.1159/000516599 34038901

[42] Cosci F Fava GA. Staging of mental disorders: systematic review. Psychother Psychosom. (2013) 82:20–34. 10.1159/000342243 23147126

[43] Bokma WA Batelaan NM Hoogendoorn AW Penninx BW van Balkom AJ. A clinical staging approach to improving diagnostics in anxiety disorders: Is it the way to go? Aust NZ J Psychiatry. (2020) 54:173–84. 10.1177/0004867419887804 31793794

[44] Cassem EH. Depression and anxiety secondary to medical illness. Psychiatr Clin North Am. (1990) 13:597–612. 2281008

[45] Cosci F Fava GA Sonino N. Mood and anxiety disorders as early manifestations of medical illness: a systematic review. Psychother Psychosom. (2015) 84:22–9. 10.1159/000367913 25547421

[46] Eysenck MW Fajkowska M. Anxiety and depression: toward overlapping and distinctive features. Cogn Emot. (2018) 32:1391–400. 10.1080/02699931.2017.1330255 28608767

[47] Cosci F Fava GA. When anxiety and depression coexist: the role of differential diagnosis using clinimetric criteria. Psychother Psychosom. (2021) 90:308–17. 10.1159/000517518 34344013

[48] Mystakidou K Tsilika E Parpa E Athanasouli P Galanos A Anna P . Illness-related hopelessness in advanced cancer: influence of anxiety, depression, and preparatory grief. Arch Psychiatr Nurs. (2009) 23:138–47. 10.1016/j.apnu.2008.04.008 19327556

[49] Fava GA Cosci F Sonino N. Current psychosomatic practice. Psychother Psychosom. (2017) 86:13–30. 10.1159/000448856 27884006

[50] Cosci F. Clinimetric perspectives in clinical psychology and psychiatry. Psychother Psychosom. (2021) 90:217–21. 10.1159/000517028 34052804

[51] Pilowsky I. Abnormal Illness Behaviour. Chichester, England: Wiley (1997).

[52] Fava GA. Well-being therapy: current indications and emerging perspectives. Psychother Psychosom. (2016) 85:136–45. 10.1159/000444114 27043240

[53] Fava GA Tomba E Brakemeier E-L Carrozzino D Cosci F Eöry A . Mental pain as a transdiagnostic patient-reported outcome measure. Psychother Psychosom. (2019) 88:341–9. 10.1159/000504024 31665739

[54] Sensky T. Mental pain and suffering: the “universal currencies” of the illness experience? Psychother Psychosom. (2020) 89:337–44. 10.1159/000509587 32781446

[55] Cosci F Mansueto G Benemei S Chiarugi A De Cesaris F Sensky T. Mental pain as a global person-centered outcome measure. CNS Spectr. (2021) 2021:1–7. 10.1017/s1092852921000699 34311805

[56] Orbach I. Mental pain and suicide. Isr J Psychiatry Relat Sci. (2003) 40:191–201. 14619678

[57] Cosci F Fava GA. The clinical inadequacy of the DSM-5 classification of somatic symptom and related disorders: an alternative trans-diagnostic model. CNS Spectr. (2016) 21:310–7. 10.1017/s1092852915000760 26707822

[58] Ryff CD. Psychological well-being revisited: advances in the science and practice of eudaimonia. Psychother Psychosom. (2014) 83:10–28. 10.1159/000353263 24281296 PMC4241300

[59] Cosci F. Well-being therapy in anxiety disorders. In: Y. K. Kim, editor. Anxiety Disorders. Advances in Experimental Medicine and Biology (Singapore: Springer), 465–85. 10.1007/978-981-32-9705-0_24 32002942

[60] Bassi M Falautano M Cilia S Goretti B Grobberio M Pattini M . Illness perception and well-being among persons with multiple sclerosis and their caregivers. J Clin Psychol Med Settings. (2016) 23:33–52. 10.1007/s10880-015-9425-8 26216661

[61] Mansueto G Cosci F. Well-being therapy for depressive symptoms in chronic migraine: a case report. Clin Case Stud. (2021) 20:296–309. 10.1177/1534650121989812

[62] Balon R Chouinard G Cosci F Dubovsky SL Fava GA Freire RC . International task force on benzodiazepines. Psychother Psychosom. (2018) 87:193–4. 10.1159/000489538 29788029

[63] Silberman E Balon R Starcevic V Shader R Cosci F Fava GA . Benzodiazepines: it's time to return to the evidence. Br J Psychiatry. (2021) 218:125–7. 10.1192/bjp.2020.164 33040746

