# Supplementary material for: Anxiety in the Medically Ill: A Systematic Review of the Literature
Source: Front Psychiatry. 2022 Jun 3;13:873126. doi: 10.3389/fpsyt.2022.873126 (PMC9203680; doi:10.3389/fpsyt.2022.873126)
Supplement: Supplementary file 1 [file Table_1.docx]

**Table S1.**

*Overview of Studies on Anxiety in the Medically Ill Patients. Studies were presented based on a chronological order*

| Reference | Country/City | Study Design | Diagnosis | Sample size | Instrument to assess anxiety | Psychopathological manifesttaions co-occurring with anxiety | JBI Checklist-total score |
| --- | --- | --- | --- | --- | --- | --- | --- |
| Fava et al., 1981 | Italy | Cross-sectional and case-control study | Secondary amenorrhea | Inpatients with amenorrhea and hyperprolactinemia, n=10  inpatients with amenorrhea,  n=10  healthy controls,  n=10 | SQ | **SQ**  Inpatients with amenorrhea and hyperprolactinemia (SQ mean ± SD: 13±4) had more severe depressive symptoms than inpatients with only amenorrhea (SQ mean ± SD: 7.6±2.4, p<0.001) and healthy controls (SQ mean ± SD: 3±2.5, p<0.001) | **7** |
| Fava et al., 1984 | Italy | Cross-sectional and observational study | Secondary amenorrhea | Inpatients with secondary amenorrhea,  n=18 | Clinical Interview for DSM-III | **Clinical Interview for Depression**  **(DSM-III)**  28% of inpatients with secondary amenorrhea had a major depressive disorder | **6** |
| Kellner et al., 1984 | New Mexico | Cross-sectional and case-control study | Hyperprolactinemia | Hyperprolactinemic women,  n=14  patients with psychiatric disease,  n=25  family practice patients,  n=29  non-patient employees,  n=26 | SQ | **SQ**  Hyperprolactinemic women and psychiatric patients did not differ for the severity of depressive symptoms (SQ mean ± SD: 9.9±3.7 vs 11 ± 5.4, p > 0.05)  Hyperprolactinemic women had more severe depressive symptoms than family practice patients (SQ mean ± SD 4.0 ± 3.8, p < 0.05) and non-patient employees (SQ mean ± SD 2.5 ± 2.6, p < 0.01) | **7** |
| Reference | **Country/City** | **Study Design** | **Diagnosis** | **Sample size** | **Instrument to assess anxiety** | **Psychopathological manifesttaions co-occurring with anxiety** | **JBI Checklist-total score** |
| Beitman, et al., 1989 | Columbia | Cross-sectional and observational study | Chest pain and angiographically normal coronary arteries | Outpatients with  chest pain and angiographically normal coronary arteries,  n=94 | SCID-UP-R for DSM-III-R  SAS | **SCID-UP-R for DSM-III-R**  12% of outpatients with chest pain and angiographically normal coronary arteries had a major depressive disorder  3% of outpatients with chest pain and angiographically normal coronary arteries had an alcohol abuse | **7** |
| Karajgi, et al., 1990 | USA | Cross-sectional and observational study | Chronic obstructive pulmonary disease | Outpatients with chronic obstructive pulmonary disease, n=50 | SCID for DSM-III-R | **SCID for DSM-III-R**  18% of outpatients with chronic obstructive pulmonary disease had a mood disorder  10% of outpatients with chronic obstructive pulmonary disease had a past episode of major depression  6% of outpatients with chronic obstructive pulmonary disease had a current episode of major depressive disorder  2% of outpatients with chronic obstructive pulmonary disease had a current episode of dysthymia  8% of outpatients with chronic obstructive pulmonary disease had alcohol abuse | **6** |
| Menza et al., 1993 | USA | Cross-sectional and case-control study | Parkinson' s disease | Outpatients with Parkinson' s disease, n=42  Subjects with chronic, debilitating osteoarthritis,  n=21 | SAS  Clinical Interview for DSM-III-R | **SDS**  43% of outpatients with Parkinson' s disease had a depressive disorder diagnosis  14% of outpatients with Parkinson' s disease had depressive symptoms but no formal DSM-III-R diagnosis  14% of controls had a depressive disorder diagnosis  14% of controls had depressive symptoms but no formal diagnosis.  The difference in prevalence of depressive disorder diagnoses between Parkinson' s disease outpatients and controls was significant (p < 0.05) | **7** |
| Reference | **Country/City** | **Study Design** | **Diagnosis** | **Sample size** | **Instrument to assess anxiety** | **Psychopathological manifesttaions co-occurring with anxiety** | **JBI Checklist-total score** |
| Polatin et al., 1993 | Texas | Cross-sectional and observational study | Chronic low-back pain | Outpatients with chronic low-back pain, n=200 | SCID for DSM-III-R | **SCID for DSM-III-R (lifetime prevalence)**  97% of outpatients with chronic low-back pain had a lifetime history of somatoform disorder  64% of outpatients with chronic low-back pain had a lifetime history of major depressive disorder  36% of outpatients with chronic low-back pain had a lifetime history of substance abuse disorder  3% of outpatients with chronic low-back pain had a lifetime history of psychotic disorder  **SCID for DSM-III-R (current prevalence)**  45% of outpatients with chronic low-back pain patients had a major depressive disorder  19% of outpatients with chronic low-back pain patients had a substance abuse disorder  33% of outpatients with chronic low-back pain patients had a paranoid personality disorder  15% of outpatients with chronic low-back pain patients had a borderline personality disorder  14% of outpatients with chronic low-back pain patients had a avoidant personality disorder  12% of outpatients with chronic low-back pain patients had a passive-aggressive personality disorder | **7** |
| Reference | **Country/City** | **Study Design** | **Diagnosis** | **Sample size** | **Instrument to assess anxiety** | **Psychopathological manifesttaions co-occurring with anxiety** | **JBI Checklist-total score** |
| Hashiro et al., 1994 | Japan | Cross-sectional and case-control study | Chronic urticarial | Outpatients with chronic urticarial, n=30  healthy subjects,  n=39 | MAS, Japanese version | **SDS**  Outpatients with chronic urticarial had more depressive symptoms than healthy subjects (43.4% vs 12.8%, p < 0.01) (SDS score ≥ 40) | **7** |
| Noy et al., 1995 | Israel | Cross-sectional and observational study | Multiple sclerosis | Outpatients with multiple sclerosis, n=20 | HAM-A | **HAM-D**  In the whole sample HAM-D mean score was 13.1 ± 1.6  30% of outpatients with multiple sclerosis had mild depressive symptoms (HAM-D score 13-16)  20% of outpatients with multiple sclerosis had moderate to severe depressive symptoms (HAM-D score ≥ 17) | **7** |
| Aass et al., 1997 | Norway | Cross-sectional and observational study | Cancer | Outpatients/ inpatients with cancer, n=716 | HADS-A | **HADS-D**  9% of patients with cancer had clinically relevant depressive symptoms (HADS-D scores ≥ 11)  11% of patients with cancer had borderline depression (HADS-D scores 8-10) | **8** |
| Reference | **Country/City** | **Study Design** | **Diagnosis** | **Sample size** | **Instrument to assess anxiety** | **Psychopathological manifesttaions co-occurring with anxiety** | **JBI Checklist-total score** |
| Reavley et al., 1997 | UK | Cross-sectional and case-control study | Hyperprolactinemia | Outpatients with hyperprolactinemia, n=66  female outpatients with no functioning pituitary tumours or treated acromegaly, n=26 | HADS-A | **HADS-D**  Outpatients with hyperprolactinemia and controls had similar rates of depressive symptoms (25% vs 27%) (HADS-D score > 8) | **6** |
| Kohen et al., 1998 | UK | Cross-sectional and observational study | Diabetes | Outpatients with diabetes, n=100 | HADS-A | **HADS-D**  12% of outpatients with diabetes had borderline depression  (HADS-D score 8-10)  17% of outpatients with diabetes had possible depressive disorder  (HADS-D score >10) | **7** |
| Savard et al., 1998 | Quebec City | Cross-sectional and observational study | Human immunodeficiency virus (HIV) seropositive | Outpatients with HIV, n=162 | HADS-A | **HADS-D**  19.1% of outpatients with HIV were potential cases of depressive disorder (HADS-D score ≥ 8)  6.8% of HIV subjects were definite cases of depressive disorder (HADS-D score ≥ 10) | **7** |
| Sutton et al., 1999 | UK | Cross-sectional and observational study | Chronic obstructive pulmonary disease | Outpatients with chronic obstructive pulmonary disease, n=37 | HADS-A |  | **8** |
| Vamos et al., 1999 | New Zealand | Cross-sectional and observational study | Chronic asthma | Outpatients with chronic asthmatic, n=80 | HADS-A | **HADS-D**  10.3% of outpatients with chronic asthmatic had possible depression (HADS score ≥ 8)  1.5% of outpatients with chronic asthmatic had a definite depression (HADS score ≥10) | **7** |
| Reference | **Country/City** | **Study Design** | **Diagnosis** | **Sample size** | **Instrument to assess anxiety** | **Psychopathological manifesttaions co-occurring with anxiety** | **JBI Checklist-total score** |
| Calikoglu et al., 2001 | Turkey | Cross-sectional and case-control study | Behçet’s disease | Outpatients with Behçet’s disease,  n=23  Outpatients with chronic plaque-type psoriasis,  n=17 | BAI | **BDI**  Outpatients with Behçet’s disease had more severe depressive symptoms than outpatients with chronic plaque-type psoriasis (BDI mean ± SD: 11.69 ± 6.93 vs. 9.11 ± 7.34, p < 0.05) | **6** |
| Fruewald et al., 2001 | Vienna | Cross-sectional and observational study | Multiple sclerosis | Outpatients with multiple sclerosis, n=60 | SAS | **SDS**  Outpatients with multiple sclerosis had a SDS mean score (39.4 ± 8.5), distinctly over the average score of the general population (≤ 33) | **7** |
| Richards et al., 2001 | UK | Cross-sectional and observational study | Psoriasis | Outpatients with psoriasis,  n=115 | HADS-A | **HADS-D**  10% of outpatients with psoriasis had depressive symptoms (HADS-D score ≥ 11) | **7** |
| Barlow et al., 2002 | UK | Longitudinal and observational study | Rheumatoid arthritis | Outpatients with rheumatoid arthritis, n=60 | HADS-A | **HADS-D**  35% of outpatients with rheumatoid arthritis had depressive symptoms (HADS-D score ≥ 8) | **6** |
| El-Miedany et al., 2002 | Egypt | Cross-sectional and observational study | Rheumatoid arthritis | Outpatients with rheumatoid arthritis, n=80 | Research Diagnostic Criteria for the ICD-10 | **ICD-10**  66.2% of outpatients with rheumatoid arthritis had a major depressive disorder.  Depression was more common in males (89%) than in females (63%) | **7** |
| Reference | **Country/City** | **Study Design** | **Diagnosis** | **Sample size** | **Instrument to assess anxiety** | **Psychopathological manifesttaions co-occurring with anxiety** | **JBI Checklist-total score** |
| Marinus et al., 2002 | Maastricht | Cross-sectional and observational study | Parkinson’s disease | Outpatients with Parkinson’s disease, n=177 | HADS-A | **HADS-D**  21.5% of outpatients with Parkinson’s disease were possible depressed (HADS-D score 8-10)  16.9% of outpatients with Parkinson’s disease were probably depressed (HADS-D score ≥ 11) | **7** |
| Nascimento et al., 2002 | Rio de Janeiro | Cross-sectional and observational study | Asthma | Outpatients with asthma,  n=86 | M.I.N.I. for DSM-IV | **M.I.N.I.**  33.7% of outpatients with asthma had a major depressive episode  37.2% of outpatients with asthma had more than one current psychiatric diagnosis. In the panic agoraphobic spectrum patients (n=35), 27.9% had another psychiatric diagnosis, 20.9% had a major depressive episode | **7** |
| Stark et al., 2002 | UK | Cross-sectional and observational study | Cancer | Outpatients with cancer,  n=178 | HADS-A  SCAN for ICD-10 | **SCAN**  15% of outpatients with cancer had a major depressive disorder | **7** |
| Smith, 2003 | Manchester | Cross-sectional and observational study | Advanced cancer | Inpatients with advanced cancer underwent palliative care,  n=68 | HADS-A | **HADS-D**  22% of inpatients with advanced cancer had depressive symptoms  (HADS-D score ≥ 11) | **7** |
| Strik et al., 2003 | The Netherlands | Longitudinal and observational study | Acute myocardial infarction | Inpatients/ outpatients with acute myocardial infarction (male),  n=318 | SCL-90 | **SCL-90**  47.1% of patients with acute myocardial infarction scored above the cut-off value of depression subscale.  62.1% of acute myocardial infarction patients scored above the cut-off value of hostility subscale | **7** |
| Reference | **Country/City** | **Study Design** | **Diagnosis** | **Sample size** | **Instrument to assess anxiety** | **Psychopathological manifesttaions co-occurring with anxiety** | **JBI Checklist-total score** |
| Bankier et al., 2004 | Massachusetts | Cross-sectional and observational study | Coronary heart disease | Outpatients with coronary heart disease,  n=100 | SCID for DSM-IV | **SCID**  29% of outpatients with coronary heart disease had a past major depressive episode in full remission  15% of outpatients with coronary heart disease had a dysthymic disorder  31% of outpatients with coronary heart disease had a recurrent major depressive episode with current major depressive episode  19% of outpatients with coronary heart disease had an alcohol abuse  6% of coronary heart disease outpatients had an alcohol dependency  29% of outpatients with coronary heart disease had a post-traumatic stress disorder  10% of outpatients with coronary heart disease had a binge eating disorder  13% of outpatients with coronary heart disease had a primary insomnia  1% of outpatients with coronary heart disease had a schizoaffective disorder  1% of outpatients with coronary heart disease had an obsessive–compulsive disorder | **7** |
| Reference | **Country/City** | **Study Design** | **Diagnosis** | **Sample size** | **Instrument to assess anxiety** | **Psychopathological manifesttaions co-occurring with anxiety** | **JBI Checklist-total score** |
| Businco et al., 2004 | Italy | Cross-sectional and observational study | Nasal polyposis | Outpatients with nasal polyposis,  n=30 | STAI | **SDS**  16.6% of outpatients with nasal polyposis had depressive symptoms (SDS score ≥ 49) | **7** |
| Kissane et al., 2004 | Australia | Cross-sectional and observational study | Breast cancer | Outpatients with early stage breast cancer, n=303  Outpatients with advanced breast cancer,  n=200 | MILP for DSM-IV | **MILP for DSM-IV**  Major depression, dysthymia, adjustment disorder, were diagnosed in 37% of women with early stage breast cancer and in 31% of the women with advanced breast cancer  Substance abuse disorders were diagnosed in 28% of women with early stage disease breast cancer and in 22% of the women with advanced breast cancer | **8** |
| Löwe et al., 2004 | Germany | Cross-sectional and case-control study | Pulmonary hypertension | Outpatients with pulmonary hypertension,  n=164  Outpatients with Inflammatory rheumatic diseases, n=164  Primary care outpatients,  n= 164 | PHQ, German version for DSM-IV-TR | **PHQ**  35.4% of the outpatients with pulmonary hypertension had one or more mental disorders  27.4% of the outpatients with inflammatory rheumatic diseases had one or more mental disorders  24.4% of the primary care outpatients had one or more mental disorders  15.9% of outpatients with pulmonary hypertension had a major depressive disorder | **8** |
| Pagano et al., 2004 | San Paulo | Cross-sectional and case-control study | Fibromyalgia | Outpatients with fibromyalgia, n=40  healthy subjects, n=40 | STAI |  | **7** |
| Reference | **Country/City** | **Study Design** | **Diagnosis** | **Sample size** | **Instrument to assess anxiety** | **Psychopathological manifesttaions co-occurring with anxiety** | **JBI Checklist-total score** |
| Shafazand et al., 2004 | Stanford | Cross-sectional and observational study | Pulmonary arterial hypertension | Outpatients with pulmonary arterial hypertension,  n=53 | HADS-A | **HADS-D**  7.5% of outpatients with pulmonary arterial hypertension had moderate to severe depression (HADS-D score ≥ 11) | **7** |
| Sonino et al., 2004 | Italy | Cross-sectional and observational study | Endocrine disease | Outpatients endocrine disease,  n=146 | SCID for DSM-IV | **SCID for DSM-IV**  26% of endocrine disease outpatients had a major depressive disorder  7% of endocrine disease outpatients had adjustment disorder | **7** |
| Galeazzi et al., 2005 | Italy | Cross-sectional and case-control study | Multiple sclerosis | Outpatients with multiple sclerosis, n=50  healthy subjects,  n=50 | SCID-I for  DSM-IV  STAI | **BDI**  Outpatients with multiple sclerosis had more severe depressive symptoms than healthy subjects (BDI mean ± SD: 5.90 ± 5.80 vs 3.16 ± 3.64, p < 0.05)  **SCID for DSM-IV**  Multiple sclerosis outpatients, compared to healthy subjects, had higher rates of major depressive disorder (46% vs 18%, OR 3.17, 95%CI 1.56 - 9.65), major depressive episode (12% and 2%, OR 6.68, 95%CI 0.77 - 57.70), dysthymic disorder (10% vs 4%, OR 2.67, CI95% 0.49 - 24.44), bipolar disorder (6% vs 2%, OR: 3.13, 95%CI: 0.31 - 31.14), obsessive-compulsive disorder (10% vs 4%, OR 2.67, CI95% 0.49 - 14.44), alcohol use disorder (6% vs 0%) | **7** |
| Golden et al., 2005 | Ireland | Cross-sectional and case-control study | Hepatitis C | Outpatients with hepatitis C,  n=90 | SCID for DSM-IV | **SCID for DSM-IV (life time prevalence)**  36% of outpatients with hepatitis C had a lifetime history of depressive disorder  26.7% of outpatients with hepatitis C had a lifetime history of alcohol misuse  18.9% of outpatients with hepatitis C had had a lifetime history of opiate misuse  **SCID for DSM-IV (current prevalence)**  27.8% of outpatients with hepatitis C had a major depressive disorder (major depressive disorder was more prevalent in women 44% than in men 22%)  11% of outpatients with hepatitis C had an adjustment disorder with depressed mood (with or without anxiety)  9% of outpatients with hepatitis C had both depressive and anxiety disorders  1.1% of outpatients with hepatitis C had alcohol misuse  25.6% of outpatients with hepatitis C had opiate misuse | **7** |
| Reference | **Country/City** | **Study Design** | **Diagnosis** | **Sample size** | **Instrument to assess anxiety** | **Psychopathological manifesttaions co-occurring with anxiety** | **JBI Checklist-total score** |
| Haworth et al., 2005 | UK | Cross-sectional and observational study | Chronic heart failure | Outpatients with chronic heart failure, n=98 | SCID-I for  DSM-IV | **SCID-I for DSM-IV**  29% of outpatients with chronic heart failure had depressive disorders  14% of outpatients with chronic heart failure had a major depressive disorder  3% of outpatients with chronic heart failure had a dysthymic disorder  6% of outpatients with chronic heart failure had depressive disorder and panic disorder  9% of outpatients with chronic heart failure had depressive disorder and generalized anxiety disorder | **7** |
| Reference | **Country/City** | **Study Design** | **Diagnosis** | **Sample size** | **Instrument to assess anxiety** | **Psychopathological manifesttaions co-occurring with anxiety** | **JBI Checklist-total score** |
| Lantéri-Minet et al., 2005 | France | Cross-sectional and observational study | Migraine | Outpatients with migraine,  n=1957 | HADS-A | **HADS-D**  3.5% of outpatients with migraine had depressive symptoms (HASD-D score ≥ 8)  19.1% of outpatients with migraine had anxiety and depressive symptoms | **9** |
| Matsushita et al., 2005 | Japan | Longitudinal and case-control study | Digestive cancer (i.e., stomach, colon, rectum, oesophagus, pancreas cancers) | Inpatients with digestive cancer on advanced-phase or  on early phase,  n=85  patients hospitalized for surgical treatment of digestive disorders other than malignancy,  n=26 | HADS-A | **HADS-D**  Across three examination days (i.e., before surgery, before discharge, 6 months after discharge) inpatients with digestive cancer on advanced-phase had higher HADS-D scores (HADS-D mean ± SD: 3 ± 4; 4.5 ± 4.3; 3.1 ± 3.2) than inpatients with digestive cancer on early phase (HADS-D mean ± SD: 0.9 ± 1.5; 2.1 ± 3.1; 2.5 ± 3.3) and control group (HADS-D mean ± SD: 0.5 ± 1.2; 1.8 ± 3.7; 2 ± 2.9)  (p < 0.05) | **7** |
| Ottolini 2005 et al., | Italy | Cross-sectional and observational study | Myocardial infarction | Inpatients with myocardial infarction, n=92 | SCID for DSM-IV, Italian short version | **SCID for DSM-IV**  17% of inpatients with myocardial infarction had a major depressive e disorder | **7** |
| De Souza et al., 2006 | Brazil | Cross-sectional and case-control study | Epilepsy | Outpatients with epilepsy,  n=60  healthy subjects,  n=60 | STAI | **BDI**  Among outpatients with epilepsy, 40.63% of women and 21.43% of men had depression  Among healthy subjects, 7.50% of women and 20.00% of men had depressive symptoms | **7** |
| Reference | **Country/City** | **Study Design** | **Diagnosis** | **Sample size** | **Instrument to assess anxiety** | **Psychopathological manifesttaions co-occurring with anxiety** | **JBI Checklist-total score** |
| Huffman et al., 2006 | Boston | Cross-sectional and observational study | Acute myocardial infarction | Inpatients with acute myocardial infarction, n=74 | BAI | **BDI**  28% of inpatients with acute myocardial infarction had depressive symptoms (BDI score≥ 10)  **SCID I for DSM-IIV**  12% of inpatients with acute myocardial infarction inpatients had a major depressive disorder | **7** |
| Lavoie et al., 2006 | Montréal | Cross-sectional and observational study | Asthma | Outpatients with asthma,  n=504 | PRIME-MD for DSM-IV  ASI | **PRIME-MD for DSM-IV**  20% of outpatients with asthma had one or more depressive disorders  14% of outpatients with asthma had a major a depressive disorder  5% of outpatients with asthma had a minor depressive disorder  4% of outpatients with asthma had had a dysthymia  11% of asthmatic outpatients had depressive and anxiety disorders | **7** |
| Pence et al., 2006 | USA | Cross-sectional and observational study | HIV | Outpatients with HIV, n=1125 | SCID for DSM-IV | **SCID** **for DSM-IV**  16% of outpatients with HIV had a major depressive disorder  10% of outpatients with HIV outpatients had a depressive disorder not otherwise specified  6% of outpatients with HIV outpatients had a post-traumatic stress disorder  11% of outpatients with HIV outpatients had alcohol abuse or dependence  13.5% of outpatients with HIV had substance abuse or dependence | **8** |
| Reference | **Country/City** | **Study Design** | **Diagnosis** | **Sample size** | **Instrument to assess anxiety** | **Psychopathological manifesttaions co-occurring with anxiety** | **JBI Checklist-total score** |
| Picardi et al., 2006 | Italy | Cross-sectional and observational study | Skin diseases | Inpatients with skin diseases,  n=539 | SCID for DSM-IV | **SCID for DSM-IV**  20.2% of inpatients with skin diseases had a mood disorder  7% of inpatients with skin diseases had an adjustment disorder  6.9% of inpatients with skin had a somatoform disorder | **8** |
| Roth et al., 2006 | New York | Cross-sectional and observational study | Prostate cancer | Outpatients with prostate cancer, n=367 | MAX–PC  GAD-7 |  | **8** |
| Sonino et al., 2006 | Italy | Cross-sectional and observational study | Primary aldosteronism | Outpatients with primary aldosteronism,  n=10 | SCID for DSM-IV | **SCID for DSM-IV**  1% of outpatients with primary aldosteronism had a major depressive disorder  1% of outpatients with primary aldosteronism had an obsessive-compulsive disorder | **7** |
| Staubach et al., 2006 | Germany | Cross-sectional and case-control study | Chronic urticarial | Outpatients with chronic urticarial, n=100  Healthy subjects,  n=96 | M.I.N.I. DIPS for DSM-IV | **M.I.N.I.DIPS for DSM-IV**  11% of outpatients with chronic urticarial had a major depressive disorder;  11% of outpatients with had a somatoform disorder | **7** |
| Tagay et al., 2006 | Germany | Cross-sectional and observational study | Diﬀerentiated thyroid cancer | Inpatients with diﬀerentiated thyroid cancer,  n=136 | HADS-A | **HADS-D**  17% of inpatients with diﬀerentiated thyroid cancer had depressive symptoms: 9% patients were identiﬁed as borderline depression cases (HADS-D 8–10 score) and 8% as depression cases (HADS-D 11–21 score) | **7** |
| Reference | **Country/City** | **Study Design** | **Diagnosis** | **Sample size** | **Instrument to assess anxiety** | **Psychopathological manifesttaions co-occurring with anxiety** | **JBI Checklist-total score** |
| Valença et al., 2006 | Rio de Janerio | Cross-sectional and observational study | Asthma | Outpatients with asthma,  n=62 | M.I.N.I. for DSM-IV | **M.I.N.I. for DSM-IV**  24.2% of outpatients with asthma had a major depressive disorder  3.2% of outpatients with asthma had an obsessive compulsive disorder  1.6% of outpatients with asthma had a psychotic disorder  Among asthmatic outpatients with a major depressive disorder, 66.6% had a comorbid anxiety disorder | **8** |
| White et al., 2006 | Utah | Cross-sectional and observational study | Pulmonary arterial hypertension | Outpatients with pulmonary arterial hypertension,  n=46 | BAI | **BDI**  26% of outpatients with pulmonary arterial hypertension had moderate to severe depression (BDI score ≥ 17) | **7** |
| Cleland et al., 2007 | UK | Cross-sectional and observational study | Chronic obstructive pulmonary disease | Outpatients with chronic obstructive pulmonary disease, n=110 | HADS-A | **HADS-D**  20.8% of patients with chronic obstructive pulmonary disease reported probable clinically significant depression level (HADS-D score ≥ 11) | **7** |
| Engin et al., 2007 | Turkey | Cross-sectional and case-control study | Chronic idiopathic urticarial | Outpatients with chronic idiopathic urticarial,  n=73  healthy subjects,  n=34 | BAI | **BDI**  Chronic idiopathic urticarial outpatients had higher levels of depression than healthy subjects (BDI mean ± SD: 10.56 ± 7.90; 4.42±4.91, p< 0.05) | **7** |
| Korostil et al., 2007 | Toronto | Cross-sectional and observational study | Multiple sclerosis | Outpatients with multiple sclerosis, n=140 | SCID for DSM-IV  HADS-A | **HADS-D**  10.7% of outpatients with multiple sclerosis had depressive symptoms (HADS-D score ≥ 10) | **7** |
| Reference | **Country/City** | **Study Design** | **Diagnosis** | **Sample size** | **Instrument to assess anxiety** | **Psychopathological manifesttaions co-occurring with anxiety** | **JBI Checklist-total score** |
| Mondolo et al., 2007 | Italy | Cross-sectional and observational study | Parkinson’s disease | Outpatients with Parkinson’s disease, n=46 | STAI  HADS-A  HAM-A |  | **7** |
| Montin et al., 2007 | Finland | Longitudinal and observational study | Hip osteoarthritis | Outpatients with hip osteoarthritis,  n=100 | STAI |  | **7** |
| Todaro et al., 2007 | Rhode Island | Cross-sectional and observational study | Coronary heart disease | Outpatients with coronary heart disease,  n=150 | ADIS-IV for DSM-IV | **ADIS-IV for DSM-IV**  0.7% of outpatients with coronary heart disease had an obsessive-compulsive disorder  2% of outpatients with coronary heart disease had a post-traumatic stress disorder | **6** |
| Wasan et al., 2007 | Boston | Cross-sectional and observational study | Chronic rhino sinusitis | Outpatients with chronic rhino sinusitis, n=147 | HADS-A | **HADS-D**  9.1% of outpatients with chronic rhino sinusitis had moderate levels of depression  14.7% of outpatients with chronic rhino sinusitis outpatients reported high levels of depression | **7** |
| Wicks et al., 2007 | UK | Cross-sectional and observational study | Amyotrophic lateral sclerosis | Inpatients with amyotrophic lateral sclerosis,  n=190 | HADS-A | **BDI**  37% of inpatients with amyotrophic lateral sclerosis had mild to-moderate depression (BDI score 10-18)  13% of inpatients with amyotrophic lateral sclerosis had moderate to -severe depression (BDI score 19-29)  6% of inpatients with amyotrophic lateral sclerosis had severe depression (BDI score 30-63)  **HADS-D**  13% of inpatients with amyotrophic lateral sclerosis had borderline levels of depression (HASD-D score 8-10)  13% of inpatients with amyotrophic lateral sclerosis had depressive symptoms (HASD score 11- 21) | **7** |
| Reference | **Country/City** | **Study Design** | **Diagnosis** | **Sample size** | **Instrument to assess anxiety** | **Psychopathological manifesttaions co-occurring with anxiety** | **JBI Checklist-total score** |
| Wilson et al., 2007 | Canada | Cross-sectional and observational study | Cancer (palliative care) | Inpatients/outpatients cancer,  n=381 | PRIME-MD for DSM-IV | **PRIME-MD for DSM-IV**  13.1% of patients with cancer had a major depressive disorder  4.5% of patients with cancer had a dysthymia disorder | **8** |
| Azad et al., 2008 | Pakistan | Cross-sectional and observational study | Rheumatic diseases | Outpatients with rheumatic diseases, n=108 | HADS-A | **HADS-D**  56.5% of outpatients with rheumatic diseases had depressive symptoms (HADS-D score ≥ 9) | **7** |
| Beiske et al., 2008 | Norway | Cross-sectional and case-control study | Multiple sclerosis | Outpatients with multiple sclerosis, n=140  general population, n=1691 | HSCL-25 | **HSCL-25**  31% of outpatients with multiple sclerosis had depressive symptoms (HSCL-25 score ≥ 1.75) | **8** |
| Cukor et al., 2008 | Brooklyn | Cross-sectional and observational study | End-stage renal disease receiving hemodialysis | Outpatients with renal disease,  n=70 | SCID for DSM-IV  HADS-A | **SCID** **for DSM-IV**  29% of outpatients with renal disease had a major depressive disorder  19% of outpatients with renal disease had a substance-related disorder  10.2% of outpatients with renal disease had a psychotic disorder  11% of outpatients with renal disease had anxiety and depression disorders | **7** |
| Reference | **Country/City** | **Study Design** | **Diagnosis** | **Sample size** | **Instrument to assess anxiety** | **Psychopathological manifesttaions co-occurring with anxiety** | **JBI Checklist-total score** |
| Dogar et al., 2008 | Pakistan | Cross-sectional and observational study | Cardiovascular disease | Inpatients with cardiovascular disease,  n=100 | Clinical interview for DSM-IV criteria | **DSM-IV criteria**  47% of inpatients with cardiovascular disease had a depressive disorder  5% of inpatients with cardiovascular disease had anxiety and depressive disorders | **6** |
| Havermans et al., 2008 | Belgium | Cross-sectional and observational study | Cystic fibrosis | Outpatients with cystic fibrosis,  n=57 | HADS-A | **HADS-D**  13% of outpatients with cystic fibrosis had borderline/clinical depressive symptoms | **7** |
| Hawamdeh, 2008 | Jordan | Cross-sectional and observational study | Lower limb amputation | Outpatients with lower limb amputation,  n=56 | HADS-A | **HADS-D**  20% of outpatients with a lower limb amputation had depressive symptoms (HADS-D score ≥ 8) | **7** |
| Means-Christensen et al., 2008 | USA | Cross-sectional and observational study | Chronic pain (i.e., muscle pain, headache, stomach pain) | Outpatients with chronic pain,  n=113 | CIDI-Auto, version 2.1 for DSM-IV | **CIDI-Auto, version 2.1 for DSM-IV**  36.3% of outpatients with chronic pain had a major depressive disorder | **7** |
| Tang et al., 2008 | China | Cross-sectional and observational study | Chronic obstructive pulmonary disease | Inpatients with chronic obstructive pulmonary disease, n=166 | HADS-A |  | **7** |
| Reference | **Country/City** | **Study Design** | **Diagnosis** | **Sample size** | **Instrument to assess anxiety** | **Psychopathological manifesttaions co-occurring with anxiety** | **JBI Checklist-total score** |
| Bachen et al., 2009 | USA | Cross-sectional and case-control study | Systemic lupus erythematosus | Outpatients with systemic lupus erythematosus,  n=326  healthy subjects, n=3618 | CIDI, for DSM-IV | **CIDI** **for DSM-IV**  47% of outpatients with systemic lupus erythematosus had a major depressive disorder  6% of outpatients with systemic lupus erythematosus had a bipolar I disorder (formerly manic-depressive disorder)  3% of outpatients with systemic lupus erythematosus had a dysthymic disorder | **8** |
| Castro et al., 2009 | Brazil | Cross-sectional and observational study | Chronic pain | Outpatients with chronic pain,  n=400 | M.I.N.I. PLUS for DSM-IV | **M.I.N.I. PLUS for DSM-IV**  42% of outpatients with chronic pain had a major depressive episode  54% outpatients with chronic pain had a dysthymic disorder  26.5% outpatients with chronic pain had a risk of suicide (48% had a low risk of suicide, 10% had a moderate of risk suicide, 42% had a high risk of suicide)  1.8% outpatients with chronic pain had mixed anxiety and depression  10.8% outpatients with chronic pain had hypochondria  10.3% outpatients with chronic pain had a body dysmorphic disorder  5.8% outpatients with chronic pain had a premenstrual dysphoric disorder with dysmenorrhea  4.8% outpatients with chronic pain had a psychotic disorder  3.5% outpatients with chronic pain had alcohol abuse/addiction  2.3% outpatients with chronic pain had bulimia | **8** |
| Reference | **Country/City** | **Study Design** | **Diagnosis** | **Sample size** | **Instrument to assess anxiety** | **Psychopathological manifesttaions co-occurring with anxiety** | **JBI Checklist-total score** |
| Chan et al., 2009 | Singapore | Cross-sectional and observational study | Chronic obstructive pulmonary disease | Inpatients with chronic obstructive pulmonary disease, n=51 | PHQ of the PRIME-MD | **PHQ of the PRIME-MD**  7.8% of inpatients with chronic obstructive pulmonary disease had a major depressive disorder | **7** |
| Cordina et al., 2009 | Malta | Cross-sectional and observational study | Asthma | Outpatients with asthma,  n=201 | BAI |  | **7** |
| Delgado-Guay et al., 2009 | USA | Cross-sectional and observational study | Cancer (palliative care) | Outpatients with cancer,  n=216 | HADS-A | **HADS-D**  37% outpatients with cancer had depressive mood (HADS-D  Score ≥ 8) | **7** |
| Gallagher et al., 2009 | Sydney | Longitudinal and observational study | Coronary artery bypass graft | Outpatients/ inpatients having coronary artery bypass grafts  before surgery,  n=155  outpatients/ inpatients having coronary artery bypass grafts  after surgery,  n=132  outpatients/ inpatients having coronary artery bypass grafts  after 2 weeks  after discharge,  n=126 | HADS-A | **HADS-D**  Depression increased across time (F = 27.03, p < 0.001). Increases in depression occurred from before to after surgery (p < 0.05) and from after surgery to the second week after discharge (p < 0.001)  16% of patients had borderline or clinically signiﬁcant levels of depression (HADS-D scores ≥ 8) before the surgery  18.2% of patients had borderline or clinically signiﬁcant levels of depression (HADS-D scores ≥ 8) after the surgery  More than 45% of patients had borderline or clinically signiﬁcant levels of depression (HADS-D score ≥8) after hospital discharge | **7** |
| Reference | **Country/City** | **Study Design** | **Diagnosis** | **Sample size** | **Instrument to assess anxiety** | **Psychopathological manifesttaions co-occurring with anxiety** | **JBI Checklist-total score** |
| Honarmand et al., 2009 | Canada | Cross-sectional and observational study | Multiple sclerosis | Outpatients with multiple sclerosis, n=180 | SCID-I for  DSM-IV | **SCID-I for DSM-IV**  16.1% of outpatients with multiple sclerosis had a major depression disorder  5.7% of outpatients with multiple sclerosis had an obsessive-compulsive disorder  2.1% of outpatients with multiple sclerosis had post-traumatic stress disorder | **7** |
| Klarić et al., 2009 | Bosnia and Herzegovina | Cross-sectional and case-control study | End-stage renal disease receiving haemodialysis | Outpatients with chronic haemodialysis, n=56  outpatients with chronic illnesses,  n=53  subjects without chronic illnesses,  n=51 | STAI | **BDI**  Outpatients with chronic haemodialysis had a higher rate of depression (51.8%) compared to outpatients with chronic but not kidney diseases (41.5%) and subjects without chronic illnesses (9.8%) | **7** |
| Kovacs et al., 2009 | Ontario and Florida | Cross-sectional and observational study | Congenital heart disease | Outpatients with congenital heart disease,  n=280 | STAI-T  SCID, for  DSM-IV | **BDI-II**  On the whole sample the mean BDI-II score was 8.4 ± 9.4  10% of outpatients with congenital heart disease had mild symptoms (BDI-II score 14-19)  12% of outpatients with congenital heart disease had moderate to severe symptoms (BDI-II score ≥ 20)  **SCID**  19% of outpatients with congenital heart disease met criteria for a mood disorder | **7** |
| Reference | **Country/City** | **Study Design** | **Diagnosis** | **Sample size** | **Instrument to assess anxiety** | **Psychopathological manifesttaions co-occurring with anxiety** | **JBI Checklist-total score** |
| Quelhas et al., 2009 | Portugal | Cross-sectional and observational study | Parkinson’s disease | Outpatients with Parkinson’s disease, n=43 | HADS-A | **HADS-D**  58% of outpatients with Parkinson’s disease had depressive symptoms (HADS-D cut off value 8)  84% outpatients with Parkinson’s disease had moderate to severe depression (HADS-D cut off value 11) | **7** |
| Romão et al., 2009 | Brazil | Cross-sectional and case-control study | Chronic pelvic pain | Outpatients chronic pelvic pain,  n=52  healthy subjects,  n=54 | HADS-A | **HADS-D**  Outpatients with chronic pelvic pain had higher rates of depressive symptoms than healthy subjects (40% vs 30%, p < 0.05) | **7** |
| Serber et al., 2009 | Rhode Island | Cross-sectional and observational study | Coronary heart disease | Outpatients with coronary heart disease,  n=143 | ADIS-IV, for DSM-IV | **ADIS-IV** **for DSM-IV**  7.7% of outpatients with coronary heart disease had depression and anxiety disorders  3.5% of outpatients with coronary heart disease had a major depressive disorder  4.2% of outpatients with coronary heart disease had a dysthymic disorder | **7** |
| Axford et al., 2010 | UK | Cross-sectional and observational study | Lower limb osteoarthritis | Outpatients with lower limb osteoarthritis,  n=54 | HADS-A | **HADS-D**  27.7% of outpatients with lower limb osteoarthritis had depressive symptoms  18.5% of outpatients with lower limb osteoarthritis had depression and anxiety | **7** |
| Reference | **Country/City** | **Study Design** | **Diagnosis** | **Sample size** | **Instrument to assess anxiety** | **Psychopathological manifesttaions co-occurring with anxiety** | **JBI Checklist-total score** |
| Bellin et al., 2010 | USA | Cross-sectional and observational study | Spine bifida | Outpatients with spine bifida,  n=61 | HSCL-25 | **HSCL-25**  53.3% of outpatients with spine bifida had HSCL-25 scores above the clinical cut-off point for both depressive and anxiety symptoms  33.3% of outpatients with spine bifida had HSCL-25 **s**cores in the clinical range for depressive symptoms | **7** |
| Bossola et al., 2010 | Italy | Cross-sectional and observational study | End-stage renal disease | Outpatients with end-stage renal disease receiving chronic haemodialysis,  n=80 | HAM-A  SCL-90-R | **BDI**  52.5% of outpatients with end-stage renal disease receiving chronic haemodialysis had depressive symptoms | **7** |
| Dirik et al., 2010 | Turkey | Cross-sectional and observational study | Rheumatoid arthritis | Inpatients/ outpatients with rheumatoid arthritis,  n=117 | HADS-A | **HADS-D**  55.6% of patients with rheumatoid arthritis had possible depression (HADS-D score ≥ 8) | **8** |
| Dissanayaka et al., 2010 | Australia | Cross-sectional and observational study | Parkinson’s disease | Outpatients with Parkinson’s disease, n=79 | M.I.N.I. plus for DSM-IV  STAI | **M.I.N.I. plus DSM-IV**  11% of outpatients with Parkinson’s disease had a major depressive disorder  6% of outpatients with Parkinson’s disease had a dysthymic disorder  6% of outpatients with Parkinson’s disease had minor depression  4% of outpatients with Parkinson’s disease had risk of suicide  14% of outpatients with Parkinson’s disease had depressive disorders (major depressive disorder or dysthymia) and anxiety disorders | **7** |
| Reference | **Country/City** | **Study Design** | **Diagnosis** | **Sample size** | **Instrument to assess anxiety** | **Psychopathological manifesttaions co-occurring with anxiety** | **JBI Checklist-total score** |
| Du-Quiton et al., 2010 | USA | Cross-sectional and observational study | Advanced non-small cell lung cancer | Outpatients with advanced non-small cell lung cancer,  n=25  inpatients with advanced non-small cell lung cancer,  n=31 | HADS-A | **HADS-D**  25% of outpatients with advanced non-small cell lung cancer outpatients had depressive symptoms (HADS-D score ≥ 8)  25% of inpatients with advanced non-small cell lung cancer outpatients had depressive symptoms (HADS-D score ≥ 8) | **7** |
| Fava et al., 2010 | Italy | Cross-sectional and observational study | Functional gastrointestinal disorders, heart disease, cancer, endocrine disorders, skin disorders | Outpatients with functional gastrointestinal disorders, heart disease, cancer, endocrine disorders, skin disorders,  n=1660 | SCID for DSM-IV | **SCID for DSM-IV**  1.6% of outpatients had obsessive-compulsive disorder | **8** |
| Giardino et al., 2010 | USA | Cross-sectional and observational study | Chronic obstructive pulmonary disease  with emphysema | outpatients with chronic obstructive pulmonary disease  with emphysema, n=1828 | STAI | **BDI**  41% of outpatients with chronic obstructive pulmonary disease with emphysema had mild to moderate depression (BDI score ≥ 10)  8% of outpatients with chronic obstructive pulmonary disease with emphysema had moderate to severe depression (BDI score ≥ 19)  4% of outpatients with chronic obstructive pulmonary disease with emphysema had moderate to severe depression (BDI score ≥ 22)  Women had significantly more severe depression than men (BDI mean: 10.3 vs. 9.0, p < 0.001) | **8** |
| Reference | **Country/City** | **Study Design** | **Diagnosis** | **Sample size** | **Instrument to assess anxiety** | **Psychopathological manifesttaions co-occurring with anxiety** | **JBI Checklist-total score** |
| Karakoyun-Celik et al., 2010 | Turkey | Cross-sectional and observational study | Breast cancer | Outpatients with breast cancer,  n=120 | STAI | **BDI** 17.6% of outpatients with breast cancer had moderate depression (BDI score 9- 17)  19.2% of outpatients with breast cancer had severe depression (BDI score > 17) | **7** |
| Santos et al., 2010 | Brazil | Cross-sectional and observational study | Orthotropic liver transplantation | Orthotropic liver transplantation outpatients and inpatients,  n=215 | BAI |  | **7** |
| Ter Kuile et al., 2010 | The Netherlands | Cross-sectional and case-control study | Chronic pelvic pain (CPP) | Outpatients with chronic pelvic pain, n=154  healthy subjects,  n=58 | HADS-A | **HADS-D**  Women with chronic pelvic pain had higher levels of pain than healthy subjects (HADS-D mean ± SD: 50.10 ± 24.02 vs 2.13 ± 5.35)  Women with chronic pelvic pain had more severe depression than healthy subjects (HADS-D mean ± SD: 6.05 ± 4.24 vs 2.26 ± 2.24)  Women with chronic pelvic pain had signiﬁcantly more vagina complaints, sexual avoidance, non-sensuality, and sexual dissatisfaction than age-matched healthy subjects | **7** |
| Reference | **Country/City** | **Study Design** | **Diagnosis** | **Sample size** | **Instrument to assess anxiety** | **Psychopathological manifesttaions co-occurring with anxiety** | **JBI Checklist-total score** |
| Thijssen et al., 2010 | The Netherlands | Cross-sectional and observational study | Irritable bowel syndrome | Outpatients with irritable bowel syndrome,  n=230 | HADS-A | **HADS-D**  22% of outpatients with irritable bowel syndrome had possible depression (HADS-D score ≥ 8). Among irritable bowel syndrome outpatients, possible depression was more common in males than in females (35% vs 18%, p < 0.001)  14% outpatients with irritable bowel syndrome had anxiety and depressive symptoms | **7** |
| Tosic-Golubovic et al., 2010 | Serbia | Cross-sectional and case-control study | Irritable bowel syndrome | Outpatients with irritable bowel syndrome,  n=30  inpatients suffering from a depressive episode,  n=30  healthy subjects,  n=30 | HAM-A | **HAM-D and** **SDS**  Inpatients with a depressive episode (HAM-D mean ± SD: 26.63 ± 5.36, SDS mean ± SD: 0.72 ± 0.12) had more severe depressive symptoms than outpatients with irritable bowel syndrome  (HAM-D mean ± SD 20.23± 5.71; SDS mean ± SD: 0.62± 0.10) (p < 0.001)  Inpatients with a depressive episode and outpatients with irritable bowel syndrome had more  more severe depressive than healthy subjects (HAM-D mean ± SD 7.63± 4.0; SDS mean ± SD: 0.37± 0.06) (p < 0.001) | **7** |
| Alok et al., 2011 | India | Cross-sectional and case-control study | Fibromyalgia | Outpatients with fibromyalgia,  n=60  healthy subjects,  n=60 | DASS-21 | **DASS-21**  Outpatients with fibromyalgia had a higher rate of depression than healthy subjects (88.3% vs 5%) | **7** |
| Reference | **Country/City** | **Study Design** | **Diagnosis** | **Sample size** | **Instrument to assess anxiety** | **Psychopathological manifesttaions co-occurring with anxiety** | **JBI Checklist-total score** |
| Asadi-Pooya et al., 2011 | Philadelphia | Cross-sectional and observational study | Epilepsy | Outpatients/ inpatients with epilepsy,  n=200 | HADS-A | **HADS-D**  9.5% of outpatients and inpatients with epilepsy had depressive symptoms (HADS-D score >10) | **7** |
| Daştan, 2011 | Istanbul | Cross-sectional and observational study | Breast cancer | Outpatients with breast cancer,  n=123 | HADS-A | **HADS-D**  13.8% of breast cancer outpatients had borderline levels of depressive disorders (HADS-D score 8-10)  3.2% of breast cancer outpatients had severe depression (HADS-D score ≥ 11) | **7** |
| Guidi et al., 2011 | Italy | Cross-sectional and observational study | Functional gastrointestinal disorders, heart disease, cancer, endocrine disorders, skin disorders | Outpatients with functional gastrointestinal disorders, heart disease, cancer, endocrine disorders, skin disorders,  n=1,560 | SCID for DSM-IV | **SCID for DSM-IV**  12.7% of outpatients had a major depressive disorder  5% of outpatients had a somatoform disorder  3.5% of outpatients had an adjustment disorder | **8** |
| Häuser et al., 2011 | Germany | Cross-sectional and observational study | Inflammatory bowel disease (Crohn’s disease, ulcerative colitis) | Outpatients with Crohn’s disease,  n=314  outpatients with ulcerative colitis,  n=108 | HADS-A | **HADS-D**  16.6% of outpatients with Crohn’s disease and 20.4% of outpatients with ulcerative colitis had potential depressive disorder (HADS-D score 8-10)  9.2% of outpatients with Crohn’s disease and 13% of outpatients with ulcerative colitis had probable depressive disorder (HADS-D score ≥ 11) | **7** |
| Reference | **Country/City** | **Study Design** | **Diagnosis** | **Sample size** | **Instrument to assess anxiety** | **Psychopathological manifesttaions co-occurring with anxiety** | **JBI Checklist-total score** |
| Ho et al., 2011 | Singapore | Cross-sectional and observational study | Rheumatoid arthritis | Outpatients with rheumatoid arthritis, n=100 | HADS-A | **HADS-D**  15% of outpatients with rheumatoid arthritis had depressive symptoms (HADS-D score≥8)  11% of outpatients with outpatients had anxiety and depressive symptoms | **7** |
| Leentjens et al., 2011 | USA  Europe  Australia | Cross-sectional and observational study | Parkinson’s disease | Outpatients with Parkinson’s disease, n=340 | M.I.N.I. for DSM-IV | **M.I.N.I.** **for DSM-IV**  7.6% of outpatients with Parkinson’s disease had a major depressive disorder  2% of outpatients with Parkinson’s had dysthymia  26% of outpatients with Parkinson’s had major depressive disorder and anxiety disorders  13% of outpatients with Parkinson’s had dysthymia and anxiety disorders  36% of outpatients with Parkinson’s had generalized anxiety disorder and major depressive disorder  29% of outpatients with Parkinson’s had agoraphobia and major depressive disorder  15% of outpatients with Parkinson’s had social phobia and major depressive disorder  20% of outpatients with Parkinson’s had panic disorder and major depressive disorder | **7** |
| Reference | **Country/City** | **Study Design** | **Diagnosis** | **Sample size** | **Instrument to assess anxiety** | **Psychopathological manifesttaions co-occurring with anxiety** | **JBI Checklist-total score** |
| Malakouti et al., 2011 | Iran | Cross-sectional and observational study | Chronic tinnitus | Outpatients with chronic tinnitus, n=400 | SCID-I for DSM-III-R | **SCID for DSM-III-R**  Females outpatients with chronic tinnitus, compared to males, had higher rates of major depression (40% vs 29.5%, p < 0.001), somatoform disorder (9.7% vs 2.5%, p < 0.001), and lower rates of bipolar (5.2 vs 6%, p < 0.001), substance use (0% vs 5.3%, p < 0.001), or adjustment disorders (2.6% vs 3.2%, p < 0.001) | **7** |
| Nahon et al., 2011 | France | Cross-sectional and observational study | Inflammatory bowel disease | Outpatients with inflammatory bowel disease,  n=1,663 | HADS-A | **HADS-D**  11% of outpatients with inflammatory bowel disease had clinical depressive symptoms (HADS-D score ≥ 11) | **8** |
| Nenadović et al., 2011 | Serbia | Cross-sectional and case-control study | Epilepsy | Outpatients with generalized epilepsy outpatients,  n=30  outpatients with epilepsy of temporal lobe,  n=30  outpatients with epilepsy of extra temporal localization,  n=30  healthy subjects,  n=30 | BAI |  | **7** |
| Reference | **Country/City** | **Study Design** | **Diagnosis** | **Sample size** | **Instrument to assess anxiety** | **Psychopathological manifesttaions co-occurring with anxiety** | **JBI Checklist-total score** |
| Ponarovsky et al., 2011 | Iran | Cross-sectional and case-control study | Chronic skin diseases | Outpatients with chronic skin diseases outpatients,  n=60  subjects with non-allergies, n=52 | HAM-A | **HAM-D**  HAM-D > 7 was more frequent among outpatients with chronic skin diseases than among subjects with non-allergic skin disorders (48.3% vs 23.1%)  **M.I.N.I. for DSM-IV**  Outpatients with chronic skin diseases had higher rates of depression and anxiety than subjects with non-allergic skin disorders (55.% vs 26.9%, p < 0.01) | **7** |
| Robbins et al., 2011 | USA | Cross-sectional and observational study | Episodic and chronic migraine | Outpatients with episodic migraine, n=32  outpatients with chronic migraine, n=17 | GAD-7 | **PHQ-9**  Outpatients with episodic and chronic migraine did not differ for depression (PHQ-9 mean ± SD: 3.1 ± 4.1 vs 3.7 ± 4.9, p > 0.05)  Rates of current depression were relatively uncommon in both the episodic (6.3%) and chronic (11.8%) groups | **7** |
| Schaefer et al., 2011 | USA | Cross-sectional and observational study | Fibromyalgia | Outpatients with fibromyalgia,  n=203 | HADS-A | **HADS-D**  57.6% of outpatients with fibromyalgia had depressive symptoms  **MOS-SS**  23.2% of outpatients with fibromyalgia had major depressive disorder  68% of outpatients with fibromyalgia had sleep disturbances/insomnia | **7** |
| Reference | **Country/City** | **Study Design** | **Diagnosis** | **Sample size** | **Instrument to assess anxiety** | **Psychopathological manifesttaions co-occurring with anxiety** | **JBI Checklist-total score** |
| Shim et al., 2011 | South Korea | Cross-sectional and observational study | Cancer (i.e., colorectal, stomach, breast, lung cancer, lymphoma, osteosarcoma) | Inpatients with  cancer,  n=131 | HADS-A | **SAHD**  71.8% of inpatients with cancer experienced low desire for hastened death (SAHD score 1-4)  13.7% of inpatients with cancer experienced moderate desire for hastened death (SAHD score 5-9)  1.7% of inpatients with cancer experienced high desire for hastened death (SAHD score ≥ 10)  **HADS-D**  39.5% of inpatients with cancer had depressive symptoms (HADS-D score ≥ 8) and experienced high desire for hastened death | **7** |
| Sonino et al., 2011 | Italy | Cross-sectional and case-control study | Primary aldosteronism or essential hypertension | Outpatients with primary aldosteronism,  n=23  outpatients with essential hypertension,  n=23  healthy subjects,  n=23 | SCID for DSM-IV | **SCID for DSM-IV**  21.7% of outpatients with primary aldosteronism had a major depressive disorder  4.3% of outpatients with primary aldosteronism had a cyclothymic disorder  4.3% of outpatients with primary aldosteronism had a dysthymic disorder | **7** |
| Vistad et al., 2011 | Oslo | Cross-sectional and case-control study | Chronic pelvic pain in cervical cancer survivors | Cancer survivors with chronic pelvic pain, n=35  cancer survivors without chronic pelvic, n=56 | HADS-A | **HADS-D**  22% of cancer survivors with chronic pelvic pain had depressive symptoms (HADS-D score ≥ 8) | **8** |
| Reference | **Country/City** | **Study Design** | **Diagnosis** | **Sample size** | **Instrument to assess anxiety** | **Psychopathological manifesttaions co-occurring with anxiety** | **JBI Checklist-total score** |
| Von Leupoldt et al., 2011 | Germany | Longitudinal and observational study | Chronic obstructive pulmonary disease | Outpatients with chronic obstructive pulmonary disease, n=238 | HADS-A | **HADS-D**  30% of outpatients with chronic obstructive pulmonary disease had depression symptoms (HADS-D score ≥ 8)  Depression was associated with an increased dyspnoea and reduced functional performance and quality of life  Outpatients with chronic obstructive pulmonary disease reported more severe depression before the pulmonary rehabilitation than to the post rehabilitation (HADS-D mean ± SD: 6 ± 3.8 vs 5.6 ± 3.8, p < 0.01) | **7** |
| Asghari et al., 2012 | Iran | Cross-sectional and observational study | Obstructive sleep apnoea | Outpatients with obstructive sleep apnoea,  n=685 | BAI | **BDI**  Among outpatients with obstructive sleep apnoea, females had higher rates of depressive symptoms than males (70% vs 49%)  22.6% of outpatients with obstructive sleep apnoea had mild depression (BDI score 10-15)  19.1% of outpatients with obstructive sleep apnoea had moderate depression (BDI score 16- 23)  12.6% of outpatients with obstructive sleep apnoea had severe depression (BDI score ≥ 24) | **8** |
| Cheung et al., 2012 | New Zealand | Cross-sectional and observational study | Chronic obstructive pulmonary disease | Outpatients with chronic obstructive pulmonary disease,  n=55 | M.I.N.I. for DSM-IV  HADS-A | **M.I.N.I. for DSM-IV**  7.3% of outpatients with chronic obstructive pulmonary disease had dysthymic disorder  3.6% of outpatients with chronic obstructive pulmonary disease had a major depressive episode | **7** |
| Reference | **Country/City** | **Study Design** | **Diagnosis** | **Sample size** | **Instrument to assess anxiety** | **Psychopathological manifesttaions co-occurring with anxiety** | **JBI Checklist-total score** |
| Damen et al., 2012 | The Netherlands | Cross-sectional and observational study | Heart failure | Outpatients with heart failure,  n=237 | HADS-A |  | **7** |
| De Kort et al., 2012 | Maastricht | Cross-sectional and case-control study | Type 1 and type 2 diabetes | Outpatients with diabetes, n=280  healthy subjects, n=355 | HADS-A | **HADS-D**  Outpatients with diabetes had higher rates of depressive symptoms than healthy subjects (19.6% vs. 13.4%, p < 0.05) (HADS-D score ≥ 8) | **8** |
| Jones et al., 2012 | UK | Cross-sectional and observational study | Multiple sclerosis | Outpatients with multiple sclerosis, n=4,178 | HADS-A | **HADS-D**  46.9% outpatients with multiple sclerosis had depressive symptoms (HADS score ≥ 8)  Among outpatients with multiple sclerosis, males were more frequently depressed than females (50.6% vs 45.3%) | **8** |
| Khoury et al., 2012 | Lebanon | Cross-sectional and observational study | β-Thalassemia major and intermedia | Outpatients with β-thalassemia major and intermedia,  n=80 | STAI | **BDI**  35% of outpatients with β-thalassemia major and intermedia had depression:  18% of outpatients with β-thalassemia major and intermedia had mild depression (BDI score 10-15)  6.2% of outpatients with β-thalassemia major and intermedia had mild to moderate depression (BDI score 16-19)  8.8% of outpatients with β-thalassemia major and intermedia had moderate to severe depression (BDI score 20-29)  1.2% of outpatients with β-thalassemia major and intermedia had severe depression (BDI score 30-63) | **7** |
| Reference | **Country/City** | **Study Design** | **Diagnosis** | **Sample size** | **Instrument to assess anxiety** | **Psychopathological manifesttaions co-occurring with anxiety** | **JBI Checklist-total score** |
| Lewko et al., 2012 | Poland | Cross-sectional and observational study | Type 2 diabetes | Outpatients with type 2 diabetes, n=126 | HADS-A | **HADS-D**  32% of outpatients with type 2 diabetes had depression (HADS-D score ≥ 8)  Among outpatients with type 2 diabetes, females and males had comparable rates of depression (22.2% vs 10.3%, p > 0.05) (HADS-D score ≥ 8) | **8** |
| Preljevic et al., 2012 | Norway | Cross-sectional and observational study | Haemodialysis and peritoneal dialysis outpatients | Haemodialysis outpatients, n=84 peritoneal dialysis outpatients, n=25 | HADS-A | **SCID**  22% of haemodialysis and peritoneal dialysis outpatients had mood disorders (14.7% had a major depressive disorder, 3.7% a minor depression, 3.7% a dysthymic disorder)  37.5% of depressed outpatients had at least one anxiety disorder | **7** |
| Rafanelli et al., 2012 | Italy | Cross-sectional and observational study | Systemic arterial hypertension | Outpatients with systemic arterial hypertension,  n=125 | SCID-I for DSM-IV | **SCID-I for DSM-IV**  28% of outpatients with systemic arterial hypertension had mood disorders (19.2% had a minor depression, 4.8% had major depression disorder, 4% had dysthymic disorder) | **7** |
| Reference | **Country/City** | **Study Design** | **Diagnosis** | **Sample size** | **Instrument to assess anxiety** | **Psychopathological manifesttaions co-occurring with anxiety** | **JBI Checklist-total score** |
| Stewart et al., 2012 | Australia | Cross-sectional and observational study | Chronic hepatitis C | Outpatients with chronic hepatitis C, n=395 | HADS-A | **HADS-D**  27% of outpatients with chronic hepatitis C had depressive symptoms  45% of outpatients with chronic hepatitis C had depressive or anxiety symptoms  23% of outpatients with chronic hepatitis C had depressive and anxiety symptoms | **7** |
| Yildirim et al., 2012 | Turkey | Cross-sectional and case-control study | Chronic idiopathic urticarial | Outpatients with chronic idiopathic urticarial,  n=75  healthy subjects,  n=51 | BAI | **BDI**  Outpatients with chronic idiopathic urticarial had more severe depression than healthy subjects (BDI mean ± SD: 15.91 ± 9.12 vs 11.20 ± 11.07, p < 0.05) | **7** |
| Alacacioglu et al., 2013 | Turkey | Cross-sectional and case-control study | Cancer | Outpatients with cancer,  n=330  patients’ relatives, n=330 | STAI | **BDI**  Outpatients with cancer had more severe depression than their relatives (BDI mean ± SD: 14.7 ± 9.8 vs 10.3 ± 7.6, p < 0.001) | **8** |
| Del Rosso et al., 2013 | Italy | Cross-sectional and case-control study | Systemic sclerosis | Outpatients with systemic sclerosis, n=119  healthy subjects,  n=50 | HADS-A | **HADS-D**  Outpatients with systemic sclerosis had higher HADS-D score than healthy subjects (HADS-D mean ± SD: 6.14 ± 3.97 vs 4.72 ± 2.88, p < 0.05)  13% of outpatients with systemic sclerosis had depressive symptoms (HADS-D score ≥ 8)  23% of outpatients with systemic sclerosis had both depressive and anxious symptoms | **7** |
| Reference | **Country/City** | **Study Design** | **Diagnosis** | **Sample size** | **Instrument to assess anxiety** | **Psychopathological manifesttaions co-occurring with anxiety** | **JBI Checklist-total score** |
| DiNicola et al., 2013 | USA | Cross-sectional survey and observational study | Chronic obstructive pulmonary disease | Outpatients with chronic obstructive pulmonary disease, n=452 | HADS-A | **GDS-SF**  21.2% of outpatients with chronic obstructive pulmonary disease had depressive symptoms (GDS-SF score > 5) | **8** |
| Gullich et al., 2013 | Brazil | Cross-sectional and observational study | Medical diseases (i.e, hearth, respiratory, gastrointestinal, renal diseases, systemic arterial hypertension, cancer, HIV, obesity, diabetes, autoimmune disease) | Inpatients with medical diseases, n=284 | HADS-A |  | **7** |
| Katotomichelakis et al., 2013 | Greece | Cross-sectional and case-control study | Chronic rhino sinusitis (with or without nasal polyps) and allergic rhinitis | Outpatients with chronic rhino sinusitis (with or without nasal polyps) and allergic rhinitis,  n=108  healthy subjects,  n=30 | SAS, STAI | **SDS, BDI**  Outpatients with chronic rhino sinusitis, compared to healthy subjects, had higher SDS scores (SDS mean ±S D: 37.68 ± 8.56 vs 28.43 ± 5.57, p < 0.01) and BDI scores (BDI mean ± SD: 9.59 ± 7.89 vs 5.07 ± 3.56, p < 0.05) | **7** |
| Kayhan et al., 2013 | Turkey | Cross-sectional and observational study | Different chronic medical illness | Inpatients with chronic medical illness,  n=603 | SCID-I for DSM-IV | **SCID-I for DSM-IV**  14.3% of inpatients with chronic medical illness had a mood disorder (8.6% with major depressive disorder) | **8** |
| Lee et al., 2013 | Korea | Cross-sectional and observational study | End-stage renal disease | Outpatients with end-stage renal disease, n=208 | HADS-A | **HADS-D**  47.1% of outpatients with end-stage renal disease had depressive symptoms (HADS-D score ≥ 8)  24.5% of outpatients with end-stage renal disease had depression and anxious symptoms | **7** |
| Reference | **Country/City** | **Study Design** | **Diagnosis** | **Sample size** | **Instrument to assess anxiety** | **Psychopathological manifesttaions co-occurring with anxiety** | **JBI Checklist-total score** |
| Leite et al., 2013 | Brazil | Cross-sectional and observational study | Systemic sclerosis | Outpatients with systemic sclerosis,  n=128 | GAD-7 | **PHQ-9**  90% of outpatients with systemic sclerosis had a PHQ-9 score ≥ 3  **BDQ**  69% of outpatients with systemic sclerosis had a body image disturbance | **7** |
| Lincoln et al., 2013 | Belgium, UK, Switzerland, Germany | Longitudinal and observational study | Stroke | Outpatients with stroke,  n=220 | HADS-A | **HADS-D**  6 months after stroke, 20% had depression (HADS-D score ≥ 7)  5 years after stroke, 33% had depression (HADS-D score ≥ 7) | **8** |
| Okamoto et al., 2013 | Japan | Cross-sectional and observational study | Thoracic aortic surgery Coronary artery bypass grafting | Outpatients who underwent thoracic aortic surgery,  n=49  outpatients who underwent coronary artery bypass grafting, n=79 | HADS-A | **HADS-D**  27.6% of the outpatients who underwent thoracic aortic surgery and 20.2% of outpatients who underwent coronary artery bypass grafting were in the significant range of depression (score ≥ 8) | **7** |
| Sharma et al., 2013a | India | Cross-sectional and case-control study | Migraine | Outpatients with migraine,  n=71  healthy subjects,  n= 71 | HADS-A | **HADS-D**  Outpatients with migraine had more severe depressive symptoms than healthy subjects (6.5 ± 4.4 vs 3.2 ± 3.6, p < 0.05)  Outpatients with migraine, compared to healthy subjects, had higher rates of possible depressive symptoms (21.1% vs 11.3%, HADS-D score 8-10) and probable depressive symptoms (19.7% vs 2.8%, HADS-D score ≥ 11) | **7** |
| Reference | **Country/City** | **Study Design** | **Diagnosis** | **Sample size** | **Instrument to assess anxiety** | **Psychopathological manifesttaions co-occurring with anxiety** | **JBI Checklist-total score** |
| Sharma et al., 2013b | India | Cross-sectional and case-control study | Chronic respiratory disorders (i.e., allergic rhinitis, bronchial asthma, chronic obstructive pulmonary disease), | Outpatients with chronic respiratory disorders,  n=391  healthy subjects, n=177 | GMHAT/PC for ICD-10 | **GMHAT/PC for ICD-10**  Outpatients with chronic respiratory disorders had higher rates of depression (13.2% vs 3.4%) and obsessive compulsive disorder (4.6% vs 2.8%) than healthy subjects | **8** |
| Van den Heuvel et al., 2013 | Zambia | Cross-sectional and observational study | HIV and tuberculosis | outpatients with HIV and/or tuberculosis, n=649 | M.I.N.I. for DSM-IV | **M.I.N.I. for DSM-IV**  11.3% of outpatients with HIV and/or tuberculosis had a major depressive disorder  30.4% of outpatients with HIV and/or tuberculosis had suicidality (low severity in 82.2%, moderate in 10.5%, high in 7.2%)  Among those who endorsed suicidal behaviour, 5.9% had a suicidal ideation, 3.2% a suicide plan, 3% attempted suicide in the past  3.8% and 18% of outpatients with HIV and/or tuberculosis had alcohol abuse and alcohol dependence respectively | **8** |
| Wang et al., 2013a | China | Longitudinal and observational study | Coronary artery disease | Inpatients with coronary artery disease,  n=1007 | SAS |  | **8** |
| Reference | **Country/City** | **Study Design** | **Diagnosis** | **Sample size** | **Instrument to assess anxiety** | **Psychopathological manifesttaions co-occurring with anxiety** | **JBI Checklist-total score** |
| Wang et al., 2013b | China | Cross-sectional and observational study | Lymphoma | Outpatients with lymphoma,  n=323 | HADS-A | **HADS-D**  35.3% of outpatients with lymphoma had depressive symptoms (HADS-D score ≥ 9) | **7** |
| Wu et al., 2013 | Taiwan | Cross-sectional and observational study | Type 2 diabetes | Outpatients with type 2 diabetes,  n=201 | BAI | **BDI**  Among outpatients with type 2 diabetes, depression was found to be significantly negatively correlated with self-efﬁcacy  (r = -0.24, p < 0.01) and significantly positively correlated with illness complications (r = 0.27, p < 0.01) | **7** |
| Zhou et al., 2013 | China | Cross-sectional and observational study | Glaucoma | Outpatients with glaucoma,  n=506 | HADS-A | **HADS-D**  16.4% of outpatients with glaucoma had depression (HADS-D score > 10) | **8** |
| Alosaimi et al., 2014 | Saudi Arabia | Cross-sectional and observational study | Gastroenterological diseases (i.e., abdominal pain, heart burn, diarrhoea, constipation, appetite or weight changes, nausea, vomiting) | Outpatients with gastroenterological diseases,  n=440 | GAD‑7 | **PHQ-9**  36% of outpatients with gastroenterological diseases had depressive symptoms (PHQ-9 score ≥ 10)  23% of outpatients with gastroenterological disease had anxious and depressive symptoms | **7** |
| Anyfanti et al., 2014 | Greece | Cross-sectional and observational study | Rheumatic diseases | Outpatients with rheumatic diseases, n=514 | HAM-A | **SDS**  21.8% of outpatients with rheumatic diseases had depressive symptoms (SDS mean ± SD: 41.9 ± 8.9) | **7** |
| Reference | **Country/City** | **Study Design** | **Diagnosis** | **Sample size** | **Instrument to assess anxiety** | **Psychopathological manifesttaions co-occurring with anxiety** | **JBI Checklist-total score** |
| Askari et al., 2014 | Iran | Cross-sectional and observational study | Multiple sclerosis | Outpatients with multiple sclerosis, n=180 | BAI | **BDI**  Among outpatients with multiple sclerosis, BDI mean was 17.5 ± 11.4 Outpatients with higher levels of disability had significant higher BDI | **7** |
| Bragança et al., 2014 | Brazil | Cross-sectional and observational study | Primary hyperhidrosis | Outpatients with primary hyperhidrosis, n=197 | HADS-A | **HADS-D**  11.2% of outpatients with primary hyperhidrosis had depressive symptoms | **7** |
| Cordingley et al., 2014 | UK | Cross-sectional and observational study | Rheumatoid arthritis | Outpatients with rheumatoid arthritis, n=322 | HADS-A | **HADS-D**  47.3% of outpatients with rheumatoid arthritis had moderate depressive symptoms (HADS-D score >7)  20.6% of outpatients with rheumatoid arthritis had had severe depressive symptoms (HADS-D score > 11) | **7** |
| Daniëls et al., 2014 | The Netherlands | Cross-sectional and case-control study | Hodgkin lymphoma | Outpatients with Hodgkin lymphoma, n=180  healthy subjects, n=327 | HADS-A | **HADS-D**  Among outpatients with Hodgkin lymphoma, 18% had depressive symptoms compared with 12% in the control group (HADS-D score > 8) | **8** |
| Fouche et al., 2014 | USA | Cross-sectional and observational study | Hereditary angioedema | Outpatients with hereditary angioedema,  n=26 | HAM-A | **HAM-D**  39% of outpatients with hereditary angioedema had depressive symptoms 50% of outpatients with hereditary angioedema had mild depressive symptoms (HAM-D score 8.13)  40% of outpatients with hereditary angioedema had moderate depressive symptoms (HAM-D score 14-18)  10% of outpatients with hereditary angioedema had severe depressive symptoms (HAM-D score > 19) | **7** |
| Reference | **Country/City** | **Study Design** | **Diagnosis** | **Sample size** | **Instrument to assess anxiety** | **Psychopathological manifesttaions co-occurring with anxiety** | **JBI Checklist-total score** |
| Ganasegeran et al., 2014 | Malaysia | Cross-sectional and observational study | Type 2 diabetes | Outpatients with type 2 diabetes,  n=169 | HADS-A | **HADS-D**  29% of outpatients with type 2 diabetes had mild depressive symptoms (HADS-D score 8-10)  9.5% of outpatients with type 2 diabetes had moderate depressive symptoms (HADS-D score 11-14)  1.8% of outpatients with type 2 diabetes had severe depressive symptoms (HADS-D score 15-21) | **7** |
| Grzesiak et al., 2014 | Poland | Cross-sectional and case-control study | Irritable bowel syndrome, gastroesophageal reflux | Outpatients with irritable bowel syndrome,  n=106  outpatients with gastroesophageal reflux disease outpatients,  n=53 | CIDI for DSM-IV | **CIDI for DSM-IV**  22.6% of outpatients with irritable bowel syndrome had depressive and anxiety disorders  11.3% of outpatients with gastroesophageal reflux disease had depressive and anxious disorders | **7** |
| Holland et al., 2014 | Australia | Cross-sectional and observational study | Interstitial lung diseases | Outpatients with interstitial lung diseases,  n=124 | HADS-A | **HADS-D**  7% of outpatients with interstitial lung diseases had clinically significant depression level (HADS-D score ≥ 11)  23% of outpatients with interstitial lung diseases had borderline levels of depression (HADS-D score 8-10) | **7** |
| Reference | **Country/City** | **Study Design** | **Diagnosis** | **Sample size** | **Instrument to assess anxiety** | **Psychopathological manifesttaions co-occurring with anxiety** | **JBI Checklist-total score** |
| Hong et al., 2014 | China | Cross-sectional and observational study | Cancer | Outpatients with cancer,  n=1,217 | HADS-A | **HADS-D**  66.7 % of outpatients with cancer had depressive symptoms (HADS-D  score ≥ 11) | **8** |
| Kheirandish et al., 2014 | Iran | Cross-sectional and observational study | Systemic lupus erythematosus | Outpatients with systemic lupus erythematosus,  n=166 | Cattel Anxiety Self-assessment Scale | **BDI**  64.5% of outpatients with systemic lupus erythematosus had depression | **7** |
| Kugler, 2014 | Germany | Cross-sectional and observational study | Heart transplantation | Outpatients with heart transplantation,  n=203 | GAD-7 | **BDI-II**  14.8% of outpatients with heart transplantation had severe depressive symptoms (BDI-II score ≥ 14)  17.7% of outpatients with heart transplantation had borderline depressive symptoms (BDI-II score 9-13) | **7** |
| Liu et al., 2014 | China | Cross-sectional and observational study | Asthma | Outpatients with asthma,  n=261 | SAS | **SDS**  13.4% of outpatients with asthma had depressive symptoms  7.7% of outpatients with asthma had depressive and anxious symptoms | **7** |
| Macaron et al., 2014 | Lebanon | Cross-sectional and observational study | End-stage renal disease | Outpatients with end-stage renal disease, n=51 | HADS-A | **HADS-D**  50% of outpatients with end-stage renal disease had depressive symptoms  (HADS-D score > 7)  **Module C of the M.I.N.I. for DSM-IV**  37% of outpatients with end-stage renal disease had a suicidal ideation (31% had low risk of suicide, 6 % moderate risk of suicide) | **7** |
| Reference | **Country/City** | **Study Design** | **Diagnosis** | **Sample size** | **Instrument to assess anxiety** | **Psychopathological manifesttaions co-occurring with anxiety** | **JBI Checklist-total score** |
| Schlereth et al., 2014 | Germany | Cross-sectional and observational study | Post herpetic neuralgia | Outpatients with post herpetic neuralgia,  n=36 | HADS-A | **HADS-D**  The HADS-D mean was 5.9 ± 0.9  16.1% of outpatients with post herpetic neuralgia had clinical depressive symptoms (HADS-D score ≥ 11)  12.9% had borderline depressive levels (HADS-D score 8-10) | **7** |
| Schramm et al., 2014 | Germany | Cross-sectional and case-control study | Autoimmune hepatitis | Outpatients with autoimmune hepatitis, n=103  healthy subjects, n=3720 | GAD-7 | **PHQ-9**  Outpatients with autoimmune hepatitis had higher rates of depressive symptoms than healthy subjects (5.9% vs. 2.6%, p < 0.001)  Scoring for major depression syndrome was more frequent in outpatients with autoimmune hepatitis than in healthy subjects (10.8% vs. 1.9%, p < 0.001) | **8** |
| Sun et al., 2014 | China | Cross-sectional and observational study | AIDS | Outpatients with AIDS,  n=772 | SAS | **CES-D**  73.1% of outpatients with AIDS had depressive symptoms (CES-D score ≥ 16)  Females had higher rates of depressive symptoms than males (84% vs 71.8%, p < 0.05) | **7** |
| Turan et al., 2014 | Turkey | Cross-sectional and observational study | Chronic obstructive pulmonary disease | Outpatients with chronic obstructive pulmonary disease, n=78 | HADS-A | **HADS-D**  17.7% of outpatients with chronic obstructive pulmonary disease had high depressive symptoms  9.7% of outpatients with chronic obstructive pulmonary disease had high HADS scores | **7** |
| Reference | **Country/City** | **Study Design** | **Diagnosis** | **Sample size** | **Instrument to assess anxiety** | **Psychopathological manifesttaions co-occurring with anxiety** | **JBI Checklist-total score** |
| Turkistani et al., 2014 | Saudi Arabia | Cross-sectional and observational study | End-stage renal disease | End-stage renal disease  outpatients,  n=286 | HADS-A | **HADS-D**  21.5% of outpatients with end-stage renal disease had a borderline level of depression (HADS-D score 0-10)  23.3% of outpatients with end-stage renal disease had an pathological level of depression (HADS-D score 11-21) | **7** |
| Vanhoof et al., 2014 | Belgium | Cross-sectional and observational study | Pulmonary arterial hypertension | Outpatients with pulmonary arterial hypertension,  n=101 | DASS-21 | **DASS-21**  32.6% of outpatients with pulmonary arterial hypertension had depressive symptoms | **7** |
| Yang et al., 2014 | China | Cross-sectional and observational study | Acute coronary syndrome | Outpatients with acute coronary syndrome,  n=100 | HADS-A  M.I.N.I. for DSM-IV | **HADS-D**  Among outpatients with acute coronary syndrome those with depression had significantly higher HADS-D mean scores than those without depression (HADS-D mean ± SD: 11.5 ± 3.1 vs 4.9±3.0, p < 0.05)  **M.I.N.I. for DSM-IV**  38% of outpatients with acute coronary syndrome had a major depressive disorder  9% of outpatients with acute coronary syndrome had depressive and anxiety disorders | **7** |
| Reference | **Country/City** | **Study Design** | **Diagnosis** | **Sample size** | **Instrument to assess anxiety** | **Psychopathological manifesttaions co-occurring with anxiety** | **JBI Checklist-total score** |
| Zhang et al., 2014 | USA | Cross-sectional and case-control study | Maintenance haemodialysis | Outpatients undergoing maintenance haemodialysis,  n=72  healthy subjects,  n=39 | BAI  HADS-A | **BDI**  33% of outpatients undergoing maintenance haemodialysis had higher rates of depressive symptoms than healthy subjects (33% vs 5%, p < 0.001) (HADS-D score ≥ 14) | **7** |
| Alsaadi et al., 2015 | Abu Dhabi | Cross-sectional and case-control study | Epilepsy | Outpatients with epilepsy, n=186  healthy subjects, n=186 | GAD-7 | **PHQ-9**  27% of outpatients with epilepsy had a major depression (PHQ-9 score >10)  7.7% of outpatients with epilepsy had depressive and anxious symptoms | **7** |
| Beutel et al., 2015 | Germany | Cross-sectional and case-control study | Survivors of malignant melanoma | Survivors of malignant melanoma,  n=683  healthy subjects, n=4133 | GAD-7 | **PH**  Survivors of malignant melanoma had more severe depressive symptoms than healthy subjects (PH mean ± SD: 3.8 ± 4.0 vs. 2.3 ± 3.2)  Females had more severe depressive symptoms than males (PH mean ± SD: 4.3 ± 4.2 vs. 3.4 ± 3.8) | **8** |
| Cardoso et al., 2015 | Portugal | Cross-sectional and observational study | Cancer | Outpatients with cancer,  n=270 | HADS-A | **HADS-D**  24.1% of outpatients with cancer had depressive symptoms (HADS-D score ≥ 8) | **7** |
| Costa et al., 2015 | Portugal | Cross-sectional and observational study | Chronic pain (i.e., fibromyalgia, rheumatoid arthritis, chronic pain not otherwise specified) | Chronic pain outpatients,  n=200 | HADS-A | **HADS-D**  Females outpatients with chronic pain had more severe depressive symptoms than males (r = −.26; p <  0.01) | **7** |
| Reference | **Country/City** | **Study Design** | **Diagnosis** | **Sample size** | **Instrument to assess anxiety** | **Psychopathological manifesttaions co-occurring with anxiety** | **JBI Checklist-total score** |
| Değirmenci et al., 2015 | Turkey | Cross-sectional and case-control study | Obesity | Obese outpatients, n=52  healthy subjects,  n=43 | HAM-A | **HAM-D17**  Obese outpatients had more severe depressive symptoms than healthy subjects (HAM-D17 mean ± SD: 9.44 ± 4.76 vs 2.46 ± 3.34 p < 0.001) | **7** |
| Freitas et al., 2015 | Brazil | Cross-sectional and observational study | Inflammatory bowel disease (i.e., Crohn’s disease, ulcerative colitis) | Outpatients with Crohn’s disease,  n=64  outpatients with ulcerative colitis, n=83) | HADS-A | **HADS-D**  24.5% of outpatients with inflammatory bowel disease had depressive symptoms (HADS-D score ≥ 8)  23.4% of outpatients with Crohn’s disease had depressive symptoms  (HADS-D score ≥ 8)  25.3% of outpatients with ulcerative colitis had depressive symptoms  (HADS-D score ≥ 8) | **7** |
| Kayhan et al., 2015 | Turkey | Cross-sectional and case-control study | Chronic low back and neck pain caused by disc herniation | Outpatients with chronic low back and neck pain caused by disc herniation,  n=149  healthy subjects,  n=60 | SCID for DSM-IV | **SCID for DSM-IV**  16.9% of outpatients with chronic low back and neck pain caused by disc herniation had a major depressive disorder | **7** |
| Kouris et al., 2015 | Greece | Cross-sectional and case-control study | Chronic  hand eczema | Outpatients with chronic hand eczema, n=71  healthy subjects, n=71 | HADS-A | **HADS-D**  Outpatients with chronic hand eczema and healthy subjects did not differ for the severity of depressive symptoms (HADS-D mean ± SD: 7.32 ± 2.69 vs 6.73 ± 3.99, p > 0.05) | **7** |
| Reference | **Country/City** | **Study Design** | **Diagnosis** | **Sample size** | **Instrument to assess anxiety** | **Psychopathological manifesttaions co-occurring with anxiety** | **JBI Checklist-total score** |
| Lou et al., 2015 | China | Cross-sectional and observational study | Benign breast lumps | Outpatients with benign breast lumps, n=371 | SAS | **CES-D**  62% of outpatients with benign breast lumps had depressive symptoms (CES-D score ≥ 16);  37.5% had co-occurrence between anxiety and depressive symptoms | **8** |
| Ozturk et al., 2015 | Turkey | Cross-sectional and observational study | Coronary artery disease and coronary artery ectasia | Outpatients with coronary artery disease and coronary artery ectasia,  n=18  outpatients with coronary artery disease,  n=310 | STAI  BAI | **BDI**  Outpatients with coronary artery disease and coronary artery ectasia and outpatients with only coronary artery disease did not differ for severity of depressive symptoms (BDI mean ± SD: 7.6 ± 6.2 vs 10.3 ± 7.9, p > 0.05) | **7** |
| Rogers et al., 2015 | Colombia | Cross-sectional and observational study | Rheumatoid arthritis | Outpatients with rheumatoid arthritis, n=103 | STAI | **SDS**  Among outpatients with rheumatoid arthritis, SDS mean score was 44.14 ± 7.40  Higher levels of depression was associated with lower levels of quality of life (r = −0.72, p < 0.001) | **7** |
| Schwarz et al., 2015 | Florida | Longitudinal and observational study | Acute chest pain | Outpatients with acute chest pain, n=151 | GAD-7 |  | **7** |
| Teixeira et al., 2015 | Brazil | Cross-sectional and observational study | Systemic hypertension and diabetes | Outpatients with systemic hypertension,  n=17  outpatients with diabetes, n=17 | BAI | **BDI**  Among outpatients with systemic hypertension, DI mean score was 24 ± 13  Among outpatients with diabetes, BDI mean score was 23 ± 12 | **7** |
| Reference | **Country/City** | **Study Design** | **Diagnosis** | **Sample size** | **Instrument to assess anxiety** | **Psychopathological manifesttaions co-occurring with anxiety** | **JBI Checklist-total score** |
| Uçar et al., 2015 | Turkey | Cross-sectional and case-control study | Fibromyalgia | Outpatients with fibromyalgia,  n=95  healthy subjects,  n=95 | HAI-SF | **BDI**  Outpatients with fibromyalgia had more severe depressive symptoms than healthy subjects (BDI mean ± SD: 18.64 ± 10.11 vs 6.21 ± 4.05, p < 0.001) | **7** |
| Wan et al., 2015 | Singapore | Cross-sectional and observational study | Rheumatoid arthritis | Outpatients with rheumatoid arthritis, n=108 | HADS-A | **HADS-D**  13% of outpatients with rheumatoid arthritis had depressive symptoms (HADS-D score ≥ 8) | **9** |
| Weaver et al., 2015 | New Delhi | Cross-sectional and observational study | Type 2 diabetes | Outpatients with type 2 diabetes (only women),  n=184 | HSCL-25 | **HSCL-25**  19% of outpatients with type 2 diabetes had depressive symptoms | **7** |
| Yang et al., 2015 | China | Cross-sectional and case-control study | Gastroesophageal reflux disease (i.e., reflux esophagitis, non-erosive reflux disease) | Outpatients with reflux esophagitis,  n=134  non-erosive reflux disease outpatients, n=145  healthy subjects, n=100 | SAS | **SDS**  Outpatients with reflux esophagitis had more severe depressive symptoms than healthy subjects (SDS mean ± SD: 46.76 ± 11.83 vs 37.61 ± 8.44, p < 0.001)  Outpatients with non-erosive reflux disease had more severe depressive symptoms than healthy subjects (SDS mean ± SD: 49.65 ± 11.09 vs 37.61 ± 8.44,  p < 0.001) | **7** |
| Reference | **Country/City** | **Study Design** | **Diagnosis** | **Sample size** | **Instrument to assess anxiety** | **Psychopathological manifesttaions co-occurring with anxiety** | **JBI Checklist-total score** |
| Yilmaz-Oner et al., 2015 | Turkey | Cross-sectional and case-control study | Systemic lupus erythematosus | Outpatients with systemic lupus erythematosus,  n=113  healthy subjects, n=123 | HADS-A | **HADS-D**  Outpatients with systemic lupus erythematosus had more severe depressive symptoms than healthy subjects (HADS-D mean ± SD: 6.11 ± 4.72 vs. 5.12 ± 3.93)  Among outpatients with systemic lupus erythematosus, 16.8% had possible depression (HADS score 8-10), 15.9% probable depression (HADS score 11-14), 5.3% were extreme cases of depression HADS-D score 15-21) | **7** |
| Abuelgasim et al., 2016 | Saudi Arabia | Cross-sectional and observational study | Haematological cancer | Outpatients with haematological cancer, n=211 | GAD-7 | **PHQ-9**  46.5% of outpatients with haematological cancer had depressive symptoms (PHQ-9 score ≥ 10)  18.1% of outpatients with haematological cancer had anxious and depressive symptoms | **7** |
| Bahar et al., 2016 | Vancouver Shanghai | Cross-sectional and observational study | Hyperhidrosis and other skin conditions | Outpatients with hyperhidrosis, n=437  outpatients without hyperhidrosis, n=1580 | GAD-7 | **PHQ-9**  Outpatients with hyperhidrosis had higher rates of depressive symptoms than outpatients without hyperhidrosis (27.2% vs 9.7%, p < 0.001) | **8** |
| Bektas et al., 2016 | Turkey | Cross-sectional and observational study | Gastrointestinal cancer | Inpatients with gastrointestinal cancer, n=335 | SCL-90-R | **HADS-D**  Among outpatients with gastrointestinal cancer, HADS-D mean was 6.34 ± 4.85  Females had more severe depressive symptoms than males (HADS-D mean ± SD: 7.21 ± 4.75 vs 5.86 ± 4.85, p < 0.05)  Outpatients with stomach cancer had more severe depressive symptoms than outpatients with colon cancer (HADS-D mean ± SD: 7.04±4.48 vs 4.98 ± 4.27, p < 0.05) | **7** |
| Reference | **Country/City** | **Study Design** | **Diagnosis** | **Sample size** | **Instrument to assess anxiety** | **Psychopathological manifesttaions co-occurring with anxiety** | **JBI Checklist-total score** |
| Bertoli et al., 2016 | UK | Cross-sectional and observational study | Chronic temporomandibular disorders (i.e., muscle pain, joint pain) | Outpatients with muscle pain, n=534 outpatients with joint pain, n=246 outpatients with both muscle and joint pain, n=461 | SCL-90-R | **SCL-90-R**  30.4% of outpatients with chronic temporomandibular disorders had depressive symptoms | **8** |
| Carvalho et al., 2016 | Brazil | Cross-sectional and observational study | Cardiovascular disease | Inpatients with cardiovascular disease, n=120 | HADS-A | **HADS-D**  17.5% of inpatients with cardiovascular disease had depressive symptoms  Females had higher rates of depressive symptoms than males (66.67% vs 33.33%, p < 0.05) | **6** |
| Cumming et al., 2016 | Sweden | Cross-sectional and case-control study | Stroke survivors | Stroke survivor outpatients, n=149  healthy subjects, n=745 | Comprehensive Psychopathological Rating Scale (CPRS) semi-structured psychiatric interview for DSM-III-R |  | **6** |
| Dissanayaka et al., 2016 | Australia | Cross-sectional and observational study | Parkinson’s disease | Outpatients with Parkinson’s disease, n=90 | M.I.N.I. Plus, for DSM-IV | **M.I.N.I. Plus for DSM-IV**  10% of outpatients with Parkinson’s disease had anxiety/depressive disorders  4.4% of outpatients with Parkinson’s disease had a major depression disorder  27.8% of outpatients with Parkinson’s had a lifetime history of a major depression disorder  5.6% of outpatients with Parkinson’s had dysthymia  7.8% of outpatients with Parkinson’s had a life time history of dysthymia  12.2% of outpatients with Parkinson’s had a suicidality | **7** |
| Reference | **Country/City** | **Study Design** | **Diagnosis** | **Sample size** | **Instrument to assess anxiety** | **Psychopathological manifesttaions co-occurring with anxiety** | **JBI Checklist-total score** |
| Gostoli, et al., 2016 | Italy |  | Sudden cardiac death | Inpatients and outpatients undergoing implantable cardioverter defibrillator,  n=117 | SCID for DSM-IV-TR | **SCID DSM-IV-TR**  17.9% of cases had depressive disorder | **7** |
| Gu et al., 2016 | China | Longitudinal and case-control study | Coronary heart disease | Inpatients with coronary heart disease who underwent percutaneous coronary intervention, n=170 | HADS-A | **HADS-D**  23.5% of coronary heart disease inpatients had depressive symptoms 1 day before the surgery  44.7% of coronary heart disease inpatients had depressive symptoms 1 day after the surgery  48.8% of coronary heart disease inpatients had depressive symptoms 1 month after the surgery  44.7% of coronary heart disease inpatients had depressive symptoms 3 months after the surgery  28.2% of coronary heart disease inpatients had depressive symptoms 6 months after the surgery  21.8% of coronary heart disease inpatients had depressive symptoms 12 months after the surgery | **7** |
| Reference | **Country/City** | **Study Design** | **Diagnosis** | **Sample size** | **Instrument to assess anxiety** | **Psychopathological manifesttaions co-occurring with anxiety** | **JBI Checklist-total score** |
| Jamshidi et al., 2016 | Iran | Cross-sectional and observational study | Rheumatoid arthritis | Outpatients with rheumatoid arthritis, n=414 | Cattell’s 40-item questionnaire | **BDI**  23.8% of outpatients with rheumatoid arthritis had mild depressive symptoms (BDI score 11-16)  14.6% of outpatients with rheumatoid arthritis had borderline depressive symptoms (BDI score 17-20)  16.5% of outpatients with rheumatoid arthritis had moderate depressive symptoms (BDI score 21-30)  7.3% of outpatients with rheumatoid arthritis had severe depressive symptoms (BDI score 31-40)  1.5% of outpatients with rheumatoid arthritis had more severe depressive symptoms (BDI score > 40) | **7** |
| Kala et al., 2016 | Czech Republic | Longitudinal and observational study | Acute myocardial infarction | Inpatients/ outpatients with acute myocardial infarction treated with primary percutaneous coronary intervention, n=79 | SAS | **BDI-II** (score ≥ 14)  21.5% of cases had depressive symptoms within 24 hours after surgery  9.2% of cases had depressive symptoms before discharge  10.4% of cases had depressive symptoms at 3 months after surgery  15.4% of cases had depressive symptoms at 6 months after surgery  13.8% of cases had depressive symptoms at 12 months after surgery | **7** |
| Reference | **Country/City** | **Study Design** | **Diagnosis** | **Sample size** | **Instrument to assess anxiety** | **Psychopathological manifesttaions co-occurring with anxiety** | **JBI Checklist-total score** |
| Karahan et al., 2016 | Turkey | Cross-sectional and case-control study | Rheumatoid arthritis | Outpatients with rheumatoid arthritis, n=148  healthy subjects, n=100 | BAI | **BDI**  41.9% of outpatients with rheumatoid arthritis had depressive symptoms (BDI score ≥ 17)  Outpatients with rheumatoid arthritis had more severe depressive symptoms than healthy subjects (BDI mean ± SD: 15.57 ± 7.95 vs 9.15 ± 4.23) | **7** |
| Kayhan et al., 2016 | Turkey | Cross-sectional and case-control study | Fibromyalgia | Outpatients with fibromyalgia,  n=96  healthy subjects,  n= 94 | SCID for DSM-IV | **SCID for DSM-IV**  47.9% of outpatients with fibromyalgia had a sexual dysfunction  28.1% of outpatients with fibromyalgia had a mood disorder  26% of outpatients with fibromyalgia had a major depressive disorder  13.5% of outpatients with fibromyalgia had a personality disorder (histrionic personality disorder, 10.4%) | **7** |
| Kokoszka et al., 2016 | Poland | Cross-sectional and observational study | Chronic kidney disease undergoing dialysis | Outpatients with chronic kidney disease undergoing dialysis, n=107 | M.I.N.I. Plus 5.0.0, Polish version | **M.I.N.I. Plus 5.0.0, Polish version**  78.5% of outpatients with chronic kidney disease had depressive disorders (29% had a major depressive episode, 28% had dysthymia, 21.5% had depression with melancholic features;  11.2% had depressive and anxiety disorders) | **7** |
| Lomper et al., 2016 | Poland | Cross-sectional and observational study | Bronchial asthma | Outpatients with controlled asthma, n=33  outpatients with uncontrolled asthma, n=63 | HADS-A | **HADS-D**  Outpatients with uncontrolled asthma did not differ for severity of depressive symptoms from patients with controlled asthma (HADS-D mean ± SD: 10.9 ± 4.8 vs 8.9 ± 4.5, p > 0.05)  Among outpatients with uncontrolled asthma: 28.6% had probable depression (HADS-D score 8-10) and 49.2% had definite depression (HADS-D score 11-21)  Among outpatients with controlled asthma: 33.3% had probable depression (HADS-D score 8.10) and 30.3% had definite depression (HADS-D score 11-21) | **7** |
| Reference | **Country/City** | **Study Design** | **Diagnosis** | **Sample size** | **Instrument to assess anxiety** | **Psychopathological manifesttaions co-occurring with anxiety** | **JBI Checklist-total score** |
| Mielcarek et al., 2016 | Poland | Longitudinal and observational study | Ovarian cancer (during multimodality ovarian cancer treatment) | Outpatients with ovarian cancer,  n=106 | HADS-A | **HADS-D**  Among outpatients with ovarian cancer, the levels of severity of depressive symptoms remained relatively stable across the therapeutic process:  Time 1: HADS-D mean ± SD 5.51 ± 3.00  Time 2: HADS-D mean ± SD 5.62 ± 3.00  Time 3: HADS-D mean ± SD 5.71 ± 3.51  Time 4: HADS-D mean ± SD 5.82 ± 3.51 | **7** |
| Moryś et al., 2016 | Poland | Cross-sectional and observational study | Coronary artery disease | Inpatients with coronary artery disease,  n=120 | HADS-A  GAD-7  STAI | **HRSD, BDI-II, PHQ-9, HADS-D**  31% of inpatients with coronary artery disease had depressive symptoms as assessed by the HRSD (score ≥ 8)  32% of inpatients with coronary artery disease had depressive symptoms as assessed by the BDI-II (score ≥ 14)  37.5% of inpatients with coronary artery disease had depressive symptoms as assessed by the PHQ-9 (score ≥ 5)  7.5% of inpatients with coronary artery disease had depressive symptoms as assessed by the HADS-D (score ≥ 8) | **7** |
| Reference | **Country/City** | **Study Design** | **Diagnosis** | **Sample size** | **Instrument to assess anxiety** | **Psychopathological manifesttaions co-occurring with anxiety** | **JBI Checklist-total score** |
| Olveira et al., 2016 | Spain | Cross-sectional and observational study | Cystic fibrosis | Outpatients with cystic fibrosis,  n=336 | HADS-A | **HADS-D**:  12.2% of outpatients with cystic fibrosis had depressive symptoms (HADS-D score ≥ 8) | **7** |
| Rani Chadalawada et al., 2016 | India | Cross-sectional and observational study | Different medical conditions | Inpatients admitted to surgical ward, n=169 medical Inpatients admitted to medical ward, n=184) | HADS-A | **HADS-D:**  38.5% of inpatients had a borderline depression (HADS-D score 8-10)  38% of inpatients had a possible depression (HADS-D score score>10) | **7** |
| Soósová et al., 2016 | Slovakia | Cross-sectional and observational study | Rheumatoid arthritis | Outpatients with rheumatoid arthritis, n=142 | BAI | **SDS**  31.7% of outpatients with rheumatoid arthritis had minimal to mild depression (SDS score 51-59)  30.3% of outpatients with rheumatoid arthritis had moderate to marked depression (SDS score 60-69)  11.3% of outpatients with rheumatoid arthritis had severe to extremely severe depression (SDS score ≥ 70) | **7** |
| Sun et al., 2016 | China | Cross-sectional and observational study | Type 2 diabetes | Outpatients with type 2 diabetes, n=893 | SAS | **SDS**  43.6% of outpatients with type 2 diabetes had depressive symptoms (SDS score ≥ 50) | **8** |
| Suzuki et al., 2016 | Tokyo | Cross-sectional and observational study | Head/neck cancer | Outpatients with cancer,  n=194 | HADS-A | **HADS-D**  27.3% of outpatients with cancer had depressive symptoms (HADS-D score ≥ 8)  9.3% of outpatients with cancer had severe depressive symptoms (HADS-D score ≥ 11) | **7** |
| Reference | **Country/City** | **Study Design** | **Diagnosis** | **Sample size** | **Instrument to assess anxiety** | **Psychopathological manifesttaions co-occurring with anxiety** | **JBI Checklist-total score** |
| Uchmanowicz et al., 2016 | Poland | Cross-sectional and observational study | Chronic obstructive pulmonary disease | Inpatients with chronic obstructive pulmonary disease, n=102 | HADS-A | **HADS-D**  Inpatients with chronic obstructive pulmonary disease had HADS-D mean ± SD: 10.8 ± 4.14 | **7** |
| Wei et al., 2016 | China | Cross-sectional and observational study | Headache | Outpatients with headache,  n=749 | HADS-A | **HADS-D**  19.7% of outpatients with headache had HADS-D score ≥ 8 | **7** |
| Zebenholzer et al., 2016 | Australia | Cross-sectional and observational study | Episodic/chronic headache | Outpatients with episodic headache, n=232  outpatients with chronic headache, n=160 | HADS-A | **HADS-D**  Outpatients with chronic headache had higher rates of depressive symptoms than those with episodic headache (43.6 % vs 22.8%, p < 0.001) | **8** |
| Zhang et al., 2016 | China | Cross-sectional and observational study | Chronic digestive system diseases | Outpatients and inpatients with chronic digestive system diseases,  n=1,736 | HADS-A | **HADS-D**  31.11% of cases with chronic digestive system diseases had depressive symptoms  20.68% of cases with chronic digestive system diseases had depressive and anxious symptoms | **8** |
| Aggarwal et al., 2017 | India | Cross-sectional and observational study | Chronic kidney disease | Outpatients with chronic kidney disease,  n=200 | HADS-A | **HADS-D**  69% of outpatients with chronic kidney disease had HADS score > 7 | **7** |
| Aggelopoulou et al., 2017 | Athens | Cross-sectional and observational study | Heart failure | Inpatients with heart failure,  n=231 | STAI | **MQ**  96.1% of inpatients with heart failure had depressive symptoms (MQ mean ± SD: 34.3 ± 8.4) | **7** |
| Reference | **Country/City** | **Study Design** | **Diagnosis** | **Sample size** | **Instrument to assess anxiety** | **Psychopathological manifesttaions co-occurring with anxiety** | **JBI Checklist-total score** |
| AlBekairy et al., 2017 | Saudi Arabia | Cross-sectional and observational study | Type 2 diabetes | Inpatients with type 2 diabetes, n=158 | HADS-A | **HADS-D**  53.8% of inpatients with type 2 diabetes had depressive symptoms  31% of inpatients with type 2 diabetes had mild depressive symptoms (HADS-D score 8-10),  17.1% of inpatients with type 2 diabetes had moderate depressive symptoms (HADS-D score 11.14)  5.7% of inpatients with type 2 diabetes had severe depressive symptoms (HADS-D score ≥ 15)  32.9% of inpatients with type 2 diabetes had depressive and anxious symptoms | **7** |
| Azizoddin et al., 2017 | Southern California | Cross-sectional and observational study | Systemic lupus erythematosus | Outpatients with systemic lupus erythematosus,  n=128 | HADS-A | **HADS-D**  23.1% of outpatients with systemic lupus erythematosus had depressive symptoms (HADS-D score ≥ 8) | **7** |
| Baniasadi et al., 2017 | Iran | Cross-sectional and observational study | Irritable bowel syndrome | Outpatients wit irritable bowel syndrome,  n=123 | DASS-21 | **DASS-21**  70.7% of outpatients with irritable bowel syndrome had depressive symptoms | **7** |
| Betancur et al., 2017 | Brazil | Cross-sectional and observational study | HIV | Outpatients with HIV, n=47 | BAI | **BDI**  21.3% of outpatients with HIV had mild depressive symptoms (BDI score: 12-19)  40.4% of outpatients with HIV had moderate depressive symptoms (BDI score 20-35)  19.1% of outpatients with HIV had severe depressive symptoms (BDI score 36-63) | **7** |
| Reference | **Country/City** | **Study Design** | **Diagnosis** | **Sample size** | **Instrument to assess anxiety** | **Psychopathological manifesttaions co-occurring with anxiety** | **JBI Checklist-total score** |
| Byrne et al., 2017 | Canada | Cross-sectional and observational study | Inflammatory bowel disease | Outpatients with inflammatory bowel disease,  n=327 | GAD-7 | **PHQ-9**  25.5% of outpatients with inflammatory bowel disease had depressive symptoms | **7** |
| Cui et al., 2017 | China | Cross-sectional and observational study | Parkinson’s disease | Outpatients with Parkinson’s disease, n=403 | HAM-A | **HAM-D**  11.2% outpatients with Parkinson’s disease had depressive symptoms (HAM-D score ≥ 13) | **8** |
| El Filali et al., 2017 | Morocco | Cross-sectional and observational study | End-stage renal disease undergoing haemodialysis | Outpatients with  end-stage renal disease  undergoing haemodialysis,  n=103 | M.I.N.I. for DSM-IV | **M.I.N.I. for DSM-IV**  34% of outpatients with end-stage  renal disease undergoing haemodialysis had a major depressive disorder (65.7% with melancholic features, 25.7% recurrent depression)  16.5% of outpatients with end-stage renal disease undergoing haemodialysis had thoughts of suicide (1.9% planned their suicide) | **7** |
| Faezi et al., 2017 | Iran | Cross-sectional and observational study | Systemic sclerosis | Outpatients with systemic sclerosis, n=114 | Cattel Anxiety Self-assessment Scale | **BDI**  68.4% of outpatients with systemic sclerosis had depressive symptoms:  22% borderline (BDI score 7-10)  21% mild (BDI score 11-16)  19.3% moderate (BDI score 17-20)  3.5% severe (BDI score 21-30)  2.6% extreme (BDI score 31-40) | **7** |
| Fortier et al., 2017 | Australia | Longitudinal and observational study | Chronic hepatitis C virus | Outpatients with chronic hepatitis C virus,  n=415 | DASS-21 | **DASS-21**  47% of outpatients chronic hepatitis C virus had moderate to extremely severe symptoms of depression (DASS-21 score) | **7** |
| Reference | **Country/City** | **Study Design** | **Diagnosis** | **Sample size** | **Instrument to assess anxiety** | **Psychopathological manifesttaions co-occurring with anxiety** | **JBI Checklist-total score** |
| Lin et al., 2017 | Iran | Cross-sectional and observational study | Epilepsy | Outpatients with epilepsy,  n=999 | HADS-A | **HADS-D**  21.9% of outpatients with epilepsy had depressive symptoms (HADS-D score ≥ 11)  24.9% of outpatients with epilepsy were borderline cases of depression (HADS-D score 8-10) | **8** |
| Magyari et al., 2017 | Hungary | Cross-sectional and observational study | Hodgkin lymphoma | Outpatients with Hodgkin lymphoma, n=140 | HADS-A | **HADS-D**  6% of outpatients with Hodgkin lymphoma were borderline cases of depression (HADS-D score 8–10)  4% of outpatients with Hodgkin lymphoma had severe depressive symptoms (HADS-D score 11-21) | **7** |
| Montserrat-Capdevila et al., 2017 | Spain | Longitudinal and observational study | Chronic obstructive pulmonary disease | Outpatients with chronic obstructive pulmonary disease, n=512 | HADS-A | **HADS-D**  12.5% of outpatients with chronic obstructive pulmonary disease had depressive symptoms (HADS-D score ≥ 10) | **8** |
| Müller-Tasch et al., 2017 | Germany | Cross-sectional and observational study | Chronic heart failure | Outpatients with chronic heart failure, n=308 | PHQ for DSM-IV - Panic disorder and anxiety disorder module | **PHQ for DSM-IV – depression module**  12.1% of outpatients with chronic heart failure had a major depressive disorder  15.1% of outpatients with chronic heart failure other depressive disorders | **7** |
| Nakagawa et al., 2017 | Japan | Cross-sectional and observational study | Chronic foot and ankle disease | Outpatients with chronic foot and ankle disease, n=250  hallux valgus outpatients, n=58 plantar fasciitis outpatients, n=32 ankle osteoarthritis outpatients, n=28 adult flatfoot outpatients, n=22 rheumatoid arthritis outpatients, n=18 | HADS-A | **HADS-D**  27% outpatients with chronic foot and ankle had depressive symptoms (HADS-D score ≥ 8)  12% outpatients with chronic foot and ankle disease had depressive symptoms (HADS-D score >11)  16% of the hallux valgus outpatients, 28% of the plantar fasciitis outpatients, and 33% of the ankle osteoarthritis outpatients had depressive symptoms (HADS-D score ≥ 8) | **7** |
| Reference | **Country/City** | **Study Design** | **Diagnosis** | **Sample size** | **Instrument to assess anxiety** | **Psychopathological manifesttaions co-occurring with anxiety** | **JBI Checklist-total score** |
| On et al., 2017 | Australia | Cross-sectional and case-control study | Gastroesophageal reflux disease | Outpatients with gastroesophageal reflux disease,  n=221  healthy subjects (only men),  n=1391 | GAD-7 | **BDI**  Outpatients with gastroesophageal reflux disease, compared to healthy subjects, had higher rates of past depressive symptoms (6.7% vs 4.6%, p < 0.001) and current depressive symptoms (30.5% vs 14.2%, p < 0.001) | **7** |
| Pfeuffer et al., 2017 | Germany | Cross-sectional and observational study | Pulmonary arterial hypertension,  chronic thromboembolic pulmonary hypertension | Outpatients with pulmonary arterial hypertension,  n=70  chronic thromboembolic pulmonary hypertension outpatients,  n=23 | HADS-A | **HADS-D**  30% of outpatients with pulmonary arterial hypertension and 56.5% of outpatients with chronic thromboembolic pulmonary hypertension had depressive symptoms (HADS-D score ≥8) | **7** |
| Reference | **Country/City** | **Study Design** | **Diagnosis** | **Sample size** | **Instrument to assess anxiety** | **Psychopathological manifesttaions co-occurring with anxiety** | **JBI Checklist-total score** |
| Raikhy et al., 2017 | India | Cross-sectional and observational study | Dermatology diagnosis (i.e., acne vulgaris, psoriasis, lichen planus, vitiligo, urticarial) | Outpatients with dermatological diseases,  n=402 | MSE for ICD-10 | **MSE for ICD-10**  36.32% of dermatological outpatients had a major depression disorder  7.96% of dermatological outpatients had a somatoform disorder  6.47% of dermatological outpatients had an obsessive-compulsive disorder  4.98% of dermatological outpatients had an adjustment disorder  4.98% of dermatological outpatients ha an alcohol dependence  2.99% of dermatological outpatients had schizophrenia  2.99% of dermatological outpatients had a bipolar disorder | **7** |
| Rebollo Rubio et al., 2017 | Spain | Cross-sectional and observational study | Chronic kidney disease | Outpatients with chronic kidney disease,  n=152 | HADS-A | **HADS-D**  27% of outpatients with chronic kidney disease had depressive symptoms | **7** |
| Thapa et al., 2017 | Nepal | Cross-sectional and case-control study | Chronic obstructive pulmonary disease | Outpatients with chronic obstructive pulmonary disease, n=93  healthy subjects, n=105 | BAI | **BDI**  Outpatients with chronic obstructive pulmonary disease had more severe depressive symptoms than healthy subjects (BDI mean ± SD: 27.72 ± 9.37 vs 11.60 ± 8.42, p < 0.05)  Outpatients with chronic obstructive pulmonary disease had a higher rate of severe depression than healthy subjects (36.6% vs 4.8%) | **7** |
| Reference | **Country/City** | **Study Design** | **Diagnosis** | **Sample size** | **Instrument to assess anxiety** | **Psychopathological manifesttaions co-occurring with anxiety** | **JBI Checklist-total score** |
| Watad et al., 2017 | Israel | Cross-sectional and case-control study | Rheumatoid arthritis | Outpatients with rheumatoid arthritis, n=11,782  subjects without rheumatoid arthritis, n=57973 | ICD-9 |  | **8** |
| Zhang et al., 2017 | China | Cross-sectional and case-control study | Rheumatoid arthritis | Outpatients with rheumatoid arthritis, n=160  healthy subjects, n=141 | HADS-A | **HADS-D**  Outpatients with rheumatoid arthritis had higher rates of depressive symptoms than healthy subjects (HADS-D score ≥ 8) (27.5% vs 17.7%, p < 0.01) | **7** |
| Zhu et al., 2017 | The Netherlands | Cross-sectional and observational study | Parkinson's disease | Outpatients with Parkinson's disease, n=409 | HADS-A | **BDI**  Among outpatients with Parkinson's disease those with anxiety had more severe depressive symptoms than those without anxiety (BDI score mean ± SD 18.17 ± 7.25 vs. 8.64 ± 5.22, p < 0.001)  70% of outpatients with Parkinson's disease fulfilled the criteria for depression (BDI ≥ 15) | **7** |
| Al-Atram, 2018 | Saudi Arabia | Cross-sectional and observational study | Medical conditions | Inpatients,  n=400 | M.I.N.I. Plus for DSM-IV  HADS-A | **M.I.N.I. Plus for DSM-IV**  14% of inpatients had a depressive disorder  4% of inpatients had an adjustment disorder  1% of inpatients had a mixed anxiety and depression disorder | **7** |
| Reference | **Country/City** | **Study Design** | **Diagnosis** | **Sample size** | **Instrument to assess anxiety** | **Psychopathological manifesttaions co-occurring with anxiety** | **JBI Checklist-total score** |
| Al-Rawashdeh et al., 2018 | Jordan | Cross-sectional and observational study | Otolaryngology | Otolaryngology outpatients,  n=1,328 | GAD-7 | **PHQ-9**  36.1% of otolaryngology outpatients had depressive symptoms: 22.6% had moderate symptoms (PHQ-9 score 10-14), 10.1% had moderate to severe symptoms (PHQ-9 score 15-19), 3.4% had severe symptoms (PHQ-9 score≥20) | **8** |
| Beesley et al., 2018 | Australia | Cross-sectional and observational study | Neuroendocrine tumours | Outpatients with neuroendocrine tumours,  n=111 | HADS-A | **HADS-D**  9% of outpatients with neuroendocrine tumours had clinical depression (HADS-D score > 11);  11% of outpatients with neuroendocrine tumours had borderline depression (HADS-D score 8-10) | **7** |
| Cui et al., 2018 | China | Cross-sectional and case-control study | Primary Sjögren’s syndrome | Outpatients with primary Sjögren’s syndrome,  n=160  healthy subjects, n=170 | HADS-A | **HADS-D**  Outpatients with primary Sjögren’s syndrome had more severe depressive symptoms than healthy subjects (HADS-D mean ± SD: 6.71 ± 4.32 vs 3.87 ± 2.80, p < 0.001)  36.9% of outpatients with primary Sjögren’s syndrome had depression | **7** |
| Czyżewski et al., 2018 | Warsaw | Cross-sectional and observational study | End-stage renal disease post kidney transplantation | Outpatients with end-stage renal disease post kidney transplantation,  n=118 | DASS-42 | **DASS-42**  The DASS-42 was 11.9±11.2, indicating mild depression symptoms (DASS-42 score 10-13) | **7** |
| Reference | **Country/City** | **Study Design** | **Diagnosis** | **Sample size** | **Instrument to assess anxiety** | **Psychopathological manifesttaions co-occurring with anxiety** | **JBI Checklist-total score** |
| Filippakis et al., 2018 | USA | Cross-sectional and observational study | Benign fasciculation syndrome | Outpatients with benign fasciculation syndrome,  n=35 | SAS |  | **7** |
| Fotos et al., 2018 | Athens | Cross-sectional and case-control study | Hepatitis B virus,  hepatitis C virus | Outpatients with hepatitis,  n=111  healthy subjects, n=111 | STAI | **BDI**  Outpatients with hepatitis had more severe depressive symptoms than healthy subjects (BDI mean ± SD: 9.86 ± 5.24 vs 6.01 ± 3.12, p < 0.001) | **7** |
| Gao et al., 2018 | China | Cross-sectional and case-control study | Bronchiectasis | Outpatients with bronchiectasis,  n=163  healthy subjects,  n=80 | HADS-A | **HADS-D**  Outpatients with bronchiectasis had more severe depressive symptoms than healthy subjects (HADS-D mean ± SD: 5.31 ± 3.62 vs 3.51 ± 2.52, p < 0.001)  Outpatients with bronchiectasis had a higher rate of depression than healthy subjects (30.1% vs 10%)  22.1% of outpatients with bronchiectasis had anxiety and depression symptoms | **7** |
| Guzelhan et al., 2018 | Turkey | Longitudinal and observational study | Coronary artery bypass graft | Coronary artery bypass graft outpatients,  n=137 | STAI |  | **7** |
| Reference | **Country/City** | **Study Design** | **Diagnosis** | **Sample size** | **Instrument to assess anxiety** | **Psychopathological manifesttaions co-occurring with anxiety** | **JBI Checklist-total score** |
| Ho et al., 2018 | Singapore | Cross-sectional and observational study | Obesity | Outpatients with obesity,  n=83 | HADS-A | **HADS-D**  18% of outpatients with obesity had depressive symptoms (HADS-D score > 10)  11% of outpatients with obesity and borderline depression (HADS score 8-10) | **7** |
| Kapısız et al., 2018 | Turkey | Cross-sectional and observational study | Chronic obstructive pulmonary disease | Inpatients with chronic obstructive pulmonary disease, n=90 | HADS-A | **HADS-D**  56.7% of inpatients with chronic obstructive pulmonary disease had depressive symptoms (HADS score 8-21) | **8** |
| Kwan et al., 2018 | Malaysia | Cross-sectional and observational study | Psoriasis | Outpatients with psoriasis,  n=100 | DASS-42 | **DASS-42**  27% of outpatients with psoriasis had depression:  7% mild symptoms (DASS-42 score 10-13)  13% moderate symptoms (DASS-42 score 14-20)  4% severe symptoms (DASS-42 score 21-27)  3% extremely severe symptoms (DASS-42 score ≥ 28) | **7** |
| Lewis et al., 2018 | Manchester | Cross-sectional and observational study | Gastroentero-pancreatic neuroendocrine tumours | Outpatients with gastroentero-pancreatic neuroendocrine tumours,  n=50 | HADS-A | **HADS-D**  12% of outpatients with gastroenterop-ancreatic neuroendocrine tumours had depressive symptoms (HADS score ≥ 8) | **7** |
| Milligan et al., 2018 | Qatar | Cross-sectional and observational study | Cancer | Outpatients with cancer,  n=57 | HADS-A | **HADS-D**  19.3% of outpatients with cancer had mild depressive symptoms  19.3% of outpatients with cancer had moderate depressive symptoms  5.3% of outpatients with cancer had severe depressive symptoms | **7** |
| Reference | **Country/City** | **Study Design** | **Diagnosis** | **Sample size** | **Instrument to assess anxiety** | **Psychopathological manifesttaions co-occurring with anxiety** | **JBI Checklist-total score** |
| Navabi et al., 2018 | Pennsylvania | Cross-sectional and observational study | Inflammatory bowel disease | Outpatients with inflammatory bowel disease,  n=432 | HADS-A | **HADS-D**  25% of outpatients with inflammatory bowel disease had depressive symptoms (HADS-D score 8)  19.9% of outpatients with inflammatory bowel disease had depressive and anxious symptoms | **8** |
| Nowicka-Sauer et al., 2018 | Poland | Cross-sectional and observational study | Systemic lupus erythematosus | Outpatients with systemic lupus erythematosus,  n=80 | STAI | **BDI**  Systemic lupus erythematosus outpatients had mild depression (BDI mean score 13.24) | **7** |
| Olsen et al., 2018 | Norwey | Longitudinal and case-control study | Coronary heart disease | Outpatients with coronary heart disease,  n=183  subjects without coronary heart disease,  n=592 | HADS-A | **HADS-D**  19% of outpatients with coronary heart disease had depressive symptoms (score ≥ 8)  Outpatients with coronary heart disease, compared to subjects without coronary heart, had more severe depressive symptoms at baseline (4.69 ± 4.10 vs. 3.84 ± 3.45, p < 0.001) and at 3 years after percutaneous coronary intervention (3.78 ± 4.09 vs. 3.08 ± 3.33, p < 0.001) | **8** |
| Park et al., 2018 | USA | Cross-sectional and observational study | Breast cancer | Outpatients with breast cancer,  n=54 | HADS-A | **HADS-D**  11% of outpatients with breast cancer had mild depressive symptoms (HADS-D score 8-10)  9% of outpatients with breast cancer had moderate to severe depressive symptoms (HADS-D score ≥ 11) | **7** |
| Reference | **Country/City** | **Study Design** | **Diagnosis** | **Sample size** | **Instrument to assess anxiety** | **Psychopathological manifesttaions co-occurring with anxiety** | **JBI Checklist-total score** |
| Polikandrioti et al., 2018 | Greece | Cross-sectional and observational study | Atrial ﬁbrillation | Outpatients with atrial ﬁbrillation,  n=170 | HADS-A | **HADS-D**  20.2% of outpatients with atrial ﬁbrillation had severe anxious and depressive symptom (HADS-D score ≥ 11) | **7** |
| Qin et al., 2018 | China | Cross-sectional and observational study | HIV | Outpatients with HIV (pregnant women), n=194 | HADS-A | **HADS-D**  54.1% of pregnant women with HIV had depressive symptoms (HADS-D score ≥ 8) | **7** |
| Rahim et al., 2018 | Malaysia | Cross-sectional and observational study | Rheumatoid arthritis | Outpatients with rheumatoid arthritis, n=189 | DASS-21 | **DASS-21**  23.3% of outpatients with rheumatoid arthritis had depressive symptoms | **7** |
| Rezapour et al., 2018 | Germany | Cross-sectional and case-control study | Glaucoma | Patients with glaucoma,  n=293  subjects without glaucoma,  n=14,657 | GAD-7 | **PHQ-9**  Patients with glaucoma and subjects without glaucoma had similar rates of depressive symptoms (6.6% vs 7.7%, p > 0.05) | **8** |
| Semaan et al., 2018 | Lebanon | Cross-sectional and observational study | End-stage renal disease | Outpatients with end-stage renal disease, n=83 | HADS-A | **HADS-D**  40.8% of outpatients with end-stage renal disease had probable depression (HADS-D score 7-10)  24.1% of outpatients with end-stage renal disease had anxious and depressive symptoms | **7** |
| Reference | **Country/City** | **Study Design** | **Diagnosis** | **Sample size** | **Instrument to assess anxiety** | **Psychopathological manifesttaions co-occurring with anxiety** | **JBI Checklist-total score** |
| Tian et al., 2018 | China | Cross-sectional and observational study | Psoriasis | Outpatients with psoriasis,  n=208 | GAD-7 | **PHQ-9**  13.9% of outpatients with psoriasis had moderate to severe depressive symptoms (PHQ-9 score ≥ 10) | **7** |
| Tomazoni et al., 2018 | Brazil | Cross-sectional survey and observational study | Crohn’s disease | Outpatients with Crohn’s disease, n=110 | HADS-A | **HADS-D**  7.3% of outpatients with Crohn’s disease had depressive symptoms  (HADS-D score ≥ 9) | **7** |
| Xiao et al., 2018 | China | Cross-sectional and observational study | Chronic obstructive pulmonary disease | Outpatients with chronic obstructive pulmonary disease, n=275 | HADS-A | **HADS-D**  13.1% of outpatients with chronic obstructive pulmonary disease had depressive symptoms (HADS-D score ≥ 8) | **7** |
| Zamora-Racaza et al., 2018 | Philippines  Southern California | Cross-sectional and observational study | Systemic lupus erythematosus | Outpatients with systemic lupus erythematosus living in California,  n=135  outpatients with systemic lupus erythematosus living in Philippines,  n=100 | HADS-A | **HADS-D**  Outpatients with systemic lupus erythematosus living in California had higher rates of depressive symptoms than those living in Philippines (48.9% vs 27%, p < 0.001) | **7** |
| Allabadi et al., 2019 | Palestine | Cross-sectional and observational study | Cardiovascular diseases | Inpatients with cardiovascular diseases,  n=1022 | DASS-42 | **DASS-42**  52.9% of inpatients with cardiovascular diseases had depressive symptoms: 33.4% had mild to moderate symptoms (DASS-42 score 10-20)  19.5% had severe to very severe symptoms (DASS-42 score ≥ 21) | **8** |
| Reference | **Country/City** | **Study Design** | **Diagnosis** | **Sample size** | **Instrument to assess anxiety** | **Psychopathological manifesttaions co-occurring with anxiety** | **JBI Checklist-total score** |
| Been et al., 2019 | Rotterdam | Cross-sectional and observational study | HIV | Outpatients with HIV, n=306 | HADS-A | **HADS-D**  15% of HIV outpatients had mild depressive symptoms  (HADS-D score 8-10)  12.7% of HIV outpatients had moderate to severe depressive symptoms  (HADS-D score ≥ 11) | **7** |
| Brito et al., 2019 | Brazil | Cross-sectional and observational study | End-stage renal disease | Outpatients undergoing dialysis, n=130  transplant outpatients, n=75 | BAI | **BDI**  31.2% of outpatients with end-stage renal disease had depressive symptoms  (BDI score ≥ 11)  41.7% of outpatients undergoing dialysis had depressive symptoms (BDI score ≥ 11)  13.3% of transplant outpatients had depressive symptoms (BDI score ≥ 11) | **7** |
| Bucourt et al., 2019 | France | Cross-sectional and observational study | Rheumatic diseases | Outpatients with fibromyalgia,  n=48  outpatients with rheumatoid arthritis, n=47  outpatients with spondyloarthritis, n=47  outpatients with Sjogren’s syndrome, n=23 | M.I.N.I. for DSM-IV  GHQ | **M.I.N.I. for DSM-IV, VAS, FSS, GHQ**  Outpatients with fibromyalgia, compared to outpatients with rheumatoid arthritis, had higher levels of pain (VAS mean: 6.96 vs 5.26, p < 0.05), fatigue (FSS mean: 6.13 vs 4.47, p < 0.05), depression (GHQ mean: 6.13 vs 3.74, p < 0.05)  Outpatients with fibromyalgia, compared to outpatients with rheumatoid arthritis, had higher rates of current major depressive episodes (47.9% vs 21.3%, p < 0.05) and recurrent major depressive episode (70.8% vs 38.3%, p < 0.05)  Outpatients with fibromyalgia, compared to outpatients with spondyloarthritis, had higher rates of recurrent major depressive episode (70.8% vs 23.4%, p < 0.05)  Outpatients with fibromyalgia, compared to outpatients with Sjogren’s syndrome, had higher rates of recurrent major depressive episode (70.8% vs 21.7%, p < 0.05) | **7** |
| Reference | **Country/City** | **Study Design** | **Diagnosis** | **Sample size** | **Instrument to assess anxiety** | **Psychopathological manifesttaions co-occurring with anxiety** | **JBI Checklist-total score** |
| Ding et al., 2019 | China | Cross-sectional and case-control study | Acute myeloid leukaemia | Outpatients with acute myeloid leukaemia, n=208  healthy subjects, n=200 | HADS-A | **HADS-D**  Outpatients with acute myeloid leukaemia had higher rates of depressive symptoms than healthy subjects (27.9% vs 10%, p < 0.001) (HADS-D score ≥ 8)  Outpatients with acute myeloid leukaemia had more severe depressive symptoms than healthy subjects (HADS-D mean ± SD: 6.61 ± 3.00 vs 4.22 ± 2.51, p < 0.001) | **7** |
| Fattouh et al., 2019 | Lebanon | Cross-sectional and observational study | Chronic diseases | Inpatients with chronic diseases, n=150 | HADS-A | **HADS-D**  29.3% of inpatients with chronic diseases had a borderline depression (HADS-D score ≥ 8)  21.3% of inpatients with chronic disease had a clinically relevant depression (HADS-D score ≥ 11) | **7** |
| Ganasegeran et al., 2019 | Malaysia | Cross-sectional and observational study | Chronic pain | Outpatients with chronic pain, n=117 | DASS-21 | **DASS-21**  44.4% of outpatients with chronic pain had depression symptoms (DASS-21 score ≥ 10) | **7** |
| Reference | **Country/City** | **Study Design** | **Diagnosis** | **Sample size** | **Instrument to assess anxiety** | **Psychopathological manifesttaions co-occurring with anxiety** | **JBI Checklist-total score** |
| Geue et al., 2019 | Germany | Longitudinal and observational study | Cancer | Outpatients with cancer, n=514 | HADS-A | **HADS-D**  16.9% of outpatients with cancer had elevated levels of depression (HADS-D score ≥ 8) at T1  17.7% of outpatients with cancer  had elevated levels of depression (HADS-D score ≥ 8) at T2  14% of outpatients with cancer  had elevated levels of depression and anxiety (HADS-D score ≥ 8) at T1  15% of outpatients with cancer  had elevated levels of depression and anxiety (HADS-D score ≥ 8) at T2 | **7** |
| Hampton et al., 2019 | Texas | Cross-sectional and observational study | Hip pathology | Outpatients with femoro-acetabular impingement,  n= 55  outpatients with lateral trochanteric pain syndrome,  n=23  outpatients with hip osteoarthritis,  n=184  outpatients with vascular necrosis of the hip,  n=16 | HADS-A | **HADS-D** (HADS-D score 11-21)  7.3% of outpatients with femoro-acetabular impingement had pathological levels of depressive symptoms  4.3% of outpatients with lateral trochanteric pain syndrome had pathological levels of depressive symptoms  14.1% of outpatients with lateral trochanteric pain syndrome hip had pathological levels of depressive symptoms  6.3% of outpatients with vascular necrosis of the hip had pathological levels of depressive symptoms | **7** |
| Reference | **Country/City** | **Study Design** | **Diagnosis** | **Sample size** | **Instrument to assess anxiety** | **Psychopathological manifesttaions co-occurring with anxiety** | **JBI Checklist-total score** |
| Henry et al., 2019 | France | Cross-sectional and observational study | Multiple sclerosis | Outpatients with multiple sclerosis, n=110 | HADS-A | **HADS-D**  21% of outpatients with multiple sclerosis had possible depressive symptoms (HADS-D score 8-10)  17% of outpatients with multiple sclerosis had probable depressive symptoms (HADS-D score > 10) | **7** |
| Karlıdağ et al., 2019 | Turkey | Cross-sectional and case-control study | Chronic hepatitis B virus | Outpatients with chronic hepatitis B treated with oral antiviral,  n=103  outpatients with untreated chronic infection,  n=94  healthy subjects,  n=50 | HAM-A | **HAM-D**  Outpatients with chronic hepatitis B and outpatients with chronic infection had more severe depressive symptom than healthy subjects (HAM-D mean ± SD: 8.46 ± 5.44 vs 5.28 ± 4.16 vs 5.16 ± 3.51, p < 0.001) | **7** |
| Khan et al., 2019 | Pakistan | Cross-sectional and observational study | Diabetes | Inpatients with diabetes, n=142 | HADS-A | **HADS-D**  49.2% of inpatients with diabetes had depressive symptoms (HADS-D score > 10):  22.8% mild symptoms, 41.4% moderate symptoms, 35.7% severe symptoms | **7** |
| Łabuz-Roszak et al., 2019 | Poland | Cross-sectional and observational study | Multiple sclerosis | Outpatients with multiple sclerosis with pain, n=117  outpatients with multiple sclerosis without pain, n=27) | HADS-A | **HADS-D**  Outpatients with multiple sclerosis with pain had more severe depressive symptoms than those without pain (HADS-D mean ± SD: 6.00 ± 4.20 vs 3.40 ± 3.71, p < 0.05) | **7** |
| Reference | **Country/City** | **Study Design** | **Diagnosis** | **Sample size** | **Instrument to assess anxiety** | **Psychopathological manifesttaions co-occurring with anxiety** | **JBI Checklist-total score** |
| Larice et al., 2019 | Italy | Cross-sectional and observational study | Rheumatoid arthritis | Outpatients with rheumatoid arthritis, n=108 | HADS-A | **HADS-D**  34.3% of outpatient with rheumatoid arthritis had depressive symptoms (HADS-D score ≥ 8) | **7** |
| Ramsenthaler et al., 2019 | UK | Longitudinal and observational study | Multiple myeloma | Inpatients/ outpatients with multiple myeloma, n=238 | HADS-A |  | **7** |
| Schouten et al., 2019 | The Netherlands | Longitudinal and observational study | End-stage renal disease receiving dialysis | Outpatients with end-stage renal disease undergoing dialysis, n=687 | BAI | **BDI**  42% of outpatients with end-stage renal disease outpatients undergoing dialysis had depressive symptoms (BDI score ≥ 13)  18% of outpatients with end-stage renal disease outpatients undergoing dialysis had comorbid depressive and anxious symptoms | **8** |
| Tat et al., 2019 | Turkey | Cross-sectional and case-control study | Chronic urticarial | Outpatients with chronic urticarial,  n=50  healthy subjects,  n=60 | HADS-A | **HADS-D**  48% of outpatients with chronic urticarial had depressive symptoms  (HADS-D score ≥7) | **7** |
| Truong et al., 2019 | Vietnam | Cross-sectional and observational study | Cancer | inpatients with cancer, n=510 | HADS-A |  | **7** |
| Reference | **Country/City** | **Study Design** | **Diagnosis** | **Sample size** | **Instrument to assess anxiety** | **Psychopathological manifesttaions co-occurring with anxiety** | **JBI Checklist-total score** |
| Uhlenbusch et al., 2019 | Germany | Cross-sectional online and observational study | Different rare diseases (e.g., neurofibromatosis, primary biliary cholangitis, autoimmune hepatitis, pulmonary arterial hypertension, Marfan syndrome and primary sclerosing cholangitis) | Outpatients with rare diseases,  n=300 | GAD-7 | **PHQ-9**  42% of the outpatients had moderate to severe e depressive symptoms (PHQ-9 score ≥ 10)  Females had more severe depressive symptoms than males (PHQ-9 mean ± SD: 9.72 ± 6.12 vs 6.33 ± 4.34, Cohens d = 0.78) | **7** |
| Unseld et al., 2019 | Vienna | Cross-sectional and observational study | Cancer | Outpatients with cancer,  n=1017 | HADS-A | **HADS-D, PTSS‐10**  14.4% of outpatients with cancer had severe depressive symptoms (HADS-D score 8-11)  13.2% of outpatients with cancer had depression (HADS-D score >11)  31.7% of outpatients with cancer had post-traumatic stress disorder symptoms (PTSS‐10 score ≥ 12.5)  Female outpatients had higher rates of post-traumatic stress disorder than males (38.9% vs 24.5%, p < 0.001) | **8** |
| Wallis et al., 2019 | The Netherlands | Cross-sectional and observational study | Multiple sclerosis | Outpatients with multiple sclerosis, n=119 | HADS-A | **HADS-D**  37.8% of outpatients with multiple sclerosis had depressive symptoms (HASD-D score ≥ 8) | **7** |
| Reference | **Country/City** | **Study Design** | **Diagnosis** | **Sample size** | **Instrument to assess anxiety** | **Psychopathological manifesttaions co-occurring with anxiety** | **JBI Checklist-total score** |
| Yan et al., 2019 | China | Cross-sectional and observational study | Lung cancer | Outpatients with lung cancer,  n=315 | HADS-A | **HADS-D**  57.1% of outpatients with lung cancer had depressive symptoms  (HASD-D score ≥ 8) | **7** |
| Banerji et al., 2020 | USA | Cross-sectional and observational study | Hereditary angioedema | Outpatients with hereditary angioedema,  n=445 | HADS-A | **HADS-D**  24% of outpatients with hereditary angioedema had depressive symptoms (HASD-D score ≥ 15) | **7** |
| Bekir et al., 2020 | Turkey | Cross-sectional and observational study | Non-cystic fibrosis bronchiectasis | Outpatients with non-cystic fibrosis bronchiectasis,  n=90 | HADS-A | **HADS-D**  41% of outpatients with non-cystic fibrosis bronchiectasis had depressive symptoms (HASD-D score ≥ 7) | **7** |
| Beşirli et al., 2020 | Turkey | Cross-sectional and observational study | Rheumatoid arthritis | Outpatients with rheumatoid arthritis, n=50 | HADS-A | **HADS-D**  54% outpatients with rheumatoid arthritis had depressive symptoms (HASD-D score ≥ 8) | **7** |
| Bove et al., 2020 | Denmark | Cross-sectional and observational study | Chronic obstructive pulmonary disease | Outpatients with chronic obstructive pulmonary disease, n=242 | HADS-A | **HADS-D**  32% of outpatients with chronic obstructive pulmonary disease had depressive symptoms (HASD-D score ≥ 8)  13% of outpatients with chronic obstructive pulmonary disease had depressive and anxious symptoms  (HASD-D score ≥ 8) | **7** |
| Bulat et al., 2020 | Croatia | Cross-sectional and observational study | Psoriasis | Outpatients with psoriasis,  n=51 | STAI | **BDI**  Outpatients with psoriasis had moderate levels of depression (BDI mean score ± SD: 18.52 ± 7.635) | **7** |
| Reference | **Country/City** | **Study Design** | **Diagnosis** | **Sample size** | **Instrument to assess anxiety** | **Psychopathological manifesttaions co-occurring with anxiety** | **JBI Checklist-total score** |
| Camara et al., 2020 | Guinea | Cross-sectional and observational study | HIV | Outpatients with HIV, n=160 | HADS-A | **HADS-D**  16.9% of outpatients with HIV had depressive symptoms (HADS-D score ≥ 8)  8.1% of outpatients with HIV had depressive and anxious symptoms | **7** |
| Chi et al., 2020 | China | Cross-sectional and case-control study | Still’s disease | Outpatients with Still’s disease,  n=82  healthy controls,  n=82 | HADS-A | **HADS-D**  Outpatients with Still’s disease had more severe depressive symptoms than healthy controls (HADS-D mean ± SD: 5.98 ± 4.01 vs 3.37 ± 2.18, p < 0.001) | **7** |
| Dong et al., 2020 | China | Cross-sectional and observational study | Type 2 diabetes | Outpatients with type 2 diabetes, n=997 | GAD-7 | **PHQ-9**  Among outpatients with type 2 diabetes, those with good quality of life had lower rates of depressive symptoms than those with poor quality of life (26.3% vs 43.6%, p < 0.001) | **9** |
| Doser et al., 2020 | Copenhagen | Cross-sectional and observational study | Neurofibromatosis type 1 | Outpatients with neurofibromatosis type 1,  n=244 | GAD-7 | **PHQ-9**  26% of outpatients with neurofibromatosis type 1 had mild depressive symptoms (PHQ-9 score 5-9)  12% of outpatients with neurofibromatosis type 1 had moderate depressive symptoms (PHQ-9 score 10-14)  7% of outpatients with neurofibromatosis type 1 had severe depressive symptoms (PHQ-9 score 20-27) | **7** |
| Reference | **Country/City** | **Study Design** | **Diagnosis** | **Sample size** | **Instrument to assess anxiety** | **Psychopathological manifesttaions co-occurring with anxiety** | **JBI Checklist-total score** |
| Esser et al., 2020 | Germany | Cross-sectional and observational study | Prostate cancer | Inpatients/ outpatients with prostate cancer, n=636 | GAD-7, German version | **PHQ-9**  23% of inpatients/outpatients with prostate cancer had depressive symptoms (PHQ-9 score ≥ 7) | **8** |
| Hamidizadeh et al., 2020 | Iran | Cross-sectional and case-control study | Vitiligo | Outpatients with vitiligo, n=100  healthy subjects, n=100 | BAI | **BDI**  Among outpatients with vitiligo, 16% had moderate depressive symptoms and 12% had severe depressive symptoms  Among healthy subjects 9% had moderate depressive symptoms and 6% had severe depressive symptoms.  (p > 0.05) | **7** |
| Hanna et al., 2020 | New Jersey | Longitudinal and observational study | Multiple sclerosis | Outpatients with multiple sclerosis, n=183 | STAI | **CMDI**  24% of outpatients with multiple sclerosis had depressive symptoms  (CMDI score ≥ 22) | **8** |
| Hao et al., 2020 | China | Cross-sectional and case-control study | COVID-19 | Inpatients with COVID-19,  n=10  inpatients with psychiatric disorders, n=10  healthy subjects, n=10 | DASS-21 | **DASS-21**  COVID-19 inpatients and psychiatry inpatients had more severe depressive symptoms than healthy subjects (DASS-21 mean ± SD: 7.01 ± 6.00 vs 7.82 ± 10.33 vs 1.00 ± 1.41) (p > 0.05) | **7** |
| Jia et al., 2020 | China | Cross-sectional and observational study | Cubital tunnel syndrome | Cubital tunnel syndrome outpatients, n=246 | HADS-A | **HADS-D**  17.8% of outpatients with cubital tunnel syndrome had depressive symptoms (HADS-D score ≥ 8) | **7** |
| Reference | **Country/City** | **Study Design** | **Diagnosis** | **Sample size** | **Instrument to assess anxiety** | **Psychopathological manifesttaions co-occurring with anxiety** | **JBI Checklist-total score** |
| Li et al., 2020a | China | Cross-sectional and observational study | Chronic hepatitis B | Inpatients/ outpatients with chronic hepatitis B,  n=401 | BAI | **BDI**  Among inpatients and outpatients with chronic hepatitis B the BDI mean score was 9.12 ± 9.28 | **7** |
| Li et al., 2020b | China | Cross-sectional and observational study | Incidental pulmonary nodule lesions | Outpatients with incidental pulmonary nodule lesions,  n=201 | HADS-A | **HADS-D**  19.4% of outpatients with incidental pulmonary nodule lesions had depressive symptoms (HADS-D score ≥ 8) | **7** |
| Lima et al., 2020 | Brazil | Cross-sectional and observational study | Chronic obstructive pulmonary disease | Inpatients with chronic obstructive pulmonary disease, n=70 | BAI | **BDI**  74.3% of inpatients with chronic obstructive pulmonary disease had moderate depressive symptoms  (BDI score 20-35)  24.3% of inpatients with chronic obstructive pulmonary disease had severe depressive symptoms  (BDI score 36-63) | **7** |
| Liu et al., 2020 | China | Cross-sectional and observational study | COVID-19 | Inpatients with COVID-19,  n=675 | GAD-7 | **PHQ-9**  19% of COVID-19 inpatients had moderate to severe depressive symptoms (PHQ-9 score 10-20) | **9** |
| Lukaviciute et al., 2020 | Lithuania | Cross-sectional and case-control study | Facial dermatoses (acne, rosacea, perioral dermatitis, folliculitis) | Outpatients with facial dermatoses,  n=543  healthy subjects, n=497 | HADS-A | **HADS-D**  Outpatients with facial dermatoses had higher rates of depressive symptoms than healthy subjects (21.7% vs 6.8%, p < 0.05) (HADS-D score ≥ 8) | **8** |
| Karimi et al., 2020 | Iran | Cross-sectional and observational study | Multiple sclerosis | Outpatients with multiple sclerosis, n=87 | DASS-21 | **DASS-21**  47.1% of outpatients with multiple sclerosis had moderate depression level (DASS-21 score 10-13)  24.1% of outpatients with multiple sclerosis had severe depression level (DASS-21 score 14-20) | **7** |
| Reference | **Country/City** | **Study Design** | **Diagnosis** | **Sample size** | **Instrument to assess anxiety** | **Psychopathological manifesttaions co-occurring with anxiety** | **JBI Checklist-total score** |
| Ma et al., 2020 | China | Cross-sectional and case-control study | Nasal septal deviation,  ear nose and throat problems | Outpatients with nasal septal deviation,  n=76  outpatients with ear nose and throat problems,  n=79 | SAS | **SDS**  Outpatients with nasal septal deviation had higher rates of depressive symptoms than outpatients with ear nose and throat problems (39.5% vs 22.8%, p < 0.05)  (SDS score ˃ 53) | **7** |
| Mani et al., 2020 | Belgium | Cross-sectional and observational study | Bladder cancer | Outpatients with bladder cancer,  n=101 | HADS-A | **HADS-D**  25.3% of outpatients with bladder cancer had depressive symptoms  (HADS-D score ˃ 8) | **7** |
| Mosleh et al., 2020 | Saudi Arabia | Cross-sectional and observational study | Chronic kidney disease | Outpatients with chronic kidney disease,  n=122 | HADS-A | **HADS-D**  27% of outpatients with chronic kidney disease had borderline depression  24.6% of outpatients with chronic kidney had depressive symptoms | **7** |
| Nie et al., 2020 | China | Cross-sectional and observational study | COVID-19 | Inpatients  with COVID-19,  n=78 | SAS | **SDS**  35.9% of inpatients with COVID-19 had depressive symptoms:  20.5% had mild symptoms (SDS score 0.5 – 0.59)  12.8% had moderate symptoms (SDS score 0.6 – 0.69)  2.6% had severe symptoms (SDS score ≥ 0.7) | **8** |
| Reference | **Country/City** | **Study Design** | **Diagnosis** | **Sample size** | **Instrument to assess anxiety** | **Psychopathological manifesttaions co-occurring with anxiety** | **JBI Checklist-total score** |
| Onwubiko et al., 2020 | Nigeria | Cross-sectional and observational study | Glaucoma | Inpatients/  outpatients with glaucoma,  n=182 | HADS-A | **HADS-D**  41.8% of inpatients/outpatients with glaucoma had depressive symptoms (HADS-D score ≥ 8) | **7** |
| Robbertz et al., 2020 | USA - Ohio | Cross-sectional and observational study | Chronic lymphocytic leukaemia | Outpatients with chronic lymphocytic leukaemia,  n=106 | GAD-7 | **PHQ-9**  14% of outpatients with chronic lymphocytic leukaemia had depressive symptoms (PHQ-9 score ≥ 15) | **7** |
| Treudler et al., 2020 | Germany | Cross-sectional and case-control study | Atopic eczema | Outpatients with atopic eczema,  n=372  healthy subjects, n=9,109 | GAD-7 | **CES-D**  Outpatients with atopic eczema had higher rates of depressive symptoms than healthy subjects (9.3% vs 6.3%, p < 0.001) (CES-D score ≥ 23) | **7** |
| Van den Brekel et al., 2020 | The Netherlands | Cross-sectional and observational study | Cancer | Inpatients  with cancer,  n=2144 | Utrecht Symptom Diary |  | **8** |
| Vikjord et al., 2020 | Norway | Longitudinal and observational study | Chronic obstructive pulmonary disease | Outpatients with chronic obstructive pulmonary disease, n=2076 | HADS-A | **HADS-D**  15.9% of outpatients with chronic obstructive pulmonary disease had depressive symptom (score ≥ 8)  10% of outpatients with chronic obstructive pulmonary disease had anxious and depressive symptoms | **8** |
| Vojvodić et al., 2020 | Serbia | Cross-sectional and observational study | Melanoma | Inpatients  with melanoma,  n=40 | BAI | **BDI**  Inpatients with melanoma reported more severe depressive symptoms in stage IV (with distant metastases) than in stages I and II (localized disease) (BDI mean: 6 vs 2.5, p < 0.05) | **6** |
| Reference | **Country/City** | **Study Design** | **Diagnosis** | **Sample size** | **Instrument to assess anxiety** | **Psychopathological manifesttaions co-occurring with anxiety** | **JBI Checklist-total score** |
| Williamson et al., 2020 | New York | Cross-sectional and observational study | Lung cancer | Outpatients  with lung cancer,  n=50 | HADS-A | **HADS-D**  4% of outpatients with lung cancer had depressive symptoms (HADS-D score ≥ 11) | **8** |
| Woon et al., 2020 | Malaysia | Cross-sectional and observational study | Type 1 or type 2 diabetes | Outpatients with diabetes, n=300 | GAD-7 | **BDI-II**  20% of outpatients with diabetes had depressive symptoms (BDI-II score ≥ 10) | **9** |
| Yon et al., 2020 | Turkey | Cross-sectional and case-control study | Chronic migraine | Outpatients with chronic migraine, n=21  healthy subjects,  n=21 | HAM-A | **BDI**  Outpatients with chronic migraine, compared to healthy subjects, had higher rates of mild depressive symptoms (65% vs 35%; BDI score: 14-19), comparable rates of moderate depressive symptoms (50% vs 50%; BDI score 20-28), higher rates of severe depressive symptoms (100% vs 0%; BDI score 29-63) (p < 0.05) | **7** |
| Yuan et al., 2020 | China | Cross-sectional and observational study | Oral cancer | Inpatients with oral cancer,  n=230 | SAS | **CES-D**  65.21% of inpatients with oral cancer had depressive symptoms (CES-D score ≥ 16) | **8** |
| Zhang et al., 2020 | China | Cross-sectional and observational study | COVID-19 | Inpatients  with COVID-19,  n=296 | HADS-A | **HADS-D**  18.6% of COVID-19 inpatients had depressive symptoms (HADS-D score ≥ 11) | **8** |
| An et al., 2021 | China | Cross-sectional and observational study | Chronic obstructive pulmonary disease | Outpatients with chronic obstructive pulmonary disease, n=86 | HADS-A | **HADS-D**  38.4% of outpatients with chronic obstructive pulmonary disease had depressive symptoms (HADS-D score ˃ 8) | **7** |
| Reference | **Country/City** | **Study Design** | **Diagnosis** | **Sample size** | **Instrument to assess anxiety** | **Psychopathological manifesttaions co-occurring with anxiety** | **JBI Checklist-total score** |
| Bilaç et al., 2021 | Turkey | Cross-sectional and case-control study | Androgenetic alopecia | Outpatients with androgenetic alopecia, n=68  healthy subjects,  n=30 | HADS-A | **HADS-D**  73.5% of outpatients with androgenetic alopecia had depressive symptoms | **7** |
| Guo et al., 2021 | China | Cross-sectional and observational study | Non-small cell lung cancer | Outpatients with non-small cell lung cancer, n=290 | SAS  HADS-A | **SDS, HADS-D**  47.6% of outpatients with non-small cell lung cancer had depressive symptoms (SDS score ≥ 50)  39.3% of outpatients with non-small cell lung cancer had depressive symptoms (HADS-D score ≥ 8) | **7** |
| Lin et al., 2021 | China | Cross-sectional and observational study | Atypical chest pain | Outpatients with atypical chest pain, n=122 | GAD-7  SAS | **PHQ-9, SDS**  16.4% of outpatients with atypical chest pain had moderate depressive symptoms (PHQ-9)  9.8% of outpatients with atypical chest pain had severe depressive symptoms (PHQ-9)  5.7% of outpatients with atypical chest pain had moderate depressive symptoms (SDS)  2.5% of outpatients with atypical chest pain had severe depressive symptoms (SDS) | **7** |
| Mishkin et al., 2021 | Kazakhstan | Cross-sectional and observational study | HIV | Outpatients  with HIV,  n=410 | GAD-7 | **PHQ-8**  12.7% of outpatients with HIV met PHQ-8 major depression criteria  8.5% of outpatients with HIV had anxiety (GAD 7 score >10) and major depression  19.3% of outpatients with HIV had anxiety (GAD 7 score >10) and major depression | **8** |
| Reference | **Country/City** | **Study Design** | **Diagnosis** | **Sample size** | **Instrument to assess anxiety** | **Psychopathological manifesttaions co-occurring with anxiety** | **JBI Checklist-total score** |
| Moayed et al., 2021 | Iran | Cross-sectional and observational study | COVID-19 | Inpatients  with COVID-19,  n=221 | DASS-21 | **DASS-21**  38.46% of inpatients with COVID-19 had severe depressive symptoms (DASS-21 score 11-13)  54.29% of inpatients with COVID-19 had very severe depressive symptoms (DASS-21 score >14) | **9** |
| Naser et al., 2021 | Jordan | Cross-sectional and observational study | Cancer | Inpatients/outpatients with cancer,  n=1,011 | HADS-A  GAD-7 | **HADS-D**  23.4% of inpatients/outpatients with cancer had depressive symptoms (HADS-D score > 8) | **8** |
| Park et al., 2021 | Korea | Cross-sectional and observational study | HIV | Outpatients  with HIV,  n=457 | STAI, Korean version | **BDI**  23.5% of HIV outpatients had depressive symptoms (BDI score ≥ 20) | **8** |
| Pinto et al., 2021 | India | Cross-sectional and observational study | Systemic lupus erythematosus | Outpatients with systemic lupus erythematosus,  n=140 | GAD-7 | **PHQ-9**  25% of outpatients with systemic lupus erythematosus had depressive symptoms (PHQ-9 score ≥ 10) | **7** |
| Pollo et al., 2021 | Brazil | Cross-sectional and observational study | Psoriasis | Outpatients  with psoriasis,  n=281 | HADS-A | **HADS-D**  19% of outpatients with psoriasis had depressive symptoms (HADS-D score > 8) | **8** |
| Reference | **Country/City** | **Study Design** | **Diagnosis** | **Sample size** | **Instrument to assess anxiety** | **Psychopathological manifesttaions co-occurring with anxiety** | **JBI Checklist-total score** |
| Sewtz et al., 2021 | Germany | Longitudinal and observational study | Cancer | Inpatients with  cancer undergoing  palliative care,  n=102 | HADS-A | **HADS-D**  56.7% of inpatients with cancer undergoing palliative care had depressive symptoms at the admission to the palliative care unit (HADS-D score ≥ 8)  73.4% of palliative care cancer inpatients had depressive symptoms at 2 weeks after discharge (HADS-D score ≥ 8) | **7** |
| Sharma et al., 2021 | Nepal | Cross-sectional and observational study | Type 2 diabetes | Outpatients with  type 2 diabetes,  n=296 | GAD-7 | **PHQ-9**  27.4% of outpatients with type 2 diabetes had mild depressive symptoms (PHQ-9 score 5-9);  19.6% of outpatients with type 2 diabetes had moderate depressive symptoms (PHQ-9 score 10-14);  2.4% of outpatients with type 2 diabetes had severe depressive symptoms (PHQ-9 score ≥20) | **9** |
| Takita et al., 2021 | Japan | Cross-sectional and observational study | Pulmonary hypertension | Outpatients with pulmonary hypertension,  n=74 | GAD-7 | **PHQ-9**  44.6% of outpatients with pulmonary hypertension had depressive symptoms (PHQ-9 score ≥ 5), among whom 17.6% had moderate to severe depressive symptoms (PHQ-9 score ≥ 10) | **7** |
| Vogt et al., 2021 | London | Cross-sectional and observational study | Chronic rhino sinusitis | Outpatients with chronic rhino sinusitis, n=95 | HADS-A | **HADS-D**  38.95% of outpatients with chronic rhino sinusitis had depressive symptoms | **8** |
| Reference | **Country/City** | **Study Design** | **Diagnosis** | **Sample size** | **Instrument to assess anxiety** | **Psychopathological manifesttaions co-occurring with anxiety** | **JBI Checklist-total score** |
| Yadav et al., 2021 | India | Cross-sectional and observational study | COVID-19 | Inpatients  with COVID-19,  n=100 | GAD-7 | **PHQ-9**  21% of inpatients with COVID-19 had mild depressive symptoms (PHQ-9 score 5-9);  4% of inpatients with COVID-19 had moderate depressive symptoms (PHQ-9 score 10-14);  2% of inpatients with COVID-19 had moderately severe symptoms (PHQ-9 score 15-19) | **7** |
| Zhang et al., 2021 | China | Cross-sectional and observational study | COVID-19 | Inpatients  with COVID-19, n=119 | M.I.N.I. | **M.I.N.I.**  5.9% of COVID-19 inpatients had a depressive disorder  9.24% of COVID-19 inpatients had post-traumatic stress syndrome or post-traumatic stress disorder | **8** |
| Zhong et al., 2021 | China | Cross-sectional and observational study | Epilepsy | Outpatients  with epilepsy,  n=320 | GAD-7 | **NDDI-E**  20.3% of outpatients with epilepsy had depressive symptoms | **7** |
| Zhou et al., 2021 | China | Cross-sectional and observational study | COVID-19 | Inpatients  with COVID-19,  n=65 | SAS | **SDS Chinese-version**  41.54% of inpatients with COVID-19 had depressive symptoms (SDS score > 72) | **8** |

**Note.** ADIS-IV: Anxiety Disorders Interview Schedule for DSM-IV; AIDS: Acquired Immune Deficiency Syndrome; ASI: Anxiety Sensitivity Index; BAI: Beck Anxiety Inventory; BDI: Beck Depression Inventory; BDI-II: Beck Depression Inventory-II; BDQ: Body Image Disturbance Questionnaire; CES-D: Center for Epidemiologic Studies Depression Scale; CIDI: Composite International Diagnostic Interview; CIDI-Auto: computerized version of the Composite International Diagnostic Interview; CMDI: Chicago Multiscale Depression Inventory; COVID-19: Coronavirus disease 2019; DASS: Depression Anxiety Stress Scales; DSM**:** Diagnostic and Statistical Manual of mental disorders; FSS: Fatigue Severity Scale; GAD-7: Generalized Anxiety Disorder Scale-7; GHQ: General Health Questionnaire; GDS-SF: Geriatric Depression Scale-Short Form; GHQ-28: General Health Questionnaire-28; GMHAT/PC: Global Mental Health Assessment Tool-Primary Care Version; HADS: Hospital Anxiety and Depression Scale; HADS-A: Hospital Anxiety and Depression Scale - Anxiety subscale; HADS-D: Hospital Anxiety and Depression Scale - Depression subscale; HAI-SF: Health Anxiety Inventory Short Form; HAM-A: Hamilton Anxiety Rating Scale; HAM-D: Hamilton Depression Rating Scale; HSCL-25: Hopkins Symptom Checklist-25; JBI: Joanna Briggs Institute; ICD: International Classification of Diseases; HIV: Human Immunodeficiency Virus; MQ: Maastricht Questionnaire; MAS: Manifest Anxiety Scale; MAX–PC: Memorial Anxiety Scale for Prostate Cancer; MILP: Monash Interview for Liaison Psychiatry; M.I.N.I.: Mini International Neuropsychiatric Interview; MOS-SS: Medical Outcomes Study Sleep Scale; MSE: clinical interview & Mental Status Examination; NDDI-E: Neurological Disorders Depression Inventory for Epilepsy; PHQ: Patient Health Questionnaire; PRIME-MD: Primary Care Evaluation of Mental Disorders; SAHD: Schedule of Attitudes towards Hastened Death; SAS: Zung Self-Rating Anxiety Scale; SCAN: Schedules for Clinical Assessment in Neuropsychiatry; SCL-90: Symptom Checklist-90; SCL-90-R: Symptom Checklist-90-Revised; SCID-UP-R: Structured Clinical Interview for DSM-III-R Upjohn version; SCID: Structured Clinical Interview; SDS: Zung Self-Rating Depression Scale; SQ: Kellner Symptom Questionnaire; STAI: State-Trait Anxiety Inventory; VAS: Visual Analogue Scale

**References**

Aass, N., Fosså, S. D., Dahl, A. A., and Aloe, T. J. (1997). Prevalence of anxiety and depression in cancer patients seen at the Norwegian Radium Hospital. Eur. J. Cancer. 33, 1597-1604. doi:10.1016/s0959-8049(97)00054-3

Abuelgasim, K. A., Ahmed, G. Y., Alqahtani, J. A., Alayed, A. M., Alaskar, A. S., and Malik, M. A. (2016). Depression and anxiety in patients with hematological malignancies, prevalence, and associated factors. Saudi Med. J. 37, 877-881. doi:10.15537/smj.2016.8.14597

Aggarwal, H. K., Jain, D., Dabas, G., and Yadav, R. K. (2017). Prevalence of depression, anxiety and insomnia in chronic kidney disease patients and their co-relation with the demographic variables. Prilozi, 38, 35-44. doi:10.1515/prilozi-2017-0020

Aggelopoulou, Z., Fotos, N. V., Chatziefstratiou, A. A., Giakoumidakis, K., Elefsiniotis, I., and Brokalaki, H. (2017). The level of anxiety, depression and quality of life among patients with heart failure in Greece. Appl. Nurs. Res. 34, 52-56. doi: 10.1016/j.apnr.2017.01.003

Al-Atram, A. A. (2018). Prevalence and patterns of psychiatric co-morbidity among adult medical inpatients: a cross-sectional study. Kuwait Med. J. 50, 410-416.

Al‐Rawashdeh, B. M., Saleh, M. Y. N., Mustafa, R. B., Alkhoujah, M. F., Elkhatib, A. H., Alsghaireen, H., et al. (2018). Prevalence of depression and anxiety among otolaryngology outpatients at Jordan University Hospital. Perspect. Psychiatr. Care. 55, 383-395. doi:10.1111/ppc.12320

Alacacioglu, A., Tarhan, O., Alacacioglu, I., Dirican, A., and Yilmaz, U. (2013). Depression and anxiety in cancer patients and their relatives. J. BUON. 18, 767-74.

AlBekairy, A., AbuRuz, S., Alsabani, B., Alshehri, A., Aldebasi, T., Alkatheri, A., et al. (2017). Exploring Factors Associated with Depression and Anxiety among Hospitalized Patients with Type 2 Diabetes Mellitus. Med. Princ. Pract. 26, 547-553. doi:10.1159/000484929

Allabadi, H., Alkaiyat, A., Alkhayyat, A., Hammoudi, A., Odeh, H., Shtayeh, J., et al. (2019). Depression and anxiety symptoms in cardiac patients: a cross-sectional hospital-based study in a Palestinian population. BMC Public. Health. 19, 232. doi:10.1186/s12889-019-6561-3

Alok, R., Das, S. K., Agarwal, G. G., Salwahan, L., and Srivastava, R. (2011). Relationship of severity of depression, anxiety and stress with severity of fibromyalgia.  Clin Exp Rheumatol, 29, S70-S72.

Alosaimi, F. D., Al-Sultan, O., Alghamdi, Q., Almohaimeed, I., and Alqannas, S. (2014). Association of help-seeking behavior with depression and anxiety disorders among gastroenterological patients in Saudi Arabia. Saudi J. Gastroenterol. 20, 233. doi:10.4103/1319-3767.136977

Alsaadi, T., El Hammasi, K., Shahrour, T. M., Shakra, M., Turkawi, L., Almaskari, B., et al. (2015). Prevalence of depression and anxiety among patients with epilepsy attending the epilepsy clinic at Sheikh Khalifa Medical City, UAE: A cross-sectional study. Epilepsy Behav. 52, 194-199. doi:10.1016/j.yebeh.2015.09.008

An, J., Zhou, H., Yang, T., Duan, C., Bao, C., Wan, C., et al. (2021). Relationship of psychological factors with daily activities and quality of life in patients with chronic obstructive pulmonary disease in a Chinese rural population. Ann. Palliat. Med. 10, 1675-1684. doi:10.21037/apm-20-1151

Anyfanti, P., Gavriilaki, E., Pyrpasopoulou, A., Triantafyllou, G., Triantafyllou, A., Chatzimichailidou, S., et al. (2016). Erratum to: Depression, anxiety, and quality of life in a large cohort of patients with rheumatic diseases: common, yet undertreated. Clin. Rheumatol. 35, 1411-1411. doi:10.1007/s10067-016-3252-7

Asadi-Pooya, A. A., and Sperling, M. R. (2011). Depression and anxiety in patients with epilepsy, with or without other chronic disorders. 
Iran. Red. Crescent Med. J. 13, 112-116.

Askari, F., Ghajarzadeh, M., Mohammadifar, M., Azimi, A., Sahraian, M. A., and Owji, M. (2014). Anxiety in patients with multiple sclerosis: association with disability, depression, disease type and sex. Acta Med. Iran. 52, 889-892.

Asghari, A., Mohammadi, F., Kamrava, S. K., Tavakoli, S., and Farhadi, M. (2012). Severity of depression and anxiety in obstructive sleep apnea syndrome. Eur. Arch. Oto-Rhino-Laryngol. 269, 2549-2553. doi:10.1007/s00405-012-1942-6

Axford, J., Butt, A., Heron, C., Hammond, J., Morgan, J., Alavi, A., et al. (2010). Prevalence of anxiety and depression in osteoarthritis: use of the Hospital Anxiety and Depression Scale as a screening tool. Clin. Rheumatol. 29, 1277-1283. doi:10.1007/s10067-010-1547-7

Azad, N., Gondal, M., and Abbas, N. (2008). Frequency of depression and anxiety in patients attending a rheumatology clinic. J. Coll. Physicians Surg. Pak. 18, 569-573.

Azizoddin, D. R., Zamora-Racaza, G., Ormseth, S. R., Sumner, L. A., Cost, C., Ayeroff, J. R., et al. (2017). Psychological Factors that Link Socioeconomic Status to Depression/Anxiety in Patients with Systemic Lupus Erythematosus. J. Clin. Psychol. Med. Settings. 24, 302-315. doi:10.1007/s10880-017-9505-z

Bachen, E. A., Chesney, M. A., and Criswell, L. A. (2009). Prevalence of mood and anxiety disorders in women with systemic lupus erythematosus. Arthritis. Rheum. 61, 822-829. doi:10.1002/art.24519

Bahar, R., Zhou, P., Liu, Y., Huang, Y., Phillips, A., Lee, T. K., et al. (2016). The prevalence of anxiety and depression in patients with or without hyperhidrosis (HH). J. Am. Acad. Dermatol. 75, 1126-1133. doi:10.1016/j.jaad.2016.07.001

Banerji, A., Davis, K. H., Brown, T. M., Hollis, K., Hunter, S. M., Long, J., et al. (2020). Patient-reported burden of hereditary angioedema: findings from a patient survey in the United States. Ann. Allergy Asthma Immunol. 124, 600-607. doi:10.1016/j.anai.2020.02.018

Baniasadi, N., Dehesh, M. M., Mohebbi, E., Hayatbakhsh Abbasi, M., and Oghabian, Z. (2017). Assessing the sleep quality and depression-anxiety-stress in irritable bowel syndrome patients. Arq. Gastroenterol. 54, 163-166. doi:10.1590/s0004-2803.201700000-08

Bankier, B., Januzzi, J. L., and Littman, A. B. (2004). The high prevalence of multiple psychiatric disorders in stable outpatients with coronary heart disease. Psychosom. Med. 66, 645-650. doi:10.1097/01.psy.0000138126.90551.62

Barlow, J. H., Cullen, L. A., and Rowe, I. F. (2002). Educational preferences, psychological well-being and self-efficacy among people with rheumatoid arthritis. Patient. Educ. Couns. 46, 11-19. doi:10.1016/s0738-3991(01)00146-x

Been, S. K., Schadé, A., Bassant, N., Kastelijns, M., Pogány, K., and Verbon, A. (2019). Anxiety, depression and treatment adherence among HIV-infected migrants. AIDS Care. 31, 979-987. doi:10.1080/09540121.2019.1601676

Beesley, V. L., Burge, M., Dumbrava, M., Callum, J., Neale, R. E., and Wyld, D. K. (2018). Perceptions of care and patient-reported outcomes in people living with neuroendocrine tumours. Support. Care Cancer, 26, 3153-3161. doi:10.1007/s00520-018-4166-5

Beiske, A. G., Svensson, E., Sandanger, I., Czujko, B., Pedersen, E. D., Aarseth, J. H., et al. (2008). Depression and anxiety amongst multiple sclerosis patients. Eur. J. Neurol. 15, 239-245. doi: 10.1111/j.1468-1331.2007.02041.x.

Beitman, B. D., Mukerji, V., Lamberti, J. W., Schmid, L., DeRosear, L., Kushner, M., et al. (1989). Panic disorder in patients with chest pain and angiographically normal coronary arteries. Am. J. Cardiol. 63, 1399-1403. doi:10.1016/0002-9149(89)91056-4

Bekir, M., Kocakaya, D., Balcan, B., Olgun Yildizeli, Ş., Eryüksel, E., and Ceyhan, B. (2020). Clinical impact of depression and anxiety in patients with non-cystic fibrosis bronchiectasis. Tuberk Toraks, 68, 103-111. doi:10.5578/tt.69348

Bektas, D. K., and Demir, S. (2016). Anxiety, Depression Levels and Quality of Life in Patients with Gastrointestinal Cancer in Turkey. Asian Pac. J. Cancer Prev. 17, 723-731. doi:10.7314/apjcp.2016.17.2.723

Bellin, M. H., Zabel, T. A., Dicianno, B. E., Levey, E., Garver, K., Linroth, R., et al. (2009). Correlates of Depressive and Anxiety Symptoms in Young Adults with Spina Bifida. J. Pediatr. Psychol. 35, 778-789. doi:10.1093/jpepsy/jsp094

Bertoli, E., and de Leeuw, R. (2016). Prevalence of Suicidal Ideation, Depression, and Anxiety in Chronic Temporomandibular Disorder Patients. J. Orofac. Pain. 296-301. doi:10.11607/ofph.1675

Beşirli, A., Alptekin, J. Ö., Kaymak, D., and Özer, Ö. A. (2019). The Relationship Between Anxiety, Depression, Suicidal Ideation and Quality of Life in Patients with Rheumatoid Arthritis. **Psychiatr. Q**. 91, 53-64. doi:10.1007/s11126-019-09680-x

Betancur, M. N., Lins, L., Oliveira, I. R. de, and Brites, C. (2017). Quality of life, anxiety and depression in patients with HIV/AIDS who present poor adherence to antiretroviral therapy: a cross-sectional study in Salvador, Brazil. Braz. J. Infect. Dis. 21, 507-514. doi:10.1016/j.bjid.2017.04.004

Beutel, M. E., Fischbeck, S., Binder, H., Blettner, M., Brähler, E., Emrich, K., et al. (2015). Depression, Anxiety and Quality of Life in Long-Term Survivors of Malignant Melanoma: A Register-Based Cohort Study. PLoS One. 10, e0116440. doi:10.1371/journal.pone.0116440

Bilaç, C., Öztürkcan, S., and Kutlubay, Z. (2021). Quality of life, anxiety and depression in patients with androgenetic alopecia. Hong Kong J. Dermatol. Venereol. 29, 13-22.

Bossola, M., Ciciarelli, C., Conte, G. L., Vulpio, C., Luciani, G., and Tazza, L. (2010). Correlates of symptoms of depression and anxiety in chronic hemodialysis patients. Gen. Hosp. Psychiatry. 32, 125-131. doi:10.1016/j.genhosppsych.2009.10.009

Bove, D. G., Lavesen, M., and Lindegaard, B. (2020). Characteristics and health related quality of life in a population with advanced chronic obstructive pulmonary disease, a cross-sectional study. BMC Palliat. Care. 19. doi:10.1186/s12904-020-00593-2

Bragança, G. M. G., Lima, S. O., Pinto Neto, A. F., Marques, L. M., Melo, E. V. de, and Reis, F. P. (2014). Evaluation of anxiety and depression prevalence in patients with primary severe hyperhidrosis. An Bras Dermatol. 89, 230-235. doi:10.1590/abd1806-4841.20142189

Brito, D. C. S. D., Machado, E. L., Reis, I. A., Carmo, L. P. D. F. D., and Cherchiglia, M. L. (2019). Depression and anxiety among patients undergoing dialysis and kidney transplantation: a cross-sectional study. Sao Paulo Med. J. 137, 137-147. doi:10.1590/1516-3180.2018.0272280119

Bucourt, E., Martaillé, V., Goupille, P., Joncker-Vannier, I., Huttenberger, B., Réveillère, C., et al. (2019). A Comparative Study of Fibromyalgia, Rheumatoid Arthritis, Spondyloarthritis, and Sjögren’s Syndrome; Impact of the Disease on Quality of Life, Psychological Adjustment, and Use of Coping Strategies. Pain Med. 22, 372-381. doi:10.1093/pm/pnz255

Bulat, V., Šitum, M., Lovrić, I., and Dediol, I. (2020). Study on the impact of psoriasis on quality of life: psychological, social and financial implications. Psychiatr. Danub. 32, 553-561.

Businco, L. D. R., Businco, A. D. R., Lauriello, M., and Tirelli, G. C. (2004). State and trait anxiety in patients affected by nasal polyposis before and after medical treatment. Acta Otorhinolaryngol. Ital. 24, 326-329.

Byrne, G., Rosenfeld, G., Leung, Y., Qian, H., Raudzus, J., Nunez, C., et al. (2017). Prevalence of anxiety and depression in patients with inflammatory bowel disease. Can. J. Gastroenterol. Hepatol. 2017, 1-6. doi:10.1155/2017/6496727

Calikoglu, E., Onder, M., Cosar, B., and Candansayar, S. (2001). Depression, anxiety levels and general psychological profile in Behçet’s disease. Dermatology. 203, 238-240. doi: 10.1159/000051756

Camara, A., Sow, M. S., Touré, A., Sako, F. B., Camara, I., Soumaoro, K., et al. (2020). Anxiety and depression among HIV patients of the infectious disease department of Conakry University Hospital in 2018. Epidemiol. Infect. 148. doi:10.1017/s095026881900222x

Cardoso, G., Graca, J., Klut, C., Trancas, B., and Papoila, A. (2015). Depression and anxiety symptoms following cancer diagnosis: a cross-sectional study. Psychol. Health Med. 21, 562-570. doi:10.1080/13548506.2015.1125006

Carvalho, I. G., Bertolli, E. D. S., Paiva, L., Rossi, L. A., Dantas, R. A. S., and Pompeo, D. A. (2016). Anxiety, depression, resilience and self-esteem in individuals with cardiovascular diseases. Rev. Lat. Am. Enfermagem. 24. doi:10.1590/1518-8345.1405.2836

Castro, M., Kraychete, D., Daltro, C., Lopes, J., Menezes, R., and Oliveira, I. (2009). Comorbid anxiety and depression disorders in patients with chronic pain. Arq. Neuropsiquiatr. 67, 982-985. doi:10.1590/s0004-282x2009000600004

Chan, H. N., Yap, H. L., Kanagasuntheram, N., and Chan, Y. H. (2009). Prevalence of depression and anxiety in hospitalized chronic obstructive pulmonary disease patients and their quality of life: a pilot study. Asia Pac. Psychiatry. 1, 130-137. doi:10.1111/j.1758-5872.2009.00040.x

Cheung, G., Patrick, C., Sullivan, G., Cooray, M., and Chang, C. L. (2012). Sensitivity and specificity of the Geriatric Anxiety Inventory and the Hospital Anxiety and Depression Scale in the detection of anxiety disorders in older people with chronic obstructive pulmonary disease. Int. Psychogeriatr. 24, 128-136. doi:10.1017/s1041610211001426

Chi, H., Jin, H., Wang, Z., Feng, T., Zeng, T., Shi, H., et al. (2020). Anxiety and depression in adult-onset Still’s disease patients and associations with health-related quality of life. Clin. Rheumatol. 39, 3723-3732. doi:10.1007/s10067-020-05094-4

Cleland, J. A., Lee, A. J., and Hall, S. (2007). Associations of depression and anxiety with gender, age, health-related quality of life and symptoms in primary care COPD patients. Fam. Pract. 24, 217-223. doi:10.1093/fampra/cmm009

Cordina, M., Fenech, A. G., Vassallo, J., and Cacciottolo, J. M. (2009). Anxiety and the management of asthma in an adult outpatient population. Ther. Adv. Respir. Dis. 3, 227-233. doi:10.1177/1753465809347038

Cordingley, L., Prajapati, R., Plant, D., Maskell, D., Morgan, C., Ali, F. R., et al. (2014). Impact of psychological factors on subjective disease activity assessments in patients with severe rheumatoid arthritis. Arthritis Care Res. 66, 861-868. doi:10.1002/acr.22249

Costa, E. C. V., Vale, S., Sobral, M., and Graça Pereira, M. (2015). Illness perceptions are the main predictors of depression and anxiety symptoms in patients with chronic pain. Psychol. Health Med. 21, 483-495. doi:10.1080/13548506.2015.1109673

Cui, S.-S., Du, J.-J., Fu, R., Lin, Y.-Q., Huang, P., He, Y.-C., et al. (2017). Prevalence and risk factors for depression and anxiety in Chinese patients with Parkinson disease. BMC Geriatr. 17. doi:10.1186/s12877-017-0666-2

Cui, Y., Xia, L., Zhao, Q., Chen, S., and Gu, Z. (2018). Anxiety and depression in primary Sjögren’s syndrome: a cross-sectional study. BMC Psychiatry. 18, 1-8. doi:10.1186/s12888-018-1715-x

Cukor, D., Coplan, J., Brown, C., Friedman, S., Newville, H., Safier, M., et al. (2008). Anxiety disorders in adults treated by hemodialysis: a single-center study. Am. J. Kidney Dis. 52, 128-136. doi:10.1053/j.ajkd.2008.02.300

Cumming, T. B., Blomstrand, C., Skoog, I., and Linden, T. (2016). The high prevalence of anxiety disorders after stroke. 
Am. J. Geriatr. Psychiatry. 24, 154-160. doi:10.1016/j.jagp.2015.06.003

Czyżewski, Ł., Frelik, P., Wyzgał, J., and Szarpak, Ł. (2018). Evaluation of Quality of Life and Severity of Depression, Anxiety, and Stress in Patients After Kidney Transplantation. Transplant. Proc. 50, 1733-1737. doi:10.1016/j.transproceed.2018.04.026

Damen, N. L. M., Pelle, A. J. M., Szabó, B. M., and Pedersen, S. S. (2012). Symptoms of anxiety and cardiac hospitalizations at 12 months in patients with heart failure. J. Gen. Intern. Med. 27, 345-350. doi:10.1007/s11606-011-1843-1

Daniëls, L. A., Oerlemans, S., Krol, A. D. G., Creutzberg, C. L., and van de Poll-Franse, L. V. (2014). Chronic fatigue in Hodgkin lymphoma survivors and associations with anxiety, depression and comorbidity. Br. J. Cancer. 110, 868-874. doi:10.1038/bjc.2013.779

Daştan, N. B., and Buzlu, S. (2011). Depression and anxiety levels in early stage Turkish breast cancer patients and related factors.
Asian Pac. J. Cancer Prev. 12, 137-141.

Degirmenci, T., Kalkan Oguzhanoglu, N., Sozeri Varma, G., Ozdel, O., and Fenkci, S. (2015). Psychological Symptoms in Obesity and Related Factors. Noro Psikiyatr. Ars. 52, 42-46. doi:10.5152/npa.2015.6904

De Kort, S., Kruimel, J. W., Sels, J. P., Arts, I. C. W., Schaper, N. C., and Masclee, A. A. M. (2012). Gastrointestinal symptoms in diabetes mellitus, and their relation to anxiety and depression. Diabetes Res. Clin. Pract. 96, 248-255. doi:10.1016/j.diabres.2012.01.021

Del Rosso, A., Mikhaylova, S., Baccini, M., Lupi, I., Matucci Cerinic, M., and Maddali Bongi, S. (2013). In systemic sclerosis, anxiety and depression assessed by hospital anxiety depression scale are independently associated with disability and psychological factors.  Biomed Res. Int. 2013. doi:10.1155/2013/507493

Delgado-Guay, M., Parsons, H. A., Li, Z., Palmer, J. L., and Bruera, E. (2008). Symptom distress in advanced cancer patients with anxiety and depression in the palliative care setting. Support. Care Cancer. 17, 573-579. doi:10.1007/s00520-008-0529-7

De Souza, E. A. P., and Salgado, P. C. B. (2006). A psychosocial view of anxiety and depression in epilepsy. Epilepsy Behav. 8, 232-238. doi:10.1016/j.yebeh.2005.10.011

Ding, T., Wang, X., Fu, A., Xu, L., and Lin, J. (2019). Anxiety and depression predict unfavorable survival in acute myeloid leukemia patients. Medicine. 98, e17314. doi:10.1097/md.0000000000017314

DiNicola, G., Julian, L., Gregorich, S. E., Blanc, P. D., and Katz, P. P. (2013). The role of social support in anxiety for persons with COPD. J. Psychosom. Res. 74, 110-115. doi:10.1016/j.jpsychores.2012.09.022

Dirik, G., and Karanci, A. N. (2010). Psychological distress in rheumatoid arthritis patients: an evaluation within the conservation of resources theory. **Psychol. Health.** 25, 617-632. doi:10.1080/08870440902721818

Dissanayaka, N. N., O'Sullivan, J. D., Pachana, N. A., Marsh, R., Silburn, P. A., White, E. X., et al. (2016). Disease-specific anxiety symptomatology in Parkinson's disease. Int. Psychogeriatr. 28, 1153-1163. doi:10.1017/s1041610215002410

Dissanayaka, N. N., Sellbach, A., Matheson, S., O'Sullivan, J. D., Silburn, P. A., Byrne, G. J., et al. (2010). Anxiety disorders in Parkinson's disease: prevalence and risk factors. Mov. Disord. 25, 838-845. doi:10.1002/mds.25937

Dogar, I. A., Khawaja, I. S., Azeem, M. W., Awan, H., Ayub, A., Iqbal, J., et al. (2008). Prevalence and risk factors for depression and anxiety in hospitalized cardiac patients in Pakistan. Psychiatry (Edgmont). 5, 38-41.

Dong, D., Lou, P., Wang, J., Zhang, P., Sun, J., Chang, G., et al. (2020). Interaction of sleep quality and anxiety on quality of life in individuals with type 2 diabetes mellitus. Health Qual. Life Outcomes. 18. doi:10.1186/s12955-020-01406-z

Doser, K., Andersen, E. W., Kenborg, L., Dalton, S. O., Jepsen, J. R. M., Krøyer, A., et al. (2020). Clinical characteristics and quality of life, depression, and anxiety in adults with neurofibromatosis type 1: A nationwide study. Am. J. Med. Genet. A. 182, 1704-1715. doi:10.1002/ajmg.a.61627

Du-Quiton, J., Wood, P. A., Burch, J. B., Grutsch, J. F., Gupta, D., Tyer, K., et al. (2010). Actigraphic assessment of daily sleep-activity pattern abnormalities reflects self-assessed depression and anxiety in outpatients with advanced non-small cell lung cancer. Psychooncology. 19, 180-189. doi:10.1002/pon.1539

El Filali, A., Bentata, Y., Ada, N., and Oneib, B. (2017). Depression and anxiety disorders in chronic hemodialysis patients and their quality of life: a cross-sectional study about 106 cases in the northeast of Morocco. Saudi J. Kidney Dis. Transpl. 28, 341-348. doi:10.4103/1319-2442.202785

El-Miedany, Y. M., and El Rasheed, A. H. (2002). Is anxiety a more common disorder than depression in rheumatoid arthritis? Joint Bone Spine. 69, 300-306. doi: 10.1016/s1297-319x(02)00368-8.

Engin, B., Uguz, F., Yilmaz, E., Özdemir, M., and Mevlitoglu, I. (2007). The levels of depression, anxiety and quality of life in patients with chronic idiopathic urticaria. J. Eur. Acad. Dermatol. Venereol. 22, 36-40. doi:10.1111/j.1468-3083.2007.02324.x

Esser, P., Mehnert‐Theuerkauf, A., Friedrich, M., Johansen, C., Brähler, E., Faller, H., et al. (2020). Risk and associated factors of depression and anxiety in men with prostate cancer: Results from a German multicenter study. Psychooncology. 29, 1604-1612. doi:10.1002/pon.5471

Faezi, S. T., Paragomi, P., Shahali, A., Akhlaghkhah, M., Akbarian, M., Akhlaghi, M., et al. (2017). Prevalence and severity of depression and anxiety in patients with systemic sclerosis: an epidemiologic survey and investigation of clinical correlates. J. Clin. Rheumatol. 23, 80-86. doi:10.1097/rhu.0000000000000428

Fattouh, N., Hallit, S., Salameh, P., Choueiry, G., Kazour, F., and Hallit, R. (2019). Prevalence and factors affecting the level of depression, anxiety, and stress in hospitalized patients with a chronic disease. Perspect. Psychiatr. Care. 55, 592-599. doi:10.1111/ppc.12369

Fava, G. A., Fava, M., Kellner, R., Serafini, E., and Mastrogiacomo, I. (1981). Depression, Hostility and Anxiety in Hyperprolactinemic Amenorrhea. Psychother Psychosom. 36, 122-128. doi:10.1159/000287535

Fava, G. A., Porcelli, P., Rafanelli, C., Mangelli, L., and Grandi, S. (2010). The Spectrum of Anxiety Disorders in the Medically Ill. J. Clin. Psychiatry. 71, 910-914. doi:10.4088/jcp.10m06000blu

Fava, G. A., Trombini, G., Grandi, S., Bernardi, M., Evangelisti, L. P., Santarsiero, G., et al. (1984). Depression and anxiety associated with secondary amenorrhea. Psychosomatics. 25, 905-908. doi:10.1016/s0033-3182(84)72922-7

Filippakis, A., Jara, J., Ventura, N., Scala, S., Scopa, C., Ruthazer, R., et al. (2018). A prospective study of benign fasciculation syndrome and anxiety. Muscle Nerve. 58, 852-854. doi:10.1002/mus.26193

Fortier, E., Alavi, M., Bruneau, J., Micallef, M., Perram, J., Sockalingam, S., et al. (2017). Depression, anxiety, and stress among people with chronic hepatitis C virus infection and a history of injecting drug use in New South Wales, Australia. J. Addict. Med. 11, 10-18. doi:10.1097/adm.0000000000000261

Fouche, A. S., Saunders, E. F. H., and Craig, T. (2014). Depression and anxiety in patients with hereditary angioedema.
Ann. Allergy Asthma Immunol. 112, 371-375. doi:10.1016/j.anai.2013.05.028

Fotos, N. V., Elefsiniotis, I., Patelarou, A., Giakoumidakis, K., Patelarou, E., Kouros, A., et al. (2018). Psychological disorders and quality of life among patients with chronic viral hepatitis: A single-center cross-sectional study with pair-matched healthy controls. Gastroenterol. Nurs. 41, 206-218. doi:10.1097/sga.0000000000000339

Freitas, T. H., Hyphantis, T. N., Andreoulakis, E., Quevedo, J., Miranda, H. L., Alves, G. S., et al. (2015). Religious coping and its influence on psychological distress, medication adherence, and quality of life in inflammatory bowel disease. Braz J Psychiatry, 37, 219-227. doi:10.1590/1516-4446-2014-1507

Fruewald, S., Loeffler-Stastka, H., Eher, R., Saletu, B., and Baumhacki, U. (2001). Depression and quality of life in multiple sclerosis. Acta Neurol. Scand. 104, 257-261. doi:10.1034/j.1600-0404.2001.00022.x

Galeazzi, G. M., Ferrari, S., Giaroli, G., Mackinnon, A., Merelli, E., Motti, L., et al. (2005). Psychiatric disorders and depression in multiple sclerosis outpatients: impact of disability and interferon beta therapy. Neurol. Sci. 26, 255-262. doi:10.1007/s10072-005-0468-8

Gallagher, R., and McKinley, S. (2009). Anxiety, depression and perceived control in patients having coronary artery bypass grafts. J. Adv. Nurs. 65, 2386-2396. doi:10.1111/j.1365-2648.2009.05101.x

Ganasegeran, K., Abdulrahman, S. A., Al-Dubai, S. A. R., Wan, T. S., Sangaran, S., and Perumal, M. (2019). A Cross-Sectional Study Exploring Perceived Depression, Anxiety and Stress among Chronic Pain Patients in a Malaysian General Hospital. Malaysian Journal of Psychiatry, 28, 48-62.

Ganasegeran, K., Renganathan, P., Manaf, R. A., and Al-Dubai, S. A. R. (2014). Factors associated with anxiety and depression among type 2 diabetes outpatients in Malaysia: a descriptive cross-sectional single-centre study. BMJ Open. 4, e004794. doi:10.1136/bmjopen-2014-004794

Gao, Y. H., Guan, W. J., Zhu, Y. N., Chen, R. C., and Zhang, G. J. (2018). Anxiety and depression in adult outpatients with bronchiectasis: Associations with disease severity and health‐related quality of life. Clin. Respir. J. 12, 1485-1494. doi:10.1111/crj.12695

Geue, K., Göbel, P., Leuteritz, K., Nowe, E., Sender, A., Stöbel‐Richter, Y., et al. (2019). Anxiety and depression in young adult German cancer patients: Time course and associated factors. Psychooncology. 28, 2083-2090. doi:10.1002/pon.5197

Giardino, N. D., Curtis, J. L., Andrei, A. C., Fan, V. S., Benditt, J. O., Lyubkin, M., et al. (2010). Anxiety is associated with diminished exercise performance and quality of life in severe emphysema: a cross-sectional study. Respir. Res. 11, 29. doi:10.1186/1465-9921-11-29

Golden, J., O'Dwyer, A. M., and Conroy, R. M. (2005). Depression and anxiety in patients with hepatitis C: prevalence, detection rates and risk factors. Gen. Hosp. Psychiatry. 27, 431-438. doi:10.1016/j.genhosppsych.2005.06.006

Gostoli, S., Bonomo, M., Roncuzzi, R., Biffi, M., Boriani, G., and Rafanelli, C. (2016). Psychological correlates, allostatic overload and clinical course in patients with implantable cardioverter defibrillator (ICD). Int. J. Cardiol. 220, 360-364. doi:10.1016/j.ijcard.2016.06.246

Grzesiak, M., Beszłej, J. A., Mulak, A., Szechiński, M., Szewczuk-Bogusławska, M., and Waszczuk, E. (2014). The lifetime prevalence of anxiety disorders among patients with irritable bowel syndrome. Adv. Clin. Exp. Med. 23, 987-992. doi:10.17219/acem/37356

Gu, G., Zhou, Y., Zhang, Y., and Cui, W. (2016). Increased prevalence of anxiety and depression symptoms in patients with coronary artery disease before and after percutaneous coronary intervention treatment. BMC Psychiatry. 16, 1-9. doi:10.1186/s12888-016-0972-9

Guidi, J., Fava, G. A., Picardi, A., Porcelli, P., Bellomo, A., Grandi, S., et al. (2011). Subtyping depression in the medically ill by cluster analysis. J. Affect. Disord. 132, 383-388. doi:10.1016/j.jad.2011.03.004

Gullich, I., Ramos, A. B., Zan, T. R. A., Scherer, C., and Mendoza-Sassi, R. A. (2013). Prevalence of anxiety in patients admitted to a university hospital in southern Brazil and associated factors. Rev. Bras. Epidemiol. 16, 644-657. doi:10.1590/s1415-790x2013000300009

Guo, C., and Huang, X. (2021). Hospital anxiety and depression scale exhibits good consistency but shorter assessment time than Zung self-rating anxiety/depression scale for evaluating anxiety/depression in non-small cell lung cancer. Medicine. 100, e24428. doi:10.1097/md.0000000000024428

Guzelhan, Y., Conkbayir, C., Ugurlucan, M., Yildiz, C. E., Alpagut, U., and Bozbuga, N. (2018). Gender Differences in Patients with Anxiety after Coronary Artery Bypass Surgery. Heart Surg. Forum. 21, E165-E169. doi:10.1532/hsf.1451

Hamidizadeh, N., Ranjbar, S., Ghanizadeh, A., Parvizi, M. M., Jafari, P., and Handjani, F. (2020). Evaluating prevalence of depression, anxiety and hopelessness in patients with Vitiligo on an Iranian population. Health Qual. Life Outcomes. 18. doi:10.1186/s12955-020-1278-7

Hampton, S. N., Nakonezny, P. A., Richard, H. M., and Wells, J. E. (2019). Pain catastrophizing, anxiety, and depression in hip pathology. Bone Joint J. 101-B, 800-807. doi:10.1302/0301-620x.101b7.bjj-2018-1309.r1

Hanna, M., and Strober, L. B. (2020). Anxiety and depression in Multiple Sclerosis (MS): Antecedents, consequences, and differential impact on well-being and quality of life. Mult. Scler. Relat. Disord. 44, 102261. doi:10.1016/j.msard.2020.102261

Hao, F., Tam, W., Hu, X., Tan, W., Jiang, L., Jiang, X., et al. (2020). A quantitative and qualitative study on the neuropsychiatric sequelae of acutely ill COVID-19 inpatients in isolation facilities. Transl. Psychiatry. 10, 1-14. doi:10.1038/s41398-020-01039-2

Hashiro, M., and Okumura, M. (1994). Anxiety, depression, psychosomatic symptoms and autonomic nervous function in patients with chronic urticaria. J. Dermatol. Sci. 6, 95. doi:10.1016/0923-1811(93)91267-x

Häuser, W., Janke, K. H., Klump, B., and Hinz, A. (2011). Anxiety and depression in patients with inflammatory bowel disease: comparisons with chronic liver disease patients and the general population. Inflamm. Bowel Dis. 17, 621-632. doi:10.1002/ibd.21346

Havermans, T., Colpaert, K., and Dupont, L. J. (2008). Quality of life in patients with Cystic Fibrosis: Association with anxiety and depression. J. Cyst. Fibros. 7, 581-584. doi:10.1016/j.jcf.2008.05.010

Hawamdeh, Z. (2008). Assessment of anxiety and depression after lower limb amputation in Jordanian patients. Neuropsychiatr. Dis. Treat. 627. doi:10.2147/ndt.s2541

Haworth, J. E., Moniz‐Cook, E., Clark, A. L., Wang, M., Waddington, R., and Cleland, J. G. F. (2005). Prevalence and predictors of anxiety and depression in a sample of chronic heart failure patients with left ventricular systolic dysfunction. Eur. J. Heart Fail. 7, 803-808. doi:10.1016/j.ejheart.2005.03.001

Henry, A., Tourbah, A., Camus, G., Deschamps, R., Mailhan, L., Castex, C., et al. (2019). Anxiety and depression in patients with multiple sclerosis: The mediating effects of perceived social support. Mult. Scler. Relat. Disord. 27, 46-51. doi:10.1016/j.msard.2018.09.039

Ho, R. C., Fu, E. H., Chua, A. N., Cheak, A. A., and Mak, A. (2011). Clinical and psychosocial factors associated with depression and anxiety in Singaporean patients with rheumatoid arthritis. Int. J. Rheum. Dis. 14, 37-47. doi:10.1111/j.1756-185x.2010.01591.x

Ho, C. S. H., Lu, Y., Ndukwe, N., Chew, M. W. P., Shabbir, A., and Ho, R. C. M. (2018). Symptoms of Anxiety and Depression in Obese Singaporeans: a Preliminary Study. (2018). East Asian Arch. Psychiatry. 28. doi:10.12809/eaap181723

Holland, A. E., Fiore Jr, J. F., Bell, E. C., Goh, N., Westall, G., Symons, K., et al. (2014). Dyspnea and comorbidity contribute to anxiety and depression in interstitial lung disease. Respirology. 19, 1215-1221. doi:10.1111/resp.12360

Honarmand, K., and Feinstein, A. (2009). Validation of the Hospital Anxiety and Depression Scale for use with multiple sclerosis patients. Mult. Scler. J. 15, 1518-1524. doi:10.1177/1352458509347150

Hong, J. S., and Tian, J. (2014). Prevalence of anxiety and depression and their risk factors in Chinese cancer patients. Support. Care Cancer. 22, 453-459. doi:10.1007/s00520-013-1997-y

Huffman, J. C., Smith, F. A., Blais, M. A., Beiser, M. E., Januzzi, J. L., and Fricchione, G. L. (2006). Recognition and treatment of depression and anxiety in patients with acute myocardial infarction. Am. J. Cardiol. 98, 319-324. doi:10.1016/j.amjcard.2006.02.033

Jamshidi, A. R., Banihashemi, A. T., Paragomi, P., Hasanzadeh, M., Barghamdi, M., and Ghoroghi, S. (2016). Anxiety and depression in rheumatoid arthritis: an epidemiologic survey and investigation of clinical correlates in Iranian population. Rheumatol. Int. 36, 1119-1125. doi:10.1007/s00296-016-3493-4

Jia, S., Shi, X., Liu, G., Wang, L., Zhang, X., Ma, X., et al. (2020). Determinants of anxiety and depression in patients with cubital tunnel syndrome. BMC Psychiatry. 20. doi:10.1186/s12888-020-02934-0

Jones, K. H., Ford, D. V., Jones, P. A., John, A., Middleton, R. M., Lockhart-Jones, H., et al. (2012). A Large-Scale Study of Anxiety and Depression in People with Multiple Sclerosis: A Survey via the Web Portal of the UK MS Register. PLoS One. 7, e41910. doi:10.1371/journal.pone.0041910

Kala, P., Hudakova, N., Jurajda, M., Kasparek, T., Ustohal, L., Parenica, J., et al. (2016). Depression and anxiety after acute myocardial infarction treated by primary PCI. PLoS One. 11, e0152367. doi:10.1371/journal.pone.0152367

Kapısız, Ö., and Eker, F. (2018). Evaluation of the relationshipbetween the levels and perceptions of dyspnea and the levels of anxiety and depression in chronic obstructive pulmonary disease (COPD) patients. J. Psychiatric Nurs. 9, 88-95. doi:10.14744/phd.2018.53244

Karahan, A. Y., Kucuk, A. D. E. M., Balkarli, A. Y. Ş. E., Kayhan, F., Ozhan, N., Nas, O., et al. (2016). Alexithymia, depression, anxiety levels and quality of life in patients with rheumatoid arthritis. Acta Medica Mediterr. 32, 1675-1682. doi: 10.19193/0393-6384_2016_5_149

Karajgi, B., Rifkin, A., Doddi, S., and Kolli, R. (1990). The prevalence of anxiety disorders in patients with chronic obstructive pulmonary disease. Am. J. Psychiatry. 147, 200-201. doi:10.1176/ajp.147.2.200

Karakoyun-Celik, O., Gorken, I., Sahin, S., Orcin, E., Alanyali, H., and Kinay, M. (2009). Depression and anxiety levels in woman under follow-up for breast cancer: relationship to coping with cancer and quality of life. Med. Oncol. 27, 108-113. doi:10.1007/s12032-009-9181-4

Karimi, S., Andayeshgar, B., and Khatony, A. (2020). Prevalence of anxiety, depression, and stress in patients with multiple sclerosis in Kermanshah-Iran: a cross-sectional study. BMC Psychiatry. 20. doi:10.1186/s12888-020-02579-z

Karlıdağ, G., and Atmaca, M. (2019). The relationship between duration, stage and, treatment of illness and depression or anxiety in patients with chronic hepatitis B.  Anadolu Psikiyatri Derg. 20, 515-521. doi:10.5455/apd.27821

Katotomichelakis, M., Simopoulos, E., Zhang, N., Tripsianis, G., Danielides, G., Livaditis, M., et al. (2013). Olfactory dysfunction and asthma as risk factors for poor quality of life in upper airway diseases. Am. J. Rhinol. Allergy. 27, 293-298. doi:10.2500/ajra.2013.27.3903

Kayhan, F., Albayrak Gezer, İ., Kayhan, A., Kitiş, S., and Gölen, M. (2015). Mood and anxiety disorders in patients with chronic low back and neck pain caused by disc herniation. Int. J. Psychiatry Clin. 20, 19-23. doi:10.3109/13651501.2015.1100314

Kayhan, F., Cıcek, E., Uguz, F., Karababa, İ. F., and Kucur, R. (2013). Mood and anxiety disorders among inpatients of a university hospital in Turkey. Gen. Hosp. Psychiatry. 35, 417-422. doi:10.1016/j.genhosppsych.2013.03.004

Kayhan, F., Küçük, A., Satan, Y., İlgün, E., Arslan, Ş., and İlik, F. (2016). Sexual dysfunction, mood, anxiety, and personality disorders in female patients with fibromyalgia. Neuropsychiatr. Dis. Treat. 12, 349-355. doi:10.2147/ndt.s99160

Kellner, R., Buckman, M. T., Fava, G. A., and Pathak, D. (1984). Hyperprolactinemia, distress, and hostility. Am. J. Psychiatry. 141, 759-763. doi:10.1176/ajp.141.6.759

Khan, P., Qayyum, N., Malik, F., Khan, T., Khan, M., and Tahir, A. (2019). Incidence of Anxiety and Depression Among Patients with Type 2 Diabetes and the Predicting Factors. Cureus. 11. doi:10.7759/cureus.4254

Kheirandish, M., Faezi, S. T., Paragomi, P., Akhlaghi, M., Gharibdoost, F., Shahali, A., et al. (2014). Prevalence and severity of depression and anxiety in patients with systemic lupus erythematosus: An epidemiologic study in Iranian patients. Mod. Rheumatol. 25, 405-409. doi:10.3109/14397595.2014.962241

Khoury, B., Musallam, K. M., Abi-Habib, R., Bazzi, L., Al Ward, Z., Succar, J., et al. (2012). Prevalence of Depression and Anxiety in Adult Patients with β-Thalassemia Major and Intermedia. Int. J. Psychiatry Med. 44, 291-303. doi:10.2190/pm.44.4.a

Kissane, D. W., Grabsch, B., Love, A., Clarke, D. M., Bloch, S., and Smith, G. C. (2004). Psychiatric Disorder in Women with Early Stage and Advanced Breast Cancer: a Comparative Analysis. Aust. N. Z. J. Psychiatry. 38, 320-326. doi:10.1080/j.1440-1614.2004.01358.x

Klarić, M., Letica, I., Petrov, B., Tomić, M., Klarić, B., Letica, L., et al. (2009). Depression and anxiety in patients on chronic hemodialysis in University Clinical Hospital Mostar. Coll. Antropol. 33, 153-158.

Kohen, D., Burgess, A. P., Catalan, J., and Lant, A. (1998). The role of anxiety and depression in quality of life and symptom reporting in people with diabetes mellitus. Qual. Life Res. 7, 197204. doi:10.1023/a:1008817812520

Kokoszka, A., Leszczyńska, K., Radzio, R., Daniewska, D., Łukasiewicz, A., Orzechowski, W. M., et al. (2016). Prevalence of depressive and anxiety disorders in dialysis patients with chronic kidney disease. Arch. Psychiatry Psychother. 1, 8-13. doi:10.12740/app/61977

Korostil, M., and Feinstein, A. (2007). Anxiety disorders and their clinical correlates in multiple sclerosis patients. Mult. Scler. J. 13, 67-72. doi:10.1177/1352458506071161

Kouris, A., Armyra, K., Christodoulou, C., Katoulis, A., Potouridou, I., Tsatovidou, R., et al. (2015). Quality of life, anxiety, depression and obsessive-compulsive tendencies in patients with chronic hand eczema. Contact Dermatitis. 72, 367-370. doi:10.1111/cod.12366

Kovacs, A. H., Saidi, A. S., Kuhl, E. A., Sears, S. F., Silversides, C., Harrison, J. L., et al. (2009). Depression and anxiety in adult congenital heart disease: predictors and prevalence. Int. J. Cardiol. 137, 158-164. doi:10.1016/j.ijcard.2008.06.042

Kugler, C., Bara, C., von Waldthausen, T., Einhorn, I., Haastert, B., Fegbeutel, C., et al. (2014). Association of depression symptoms with quality of life and chronic artery vasculopathy: a cross-sectional study in heart transplant patients. J. Psychosom. Res. 77, 128-134. doi:10.1016/j.jpsychores.2014.06.007

Kwan, Z., Bong, Y. B., Tan, L. L., Lim, S. X., Yong, A. S. W., Ch’ng, C. C., et al. (2018). Determinants of quality of life and psychological status in adults with psoriasis. Arch. Dermatol. Res. 310, 443-451. doi:10.1007/s00403-018-1832-x

Łabuz-Roszak, B., Niewiadomska, E., Kubicka-Bączyk, K., Skrzypek, M., Dobrakowski, P., Tyrpień-Golder, K., et al. (2019). Prevalence of pain in patients with multiple sclerosis and its association with anxiety, depressive symptoms and quality of life. Psychiatr. Pol. 53, 475-486. doi:10.12740/pp/94469

Lantéri-Minet, M., Radat, F., Chautard, M. H., and Lucas, C. (2005). Anxiety and depression associated with migraine: influence on migraine subjects' disability and quality of life, and acute migraine management. Pain, 118, 319-326. doi:10.1016/j.pain.2005.09.010

Larice, S., Ghiggia, A., Di Tella, M., Romeo, A., Gasparetto, E., Fusaro, E., et al. (2019). Pain appraisal and quality of life in 108 outpatients with rheumatoid arthritis. Scand. J. Psychol. 61, 271-280. doi:10.1111/sjop.12592

Lavoie, K. L., Bacon, S. L., Barone, S., Cartier, A., Ditto, B., and Labrecque, M. (2006). What is worse for asthma control and quality of life: depressive disorders, anxiety disorders, or both?. Chest. 130, 1039-1047. doi:10.1378/chest.130.4.1039

Lee, Y. J., Kim, M. S., Cho, S., and Kim, S. R. (2013). Association of depression and anxiety with reduced quality of life in patients with predialysis chronic kidney disease. Int. J. Clin. Pract. Suppl. 67, 363-368. doi:10.1111/ijcp.12020

Leentjens, A. F., Dujardin, K., Marsh, L., Martinez‐Martin, P., Richard, I. H., and Starkstein, S. E. (2011). Symptomatology and markers of anxiety disorders in Parkinson's disease: a cross‐sectional study. Mov. Disord. 26, 484-492. doi:10.1002/mds.23528

Leite, C. C., and Maia, Â. C. (2013). Symptoms of disease and psychological adaptation in Brazilian scleroderma patients. Rev. Bras. Reumatol. Engl. Ed. 53, 405-411. doi:10.1016/s2255-5021(13)70111-1

Lewis, A. R., Wang, X., Magdalani, L., D’Arienzo, P., Bashir, C., Mansoor, W., et al. (2018). Health-related quality of life, anxiety, depression and impulsivity in patients with advanced gastroenteropancreatic neuroendocrine tumours. World J. Gastroenterol. 24, 671-679. doi:10.3748/wjg.v24.i6.671

Lewko, J., Zarzycki, W., and Krajewska-Kulak, E. (2012). Relationship between the occurrence of symptoms of anxiety and depression, quality of life, and level of acceptance of illness in patients with type 2 diabetes. Saudi Med. J. 33, 887-94.

Li, G., Wang, G., Hsu, F.-C., Xu, J., Pei, X., Zhao, B., et al. (2020a). Effects of Depression, Anxiety, Stigma, and Disclosure on Health-Related Quality of Life among Chronic Hepatitis B Patients in Dalian, China. Am. J. Trop. Med. Hyg. 102, 988-994. doi:10.4269/ajtmh.19-0007

Li, L., Zhao, Y., and Li, H. (2020b). Assessment of anxiety and depression in patients with incidental pulmonary nodules and analysis of its related impact factors. Thorac. Cancer. 11, 1433-1442. doi:10.1111/1759-7714.13406

Lima, C. de A. de, Oliveira, R. C. de, Oliveira, S. A. G. de, Silva, M. A. S. da, Lima, A. de A., Andrade, M. S., et al. (2020). Quality of life, anxiety and depression in patients with chronic obstructive pulmonary disease. Rev. Bras. Enferm. 73(suppl 1). doi:10.1590/0034-7167-2019-0423

Lin, Q., Bonkano, O., Wu, K., Liu, Q., Ali Ibrahim, T., and Liu, L. (2021). The Value of Chinese Version GAD-7 and PHQ-9 to Screen Anxiety and Depression in Chinese Outpatients with Atypical Chest Pain. Ther. Clin. Risk Manag. 17, 423-431. doi:10.2147/tcrm.s305623

Lin, C.-Y., and Pakpour, A. H. (2017). Using Hospital Anxiety and Depression Scale (HADS) on patients with epilepsy: Confirmatory factor analysis and Rasch models. Seizure, 45, 42-46. doi:10.1016/j.seizure.2016.11.019

Lincoln, N. B., Brinkmann, N., Cunningham, S., Dejaeger, E., De Weerdt, W., Jenni, W., et al. (2013). Anxiety and depression after stroke: a 5 year follow-up. 
Disabil. Rehabil. 35, 140-145. doi:10.3109/09638288.2012.691939

Liu, S., Wu, R., Li, L., Liu, L., Li, G., Zhang, X., et al. (2014). The prevalence of anxiety and depression in Chinese asthma patients. PLoS One. 9, e103014. doi:10.1371/journal.pone.0103014

Liu, D., Baumeister, R. F., Veilleux, J. C., Chen, C., Liu, W., Yue, Y., et al. (2020). Risk factors associated with mental illness in hospital discharged patients infected with COVID-19 in Wuhan, China. Psychiatry Res. 292, 113297. doi:10.1016/j.psychres.2020.113297

Lomper, K., Chudiak, A., Uchmanowicz, I., Rosińczuk, J., and Jankowska-Polanska, B. (2016). Effects of depression and anxiety on asthma-related quality of life. Adv. Respir. Med. 84, 212-221. doi:10.5603/piap.2016.0026

Lou, Z., Li, Y., Yang, Y., Wang, L., and Yang, J. (2015). Affects of Anxiety and Depression on Health-Related Quality of Life among Patients with Benign Breast Lumps Diagnosed via Ultrasonography in China. Int. J. Environ. Res. Public Health. 12, 10587-10601. doi:10.3390/ijerph120910587

Löwe, B., Gräfe, K., Ufer, C., Kroenke, K., Grünig, E., Herzog, W., et al. (2004). Anxiety and depression in patients with pulmonary hypertension. Psychosom. Med. 66, 831-836. doi:10.1097/01.psy.0000145593.37594.39

Lukaviciute, L., Ganceviciene, R., Navickas, P., Navickas, A., Grigaitiene, J., and Zouboulis, C. C. (2020). Anxiety, Depression, and Suicidal Ideation amongst Patients with Facial Dermatoses (Acne, Rosacea, Perioral Dermatitis, and Folliculitis) in Lithuania. Dermatology, 236, 314-322. doi:10.1159/000506627

Ma, Q., Su, K., Fu, Z., Wang, P., and Shi, H. (2020). Anxiety and depression in patients with nasal septal deviation. Am. J. Otolaryngol. 41, 102450. doi:10.1016/j.amjoto.2020.102450

Macaron, G., Fahed, M., Matar, D., Bou-Khalil, R., Kazour, F., Nehme-Chlela, D., et al. (2014). Anxiety, depression and suicidal ideation in Lebanese patients undergoing hemodialysis. Community Ment. Health J. 50, 235-238. doi:10.1007/s10597-013-9669-4

Magyari, F., Kósa, K., Berecz, R., Illés, A., Miltényi, Z., Simon, Z., et al. (2017). Employment status and health related quality of life among Hodgkin-lymphoma survivors’– results based on data from a major treatment center in Hungary. Health Qual. Life Outcomes. 15. doi:10.1186/s12955-017-0758-x

Malakouti, S. K., Mahmoudian, M. N. S., Alifattahi, N., and Salehi, M. (2011). Comorbidity of chronic tinnitus and mental disorders. Int. Tinnitus J. 16, 118-122.

Mani, J., Neuberth, M.-T., Fettel, J., Senf, B., Khoder, W., and Vallo, S. (2020). Quality of Patient Information by Urologists Is Associated with Mental Distress in Bladder Cancer Patients. Oncol. Res. and Treat. 43, 228-236. doi:10.1159/000507094

Marinus, J., Leentjens, A. F., Visser, M., Stiggelbout, A. M., and van Hilten, J. J. (2002). Evaluation of the hospital anxiety and depression scale in patients with Parkinson's disease. Clin. Neuropharmacol. 25, 318-324. doi:10.1097/00002826-200211000-00008

Matsushita, T., Matsushima, E., and Maruyama, M. (2005). Anxiety and depression of patients with digestive cancer. Psychiatry Clin. Neurosci. 59, 576-583. doi:10.1111/j.1440-1819.2005.01417.x

Means-Christensen, A. J., Roy-Byrne, P. P., Sherbourne, C. D., Craske, M. G., and Stein, M. B. (2008). Relationships among pain, anxiety, and depression in primary care. Depress Anxiety. 25, 593-600. doi:10.1002/da.20342

Menza, M. A., Robertson-Hoffman, D. E., and Bonapace, A. S. (1993). Parkinson's disease and anxiety: comorbidity with depression. Biol. Psychiatry. 34, 465-470. doi:10.1016/0006-3223(93)90237-8

Mielcarek, P., Nowicka-Sauer, K., and Kozaka, J. (2016). Anxiety and depression in patients with advanced ovarian cancer: a prospective study. J. Psychosom. Obstet. Gynecol. 37, 57-67. doi:10.3109/0167482x.2016.1141891

Milligan, F., Martinez, F., Aal, S. H. M. A., Ahmed, S. A., Joby, B., Matalam, J. S., et al. (2018). Assessing anxiety and depression in cancer patients. Br. J. Community Nurs. 27, S18-S23. doi:10.12968/bjon.2018.27.10.s18

Mishkin, K., Nugmanova, Z., Urbaeva, J., Nugumanova, G., Abdumananova, M., Kim, E., et al. (2021). Anxiety and depression among women living with HIV in Kazakhstan. AIDS Care. 33, 172-179. doi: 10.1080/09540121.2020.1719277

Moayed, M. S., Vahedian-Azimi, A., Mirmomeni, G., Rahimi-Bashar, F., Goharimoghadam, K., Pourhoseingholi, M. A., et al. (2021). Depression, Anxiety, and Stress Among Patients with COVID-19: A Cross-Sectional Study. Adv. Exp. Med. Biol. 131, 229-236. doi:10.1007/978-3-030-59261-5_19

Mondolo, F., Jahanshahi, M., Granà, A., Biasutti, E., Cacciatori, E., and Di Benedetto, P. (2007). Evaluation of anxiety in Parkinson’s disease with some commonly used rating scales. Neurol. Sci. 28, 270-275. doi:10.1007/s10072-007-0834-9

Montin, L., Leino-Kilpi, H., Katajisto, J., Lepistö, J., Kettunen, J., and Suominen, T. (2007). Anxiety and health-related quality of life of patients undergoing total hip arthroplasty for osteoarthritis. Chronic Illn. 3, 219-227. doi:10.1177/1742395307084405

Montserrat-Capdevila, J., Godoy, P., Marsal, J. R., Barbé, F., Pifarré, J., Alsedà, M., et al. (2017). Overview of the impact of depression and anxiety in chronic obstructive pulmonary disease. Lung, 195, 77-85. doi:10.1007/s00408-016-9966-0

Moryś, J. M., Bellwon, J., Adamczyk, K., and Gruchała, M. (2016). Depression and anxiety in patients with coronary artery disease, measured by means of self-report measures and clinician-rated instrument. Kardiol. Pol. 74, 53-60. doi:10.5603/kp.a2015.0116

Mosleh, H., and Alenezi, M. (2020). Prevalence and factors of anxiety and depression in chronic kidney disease patients undergoing hemodialysis: a cross-sectional single-center study in Saudi Arabia. Cureus. 12. doi:10.7759/cureus.6668

Müller-Tasch, T., Löwe, B., Lossnitzer, N., Frankenstein, L., Täger, T., Haass, M., et al. (2017). Anxiety and self-care behaviour in patients with chronic systolic heart failure: A multivariate model. Eur. J. Cardiovasc. Nurs. 17, 170-177. doi:10.1177/1474515117722255

Nahon, S., Lahmek, P., Saas, C., Durance, C., Olympie, A., Lesgourgues, B., et al. (2011). Socioeconomic and psychological factors associated with nonadherence to treatment in inflammatory bowel disease patients: results of the ISSEO survey. Inflamm. Bowel Dis. 17, 1270-1276. doi:10.1002/ibd.21482

Nakagawa, R., Yamaguchi, S., Kimura, S., Sadamasu, A., Yamamoto, Y., Sato, Y., et al. (2017). Association of Anxiety and Depression With Pain and Quality of Life in Patients With Chronic Foot and Ankle Diseases. Foot Ankle Int. 38, 1192-1198. doi:10.1177/1071100717723133

Nascimento, I., Nardi, A. E., Valença, A. M., Lopes, F. L., Mezzasalma, M. A., Nascentes, R., et al. (2002). Psychiatric disorders in asthmatic outpatients. Psychiatry Res. 110, 73-80. doi:10.1016/s0165-1781(02)00029-x

Naser, A. Y., Hameed, A. N., Mustafa, N., Alwafi, H., Dahmash, E. Z., Alyami, H. S., et al. (2021). Depression and Anxiety in Patients With Cancer: A Cross-Sectional Study. Front. Psychol. 12. doi:10.3389/fpsyg.2021.585534

Navabi, S., Gorrepati, V. S., Yadav, S., Chintanaboina, J., Maher, S., Demuth, P., et al. (2018). Influences and impact of anxiety and depression in the setting of inflammatory bowel disease. Inflamm. Bowel Dis. 24, 2303-2308. doi:10.1093/ibd/izy143

Nenadović, M., Jašović-Gašić, M., Vićentić, S., Nenadović, N., and Simonović, P. (2011). Anxiety in epileptic patients. Psychiatr. Danub. 23, 264-269.

Nie, X.-D., Wang, Q., Wang, M.-N., Zhao, S., Liu, L., Zhu, Y.-L., et al. (2020). Anxiety and depression and its correlates in patients with coronavirus disease 2019 in Wuhan. Int. J. Psychiatry Clin. 25, 109-114. doi:10.1080/13651501.2020.1791345

Noy, S., Achiron, A., Gabbay, U., Barak, Y., Rotstein, Z., Laor, N., et al. (1995). A new approach to affective symptoms in relapsing-remitting multiple sclerosis. Compr. Psychiatry. 36, 390-395. doi: 10.1016/s0010-440x(95)90121-3.

Nowicka-Sauer, K., Hajduk, A., Kujawska-Danecka, H., Banaszkiewicz, D., Smoleńska, Ż., Czuszyńska, Z., et al. (2018). Illness perception is significantly determined by depression and anxiety in systemic lupus erythematosus. Lupus, 27, 454-460. doi:10.1177/0961203317751858

Okamoto, Y., Motomura, N., Murashima, S., and Takamoto, S. (2013). Anxiety and depression after thoracic aortic surgery or coronary artery bypass.
Asian Cardiovasc. Thorac. Ann. 21, 22-30. doi:10.1177/0218492312444283

Olsen, S. J., Schirmer, H., Wilsgaard, T., Bønaa, K. H., and Hanssen, T. A. (2018). Cardiac rehabilitation and symptoms of anxiety and depression after percutaneous coronary intervention. Eur. J. Prev. Cardiol. 25, 1017-1025. doi:10.1177/2047487318778088

Olveira, C., Sole, A., Girón, R. M., Quintana-Gallego, E., Mondejar, P., Baranda, F., et al. (2016). Depression and anxiety symptoms in Spanish adult patients with cystic fibrosis: associations with health-related quality of life. Gen. Hosp. Psychiatry. 40, 39-46. doi:10.1016/j.genhosppsych.2016.02.002

On, Z. X., Grant, J., Shi, Z., Taylor, A. W., Wittert, G. A., Tully, P. J., et al. (2017). The association between gastroesophageal reflux disease with sleep quality, depression, and anxiety in a cohort study of Australian men. J. Gastroenterol. Hepatol. 32, 1170-1177. doi:10.1111/jgh.13650

Onwubiko, S. N., Nwachukwu, N. Z., Muomah, R. C., Okoloagu, N. M., Ngwegu, O. M., and Nwachukwu, D. C. (2020). Factors associated with depression and anxiety among glaucoma patients in a tertiary hospital South-East Nigeria. Niger. J. Clin. Pract. 23, 315-321. doi: 10.4103/njcp.njcp_140_19.

Ottolini, F., Modena, M. G., and Rigatelli, M. (2005). Prodromal Symptoms in Myocardial Infarction. Psychother. Psychosom. 74, 323-327. doi:10.1159/000086324

Ozturk, S., Yalvac, H. D., Sivri, N., Ozturk, H. M., Kılıc, Y., Bulut, E., et al. (2015). Anxiety and depression scores in patients with coronary artery disease and coronary artery ectasia. Int. J. Cardiol. 186, 299-301. doi:10.1016/j.ijcard.2015.03.305

Pagano, T., Matsutani, L. A., Ferreira, E. A. G., Marques, A. P., and Pereira, C. A. de B. (2004). Assessment of anxiety and quality of life in fibromyalgia patients. Sao Paulo Med. J. 122, 252-258. doi:10.1590/s1516-31802004000600005

Park, E. M., Gelber, S., Rosenberg, S. M., Seah, D. S. E., Schapira, L., Come, S. E., et al. (2018). Anxiety and Depression in Young Women With Metastatic Breast Cancer: A Cross-Sectional Study. Psychosomatics. 59, 251-258. doi:10.1016/j.psym.2018.01.007

Park, K. S., Hwang, S. Y., Choi, B. Y., Kim, J., Kim, S. I., Kim, W.-J., et al. (2021). Associations of depression and anxiety with cardiovascular risk among people living with HIV/AIDS in Korea. Epidemiol. Health. 43, e2021002. doi:10.4178/epih.e2021002

Pence, B. W., Miller, W. C., Whetten, K., Eron, J. J., and Gaynes, B. N. (2006). Prevalence of DSM-IV-Defined Mood, Anxiety, and Substance Use Disorders in an HIV Clinic in the Southeastern United States. J. Acquir. Immune. Defic. Syndr. 42, 298-306. doi:10.1097/01.qai.0000219773.82055.aa

Picardi, A., Porcelli, P., Pasquini, P., Fassone, G., Mazzotti, E., Lega, I., et al. (2006). Integration of Multiple Criteria for Psychosomatic Assessment of Dermatological Patients. Psychosomatics. 47, 122-128. doi:10.1176/appi.psy.47.2.122

Pinto, B., Dhooria, A., Grover, S., Jolly, M., Raj, J. M., and Sharma, A. (2021). Fatigue and its correlates in Indian patients with systemic lupus erythematosus. Clin. Rheumatol. 40, 905-911. doi:10.1007/s10067-020-05445-1

Polatin, P. B., Kinnedy, R. K., Gatchel, R. J., Lillo, E., and Mayer, T. G. (1993). Psychiatric Illness and Chronic Low-Back Pain. Spine, 18, 66-71. doi:10.1097/00007632-199301000-00011

Polikandrioti, M., Koutelekos, I., Vasilopoulos, G., Gerogianni, G., Gourni, M., Zyga, S., et al. (2018). Anxiety and Depression in Patients with Permanent Atrial Fibrillation: Prevalence and Associated Factors. Cardiol. Res. Pract. 2018, 1-9. doi:10.1155/2018/7408129

Pollo, C. F., Miot, H. A., Matos, T. D. de S., Souza, J. M., Jorge, M. F. S., Miot, L. D. B., at el. (2021). Prevalence and factors associated with depression and anxiety in patients with psoriasis. J. Clin. Nurs. 30, 572-580. doi:10.1111/jocn.15577

Ponarovsky, B., Amital, D., Lazarov, A., Kotler, M., and Amital, H. (2011). Anxiety and depression in patients with allergic and non-allergic cutaneous disorders. Int. J. Dermatol. 50, 1217-1222. doi:10.1111/j.1365-4632.2011.04910.x

Pfeuffer, E., Krannich, H., Halank, M., Wilkens, H., Kolb, P., Jany, B., et al. (2017). Anxiety, depression, and health-related QOL in patients diagnosed with PAH or CTEPH. Lung. 195, 759-768. doi:10.1007/s00408-017-0052-z

Preljevic, V. T., Østhus, T. B. H., Sandvik, L., Opjordsmoen, S., Nordhus, I. H., Os, I., et al. (2012). Screening for anxiety and depression in dialysis patients: comparison of the Hospital Anxiety and Depression Scale and the Beck Depression Inventory. J. Psychosom. Res. 73, 139-144. doi:10.1016/j.jpsychores.2012.04.015

Quelhas, R., and Costa, M. (2009). Anxiety, Depression, and Quality of Life in Parkinson’s Disease. J. Neuropsychiatry Clin. Neurosci. 21, 413-419. doi:10.1176/jnp.2009.21.4.413

Qin, S., Tan, Y., Lu, B., Cheng, Y., and Nong, Y. (2018). Survey and analysis for impact factors of psychological distress in HIV-infected pregnant women who continue pregnancy. J. Matern.-Fetal Neonatal. Med. 32, 3160-3167. doi:10.1080/14767058.2018.1459550

Rafanelli, C., Offidani, E., Gostoli, S., and Roncuzzi, R. (2012). Psychological correlates in patients with different levels of hypertension. Psychiatry Res. 198, 154-160. doi:10.1016/j.psychres.2011.09.014

Rahim, R. A., and Cheng, C. H. (2018). Self-reported symptoms of depression, anxiety and stress among patients with Rheumatoid Arthritis in a Malaysian rheumatology centre-prevalence and correlates. Med. J. Malaysia. 73, 226-232.

Raikhy, S., Gautam, S., and Kanodia, S. (2017). Pattern and prevalence of psychiatric disorders among patients attending dermatology OPD. Asian J. Psychiatr. 29, 85-88. doi:10.1016/j.ajp.2017.04.018

Ramsenthaler, C., Gao, W., Siegert, R. J., Edmonds, P. M., Schey, S. A., and Higginson, I. J. (2019). Symptoms and anxiety predict declining health-related quality of life in multiple myeloma: A prospective, multi-centre longitudinal study. Palliat. Med. 33, 541-551. doi:10.1177/0269216319833588

Rani Chadalawada, U., Kumari P, S., Rani M, S., and Devi S, A. (2016). Assessment of anxiety and depression among patients in a government teaching general hospital. J. Evol. Med. Dent. Sci. 5, 4528-4531. doi:10.14260/jemds/2016/1034

Reavley, S., Fisher, A. D., Owen, D., Creed, F. H., and Davis, J. R. E. (1997). Psychological distress in patients with hyperprolactinaemia. Clin. Endocrinol. 47, 343-348. doi:10.1046/j.1365-2265.1997.2701073.x

Rebollo Rubio, A., Morales Asencio, J. M., and Eugenia Pons Raventos, M. (2017). Depression, anxiety and health‐related quality of life amongst patients who are starting dialysis treatment. J. Ren. Care. 43, 73-82. doi:10.1111/jorc.12195

Rezapour, J., Nickels, S., Schuster, A. K., Michal, M., Münzel, T., Wild, P. S., et al. (2018). Prevalence of depression and anxiety among participants with glaucoma in a population-based cohort study: The Gutenberg Health Study. BMC Ophthalmol. 18. doi:10.1186/s12886-018-0831-1

Richards, H. L., Fortune, D. G., Griffiths, C. E., and Main, C. J. (2001). The contribution of perceptions of stigmatisation to disability in patients with psoriasis. J. Psychosom. Res. 50, 11-15. doi:10.1016/s0022-3999(00)00210-5

Robbertz, A. S., Weiss, D. M., Awan, F. T., Byrd, J. C., Rogers, K. A., and Woyach, J. A. (2020). Identifying risk factors for depression and anxiety symptoms in patients with chronic lymphocytic leukemia. Support. Care Cancer. 28, 1799-1807. doi:10.1007/s00520-019-04991-y

Robbins, M. S., Bronheim, R., Lipton, R. B., Grosberg, B. M., Vollbracht, S., Sheftell, F. D., et al. (2011). Depression and Anxiety in Episodic and Chronic Cluster Headache: A Pilot Study. Headache. 52, 600-611. doi:10.1111/j.1526-4610.2011.02024.x

Rogers, H. L., Brotherton, H. T., Plaza, S. L. O., Durán, M. A. S., and Altamar, M. L. P. (2015). Depressive and anxiety symptoms and social support are independently associated with disease-specific quality of life in Colombian patients with rheumatoid arthritis. Rev. Bras. Reumatol. Engl. Ed. 55, 406-413. doi:10.1016/j.rbre.2015.01.005

Romão, A. P. M. S., Gorayeb, R., Romão, G. S., Poli-Neto, O. B., dos Reis, F. J. C., Rosa-e-Silva, J. C., et al. (2009). High levels of anxiety and depression have a negative effect on quality of life of women with chronic pelvic pain. Int. J. Clin. Pract. Suppl.  63, 707-711. doi:10.1111/j.1742-1241.2009.02034.x

Roth, A., Nelson, C. J., Rosenfeld, B., Warshowski, A., O’shea, N., Scher, H., et al. (2006). Assessing Anxiety in Men With Prostate Cancer: Further Data on the Reliability and Validity of the Memorial Anxiety Scale for Prostate Cancer (MAX–PC). Psychosomatics. 47, 340-347. doi:10.1176/appi.psy.47.4.340

Santos, G. R., Boin, I. F. S. F., Pereira, M. I. W., Bonato, T. C. M. P., Silva, R. C. M. A., Stucchi, R. S. B., et al. (2010). Anxiety Levels Observed in Candidates for Liver Transplantation. Transplant. Proc. 42, 513-516. doi:10.1016/j.transproceed.2010.01.009

Savard, J., Laberge, B., Gauthier, J. G., Ivers, H., and Bergeron, M. G. (1998). Evaluating Anxiety and Depression in HIV-Infected Patients. J. Pers. Assess. 71, 349-367. doi:10.1207/s15327752jpa7103_5

Schaefer, C., Chandran, A., Hufstader, M., Baik, R., McNett, M., Goldenberg, D., et al. (2011). The comparative burden of mild, moderate and severe Fibromyalgia: results from a cross-sectional survey in the United States. Health Qual. Life Outcomes. 9, 71. doi:10.1186/1477-7525-9-71

Schlereth, T., Heiland, A., Breimhorst, M., Féchir, M., Kern, U., Magerl, W., et al. (2014). Association between pain, central sensitization and anxiety in postherpetic neuralgia. Eur J Pain. 19, 193-201. doi:10.1002/ejp.537

Schouten, R. W., Haverkamp, G. L., Loosman, W. L., Shaw, P. K. C., van Ittersum, F. J., Smets, Y. F., et al. (2019). Anxiety symptoms, mortality, and hospitalization in patients receiving maintenance Dialysis: a cohort study. Am. J. Kidney Dis. 74, 158-166. doi:10.1053/j.ajkd.2019.02.017

Schramm, C., Wahl, I., Weiler-Normann, C., Voigt, K., Wiegard, C., Glaubke, C., et al. (2014). Health-related quality of life, depression, and anxiety in patients with autoimmune hepatitis. J. Hepatol. 60, 618-624. doi:10.1016/j.jhep.2013.10.035

Schwarz, J., Prashad, A., and Winchester, D. E. (2015). Prevalence and implications of severe anxiety in a prospective cohort of acute chest pain patients. Crit. Pathw. Cardiol. 14, 44-47. doi:10.1097/hpc.0000000000000038

Semaan, V., Noureddine, S., and Farhood, L. (2018). Prevalence of depression and anxiety in end-stage renal disease: A survey of patients undergoing hemodialysis. Appl. Nurs. Res. 43, 80-85. doi:10.1016/j.apnr.2018.07.009

Serber, E. R., Todaro, J. F., Tilkemeier, P. L., and Niaura, R. (2009). Prevalence and characteristics of multiple psychiatric disorders in cardiac rehabilitation patients. J. Cardiopulm. Rehabil. Prev. 29, 161-168. doi:10.1097/hcr.0b013e3181a33365

Sewtz, C., Muscheites, W., Grosse-Thie, C., Kriesen, U., Leithaeuser, M., Glaeser, D., et al. (2021). Longitudinal observation of anxiety and depression among palliative care cancer patients. Ann. Palliat. Med. 10, 3836-3846. doi:10.21037/apm-20-1346

Shafazand, S., Goldstein, M. K., Doyle, R. L., Hlatky, M. A., and Gould, M. K. (2004). Health-related quality of life in patients with pulmonary arterial hypertension. Chest. 126, 1452-1459. doi:10.1378/chest.126.5.1452

Sharma, K., Dhungana, G., Adhikari, S., Bista Pandey, A., and Sharma, M. (2021). Depression and Anxiety among Patients with Type II Diabetes Mellitus in Chitwan Medical College Teaching Hospital, Nepal. Nurs. Res. Pract. 2021, 1-8. doi:10.1155/2021/8846915

Sharma, K., Singh, S., and Remanan, R. (2013a). Quality of life and psychiatric co‑morbidity in Indian migraine patients: A headache clinic sample. Neurol. India. 61, 355. doi:10.4103/0028-3886.117584

Sharma, B. B., Singh, S., Sharma, V. K., Choudhary, M., Singh, V., Lane, S., et al. (2013b). Psychiatric morbidity in chronic respiratory disorders in an Indian service using GMHAT/PC. Gen. Hosp. Psychiatry. 35, 39-44. doi:10.1016/j.genhosppsych.2012.09.009

Shim, E.-J., and Hahm, B.-J. (2011). Anxiety, helplessness/hopelessness and “desire for hastened death” in Korean cancer patients. Eur. J. Cancer Care. 20, 395–402. doi:10.1111/j.1365-2354.2010.01202.x

Smith, E., Gomm, S., and Dickens, C. (2003). Assessing the independent contribution to quality of life from anxiety and depression in patients with advanced cancer. Palliat. Med.17, 509-513. doi:10.1191/0269216303pm781oa

Sonino, N., Fallo, F., and Fava, G. A. (2006). Psychological Aspects of Primary Aldosteronism. Psychother. Psychosom. 75, 327-330. doi:10.1159/000093956

Sonino, N., Navarrini, C., Ruini, C., Ottolini, F., Paoletta, A., Fallo, F., et al. (2004). Persistent Psychological Distress in Patients Treated for Endocrine Disease. Psychother. Psychosom. 73, 78-83. doi:10.1159/000075538

Sonino, N., Tomba, E., Genesia, M. L., Bertello, C., Mulatero, P., Veglio, F., et al. (2011). Psychological Assessment of Primary Aldosteronism: A Controlled Study.
J. Clin. Endocrinol. Metab. 96, E878-E883. doi:10.1210/jc.2010-2723

Soósová, M. S., Macejová, Ž., Zamboriová, M., and Dimunová, L. (2017). Anxiety and depression in Slovak patients with rheumatoid arthritis. J. Ment. Health. 26, 21-27. doi:10.1080/09638237.2016.1244719

Stark, D., Kiely, M., Smith, A., Velikova, G., House, A., and Selby, P. (2002). Anxiety Disorders in Cancer Patients: Their Nature, Associations, and Relation to Quality of Life. J. Clin. Oncol. 20, 3137–3148. doi:10.1200/jco.2002.08.549

Staubach, P., Eckhardt-Henn, A., Dechene, M., Vonend, A., Metz, M., Magerl, M., et al. (2005). Quality of life in patients with chronic urticaria is differentially impaired and determined by psychiatric comorbidity. Br. J. Dermatol. 154, 294–298. doi:10.1111/j.1365-2133.2005.06976.x

Stewart, B., Mikocka-Walus, A., Morgan, J., Colman, A., Phelps, M., Harley, H., et al. (2012). Anxiety and depression in Australian chronic hepatitis C outpatients: prevalence and predictors. Australas. Psychiatry. 20, 496-500. doi:10.1177/1039856212460597

Strik, J. J., Denollet, J., Lousberg, R., and Honig, A. (2003). Comparing symptoms of depression and anxiety as predictors of cardiac events and increased health care consumption after myocardial infarction. J. Am. Coll. Cardiol. 42, 1801-1807. doi:10.1016/j.jacc.2003.07.007

Sun, N., Lou, P., Shang, Y., Zhang, P., Wang, J., Chang, G., et al. (2016). Prevalence and determinants of depressive and anxiety symptoms in adults with type 2 diabetes in China: a cross-sectional study. BMJ Open. 6, e012540. doi:10.1136/bmjopen-2016-012540

Sun, W., Wu, M., Qu, P., Lu, C., and Wang, L. (2014). Psychological well-being of people living with HIV/AIDS under the new epidemic characteristics in China and the risk factors: a population-based study. Int. J. Infect. Dis. 28, 147–152. doi:10.1016/j.ijid.2014.07.010

Sutton, K., Cooper, M., Pimm, J., and Wallace, L. (1999). Anxiety in chronic obstructive pulmonary disease: the role of illness specific catastrophic thoughts. Cognit. Ther. Res. 23, 573-585. doi:10.1023/a:1018780623406

Suzuki, M., Deno, M., Myers, M., Asakage, T., Takahashi, K., Saito, K., et al. (2016). Anxiety and depression in patients after surgery for head and neck cancer in Japan. Palliat. Support. Care, 14, 269–277. doi:10.1017/s1478951515000930

Tagay, S., Herpertz, S., Langkafel, M., Erim, Y., Bockisch, A., Senf, W., et al. (2006). Health-related Quality of Life, Depression and Anxiety in Thyroid Cancer Patients. Qual. Life Res. 15, 695–703. doi:10.1007/s11136-005-3689-7

Takita, Y., Takeda, Y., Fujisawa, D., Kataoka, M., Kawakami, T., and Doorenbos, A. Z. (2021). Depression, anxiety and psychological distress in patients with pulmonary hypertension: a mixed-methods study. BMJ Open Respir. Res. 8, e000876. doi:10.1136/bmjresp-2021-000876

Tang, W. K., Wong, E., Chiu, H. F., Lum, C. M., and Ungvari, G. S. (2008). Examining item bias in the anxiety subscale of the Hospital Anxiety and Depression Scale in patients with chronic obstructive pulmonary disease. Int. J. Methods Psychiatr. Res. 17, 104-110. doi:10.1002/mpr.234

Tat, T. S. (2019). Higher Levels of Depression and Anxiety in Patients with Chronic Urticaria. Med. Sci. Monit. 25, 115-120. doi:10.12659/msm.912362

Teixeira, R. B., Marins, J. C. B., de Sá-Junior, A. R., de Carvalho, C. J., Lade, C. G., Rizvanov, A. A., et al. (2015). Psychological and Cognitive Profile of Hypertensive and Diabetic Patients. J. Nerv. Ment. Dis. 203, 781-785. doi:10.1097/nmd.0000000000000367

Ter Kuile, M. M., Weijenborg, P. T. M., and Spinhoven, P. (2010). PAIN: Sexual Functioning in Women with Chronic Pelvic Pain: The Role of Anxiety and Depression. J. Sex. Med. 7, 1901-1910. doi:10.1111/j.1743-6109.2009.01414.x

Thapa, N., Maharjan, M., Shrestha, T. M., Gauchan, S., Pun, P., and Thapa, Y. B. (2017). Anxiety and depression among patients with chronic obstructive pulmonary disease and general population in rural Nepal. BMC Psychiatry. 17, 397. doi:10.1186/s12888-017-1550-5

Thijssen, A. Y., Jonkers, D. M., Leue, C., van der Veek, P. P., Vidakovic-Vukic, M., van Rood, Y. R., et al. (2010). Dysfunctional cognitions, anxiety and depression in irritable bowel syndrome. J. Clin. Gastroenterol. 44, e236-e241. doi:10.1097/mcg.0b013e3181eed5d8

Tian, Z., Huang, Y., Yue, T., Zhou, J., Tao, L., Han, L., et al. (2018). A Chinese cross-sectional study on depression and anxiety symptoms in patients with psoriasis vulgaris. Psychol. Health Med. 24, 269-280. doi:10.1080/13548506.2018.1529323

Todaro, J. F., Shen, B. J., Raffa, S. D., Tilkemeier, P. L., and Niaura, R. (2007). Prevalence of anxiety disorders in men and women with established coronary heart disease. J. Cardiopulm. Rehabil. Prev. 27, 86-91. doi:10.1097/01.hcr.0000265036.24157.e7

Tomazoni, E. I., and Benvegnú, D. M. (2018). Symptoms of anxiety and depression, and quality of life of patients with Crohn’s disease. **Arq. Gastroenterol.** 55, 148-153. doi:10.1590/s0004-2803.201800000-26

Tosic-Golubovic, S., Miljkovic, S., Nagorni, A., Lazarevic, D., and Nikolic, G. (2010). Irritable bowel syndrome, anxiety, depression and personality characteristics. Psychiatr. Danub. 22, 418-424.

Treudler, R., Zeynalova, S., Riedel‐Heller, S. G., Zuelke, A. E., Roehr, S., Hinz, A., et al. (2020). Depression, anxiety and quality of life in subjects with atopic eczema in a population‐based cross‐sectional study in Germany. J. Eur. Acad. Dermatol. Venereol. 34, 810-816. doi:10.1111/jdv.16148

Truong, D. V., Bui, Q. T. T., Nguyen, D. T., and Moore, J. (2019). Anxiety Among Inpatients With Cancer: Findings From a Hospital-Based Cross-Sectional Study in Vietnam. Cancer Control. 26, 107327481986464. doi:10.1177/1073274819864641

Turan, O., Yemez, B., and Itil, O. (2014). The effects of anxiety and depression symptoms on treatment adherence in COPD patients. Prim. Health Care Res. Dev. 15, 244-251. doi:10.1017/s1463423613000169

Turkistani, I., Nuqali, A., Badawi, M., Taibah, O., Alserihy, O., Morad, M., et al. (2014). The prevalence of anxiety and depression among end-stage renal disease patients on hemodialysis in Saudi Arabia. Ren. Fail. 36, 1510-1515. doi:10.3109/0886022x.2014.949761

Uçar, M., Sarp, Ü., Karaaslan, Ö., Gül, A. I., Tanik, N., and Arik, H. O. (2015). Health anxiety and depression in patients with fibromyalgia syndrome. Int. J. Med. Res. 43, 679-685. doi:10.1177/0300060515587578

Uchmanowicz, I., Jankowska-Polanska, B., Motowidlo, U., Uchmanowicz, B., and Chabowski, M. (2016). Assessment of illness acceptance by patients with COPD and the prevalence of depression and anxiety in COPD. Int. J. Chron. Obstruct. Pulmon. Dis. 11, 963-970. doi:10.2147/copd.s102754

Uhlenbusch, N., Löwe, B., Härter, M., Schramm, C., Weiler-Normann, C., and Depping, M. K. (2019). Depression and anxiety in patients with different rare chronic diseases: A cross-sectional study. PLoS One. 14, e0211343. doi:10.1371/journal.pone.0211343

Unseld, M., Krammer, K., Lubowitzki, S., Jachs, M., Baumann, L., Vyssoki, B., et al. (2019). Screening for post‐traumatic stress disorders in 1017 cancer patients and correlation with anxiety, depression, and distress. Psycho-Oncol. 28, 2382-2388. doi: 10.1002/pon.5239.

Valença, A. M., Falcão, R., Freire, R. C., Nascimento, I., Nascentes, R., Zin, W. A., et al. (2006). The relationship between the severity of asthma and comorbidites with anxiety and depressive disorders. Braz. J. Psychiatry. 28, 206-208. doi:10.1590/s1516-44462006005000005

Vamos, M., and Kolbe, J. (1999). Psychological factors in severe chronic asthma. Aust. N. Z. J. Psychiatry. 33, 538-544. doi:10.1080/j.1440-1614.1999.00591.x

Van den Brekel, L., van der Baan, F. H., Zweers, D., Koldenhof, J. J., Vos, J. B. H., de Graeff, A., et al. (2020). Predicting Anxiety in Hospitalized Cancer Patients. J. Pain Symptom Manage. 60, 522-530.e1. doi:10.1016/j.jpainsymman.2020.04.005

Van den Heuvel, L., Chishinga, N., Kinyanda, E., Weiss, H., Patel, V., Ayles, H., et al. (2013). Frequency and correlates of anxiety and mood disorders among TB- and HIV-infected Zambians. AIDS Care. 25, 1527-1535. doi:10.1080/09540121.2013.793263

Vanhoof, J. M., Delcroix, M., Vandevelde, E., Denhaerynck, K., Wuyts, W., Belge, C., et al. (2014). Emotional symptoms and quality of life in patients with pulmonary arterial hypertension. J. Heart Lung Transplant. 33, 800-808. doi:10.1016/j.healun.2014.04.003

Vikjord, S. A. A., Brumpton, B. M., Mai, X. M., Vanfleteren, L., and Langhammer, A. (2020). The association of anxiety and depression with mortality in a COPD cohort. The HUNT study, Norway. Respir. Med. 171, 106089. doi: 10.1016/j.rmed.2020.106089

Vistad, I., Cvancarova, M., Kristensen, G. B., and Fosså, S. D. (2011). A study of chronic pelvic pain after radiotherapy in survivors of locally advanced cervical cancer. J. Cancer Surviv. 5, 208-216. doi:10.1007/s11764-011-0172-z

Vogt, F., Sahota, J., Bidder, T., Livingston, R., Bellas, H., Gane, S. B., et al. (2021). Chronic rhinosinusitis with and without nasal polyps and asthma: Omalizumab improves residual anxiety but not depression. Clin. Transl. Allergy. 11. doi:10.1002/clt2.12002

Vojvodić, A., and Dedic, G. (2020). Depression, anxiety and quality of life in patients with melanoma. Military Medical and Pharmaceutical Journal of Serbia, 77, 1318-1322. doi:10.2298/vsp180414185v

Von Leupoldt, A., Taube, K., Lehmann, K., Fritzsche, A., and Magnussen, H. (2011). The impact of anxiety and depression on outcomes of pulmonary rehabilitation in patients with COPD. Chest. 140, 730-736. doi: 10.1378/chest.10-2917

Wallis, O., Bol, Y., Köhler, S., and Heugten, C. (2019). Anxiety in multiple sclerosis is related to depressive symptoms and cognitive complaints. Acta Neurol. Scand. 141, 212-218. doi:10.1111/ane.13191

Wan, S., He, H.-G., Mak, A., Lahiri, M., Luo, N., Cheung, P. P., et al. (2015). AB1211-HPR Health-Related Quality of Life and its Predictors Among Patients with Rheumatoid Arthritis. Ann. Rheum. Dis. 74, 1339.3-1340. doi:10.1136/annrheumdis-2015-eular.1223

Wang, G., Cui, J., Wang, Y., Deng, B., Liang, X., Bai, J., et al. (2013a). Anxiety and Adverse Coronary Artery Disease Outcomes in Chinese Patients. Psychosom. Med. 75, 530-536. doi:10.1097/psy.0b013e3182984317

Wang, Y., Zou, L., Jiang, M., Wei, Y., and Jiang, Y. (2013b). Measurement of distress in Chinese inpatients with lymphoma. Psychooncology. 22, 1581-1586. doi:10.1002/pon.3170

Wasan, A., Fernandez, E., Jamison, R. N., and Bhattacharyya, N. (2007). Association of anxiety and depression with reported disease severity in patients undergoing evaluation for chronic rhinosinusitis. Ann. Otol. Rhinol. Laryngol. 116, 491-497. doi:10.1177/000348940711600703

Watad, A., Bragazzi, N. L., Adawi, M., Aljadeff, G., Amital, H., Comaneshter, D., et al. (2017). Anxiety disorder among rheumatoid arthritis patients: insights from real-life data. J. Affect. Disord. 213, 30-34. doi:10.1016/j.jad.2017.02.007

Weaver, L. J., and Madhu, S. V. (2015). Type 2 Diabetes and Anxiety Symptoms Among Women in New Delhi, India. Am. J. Public Health. 105, 2335-2340. doi:10.2105/ajph.2015.302830

Wei, C.-B., Jia, J.-P., Wang, F., Zhou, A.-H., Zuo, X.-M., and Chu, C.-B. (2016). Overlap between Headache, Depression, and Anxiety in General Neurological Clinics. Chin. Med. J. 129, 1394-1399. doi:10.4103/0366-6999.183410

White, J., Hopkins, R. O., Glissmeyer, E. W., Kitterman, N., and Elliott, C. G. (2006). Cognitive, emotional, and quality of life outcomes in patients with pulmonary arterial hypertension. Respir. Res. 7, 55. doi:10.1186/1465-9921-7-55

Wicks, P., Abrahams, S., Masi, D., Hejda-Forde, S., Leigh, P. N., and Goldstein, L. H. (2007). Prevalence of depression in a 12-month consecutive sample of patients with ALS. Eur. J. Neurol. 14, 993-1001. doi:10.1111/j.1468-1331.2007.01843.x

Williamson, T. J., Ostroff, J. S., Haque, N., Martin, C. M., Hamann, H. A., Banerjee, S. C., et al. (2020). Dispositional shame and guilt as predictors of depressive symptoms and anxiety among adults with lung cancer: The mediational role of internalized stigma. Stigma and Health, 5, 425-433. doi:10.1037/sah0000214

Wilson, K. G., Chochinov, H. M., Graham Skirko, M., Allard, P., Chary, S., Gagnon, P. R., et al. (2007). Depression and Anxiety Disorders in Palliative Cancer Care. J. Pain Symptom Manage. 33, 118-129. doi:10.1016/j.jpainsymman.2006.07.016

Woon, L. S. C., Sidi, H. B., Ravindran, A., Gosse, P. J., Mainland, R. L., Kaunismaa, E. S., et al. (2020). Depression, anxiety, and associated factors in patients with diabetes: evidence from the anxiety, depression, and personality traits in diabetes mellitus (ADAPT-DM) study. BMC Psychiatry. 20, 1-14. doi:10.21203/rs.2.20390/v1

Wu, S.-F. V., Huang, Y.-C., Lee, M.-C., Wang, T.-J., Tung, H.-H., and Wu, M.-P. (2013). Self-efficacy, self-care behavior, anxiety, and depression in Taiwanese with type 2 diabetes: A cross-sectional survey. Nurs. Health Sci. 15, 213-219. doi:10.1111/nhs.12022

Xiao, T., Qiu, H., Chen, Y., Zhou, X., Wu, K., Ruan, X., et al. (2018). Prevalence of anxiety and depression symptoms and their associated factors in mild COPD patients from community settings, Shanghai, China: a cross-sectional study. BMC Psychiatry. 18, 89. doi:10.1186/s12888-018-1671-5

Yadav, R., Yadav, P., Kumar, S. S., and Kumar, R. (2021). Assessment of Depression, Anxiety, and Sleep Disturbance in COVID-19 Patients at Tertiary Care Center of North India. J. Neurosci. Rural. Pract. 12, 316-322. doi:10.1055/s-0040-1722811

Yan, X., Chen, X., Li, M., and Zhang, P. (2019). Prevalence and risk factors of anxiety and depression in Chinese patients with lung cancer: a cross-sectional study. Cancer Manag. Res. 11, 4347-4356. doi:10.2147/cmar.s202119

Yang, Y., Ding, R., Hu, D., Zhang, F., and Sheng, L. (2014). Reliability and validity of a Chinese version of the HADS for screening depression and anxiety in psycho-cardiological outpatients. Compr. Psychiatry. 55, 215-220. doi:10.1016/j.comppsych.2013.08.012

Yang, X. J., Jiang, H. M., Hou, X. H., and Song, J. (2015). Anxiety and depression in patients with gastroesophageal reflux disease and their effect on quality of life. World J. Gastroenterol. 21, 4302-4309. doi:10.3748/wjg.v21.i14.4302

Yıldırım, N. K., Özkan, M., Özkan, S., Oflaz, S. B., Gelincik, A., and Büyüköztürk, S. (2012). Relationship among alexithymia, anxiety, and depression in patients with chronic idiopathic urticaria. Nobel Med. 8, 46-51.

Yilmaz-Oner, S., Oner, C., Dogukan, F. M., Moses, T. F., Demir, K., Tekayev, N., et al. (2015). Anxiety and depression predict quality of life in Turkish patients with systemic lupus erythematosus. Clin. Exp. Rheumatol. 33, 360-365. doi:10.1007/s10067-015-2930-1

Yon, M., Gumusyayla, S., and Vural, G. (2020). Sleep quality and frequency of comorbidities and its relation with chronic migraine related disability in patients with chronic migraine. Ann. Med. Res. 27, 45. doi:10.5455/annalsmedres.2019.12.879

Yuan, L., Pan, B., Wang, W., Wang, L., Zhang, X., and Gao, Y. (2020). Prevalence and Predictors of Anxiety and Depressive Symptoms among Patients Diagnosed with Oral Cancer in China: A cross-sectional study. BMC Psychiatry. 20, 394. doi:10.21203/rs.3.rs-15838/v2

Zamora-Racaza, G., Azizoddin, D. R., Ishimori, M. L., Ormseth, S. R., Wallace, D. J., Penserga, E. G., et al. (2017). Role of psychosocial reserve capacity in anxiety and depression in patients with systemic lupus erythematosus. Int. J. Rheum. Dis. 21, 850-858. doi:10.1111/1756-185x.13033

Zebenholzer, K., Lechner, A., Broessner, G., Lampl, C., Luthringshausen, G., Wuschitz, A., et al. (2016). Impact of depression and anxiety on burden and management of episodic and chronic headaches – a cross-sectional multicentre study in eight Austrian headache centres. J. Headache Pain. 17. doi:10.1186/s10194-016-0603-3

Zhang, A. Z., Wang, Q. C., Huang, K. M., Huang, J. G., Zhou, C. H., Sun, F. Q., et al. (2016). Prevalence of depression and anxiety in patients with chronic digestive system diseases: A multicenter epidemiological study. World J. Gastroenterol. 22, 9437-9444. doi:10.3748/wjg.v22.i42.9437

Zhang, L., Xia, Y., Zhang, Q., Fu, T., Yin, R., Guo, G., et al. (2017). The correlations of socioeconomic status, disease activity, quality of life, and depression/anxiety in Chinese patients with rheumatoid arthritis. Psychol. Health Med. 22, 28-36. doi:10.1080/13548506.2016.1198817

Zhang, M., Kim, J. C., Li, Y., Shapiro, B. B., Porszasz, J., Bross, R., et al. (2014). Relation between anxiety, depression, and physical activity and performance in maintenance hemodialysis patients. J. Ren. Nutr. 24, 252-260. doi:10.1053/j.jrn.2014.03.002

Zhang, Z., Feng, Y., Song, R., Yang, D., and Duan, X. (2021). Prevalence of psychiatric diagnosis and related psychopathological symptoms among patients with COVID-19 during the second wave of the pandemic. Glob. Health. 17. doi:10.1186/s12992-021-00694-4

Zhang, J., Yang, Z., Wang, X., Li, J., Dong, L., Wang, F., et al. (2020). The relationship between resilience, anxiety and depression among patients with mild symptoms of COVID‐19 in China: A cross‐sectional study. J. Clin. Nurs. 29, 4020-4029. doi:10.1111/jocn.15425

Zhong, R., Chen, Q., Li, M., Li, N., Chu, C., Li, J., et al. (2021). A cross-sectional study on the association of serum uric acid levels with depressive and anxiety symptoms in people with epilepsy. BMC Psychiatry. 21. doi:10.1186/s12888-020-03019-8

Zhou, C., Qian, S., Wu, P., and Qiu, C. (2013). Anxiety and depression in Chinese patients with glaucoma: Sociodemographic, clinical, and self-reported correlates. J. Psychosom. Res. 75, 75-82. doi:10.1016/j.jpsychores.2013.03.005

Zhou, F., Wang, R.-R., Huang, H.-P., Du, C.-L., Wu, C.-M., Qian, X.-M., et al. (2021). A randomized trial in the investigation of anxiety and depression in patients with coronavirus disease 2019 (COVID-19). Ann. Palliat. Med. 10, 2167-2174. doi:10.21037/apm-21-212

Zhu, K., van Hilten, J. J., and Marinus, J. (2017). Onset and evolution of anxiety in Parkinson's disease. Eur. J. Neurol. 24, 404-411. doi:10.1111/ene.13217
